# CODE-II: a large-scale dataset for artificial intelligence in ECG analysis

**DOI:** 10.1038/s41746-026-02704-4

**Published:** 2026-05-26

**Authors:** Petrus E. O. G. B. Abreu, Gabriela M. M. Paixão, Jiawei Li, Paulo R. Gomes, Peter W. Macfarlane, Ana C. S. Oliveira, Vinícius T. Carvalho, Thomas B. Schön, Antonio Luiz P. Ribeiro, Antônio H. Ribeiro

**Affiliations:** 1 Faculdade de Medicina, Universidade Federal de Minas Gerais (UFMG), Belo Horizonte, Brazil; 2 Uppsala University, Uppsala, Sweden; 3 Telehealth Center, Hospital das Clínicas, UFMG, Belo Horizonte, Brazil; 4 University of Glasgow, Glasgow, UK

**Keywords:** Cardiology, Computational biology and bioinformatics, Diseases, Health care, Medical research

## Abstract

Data-driven methods for electrocardiogram (ECG) interpretation are rapidly progressing. Large datasets have enabled advances in artificial intelligence (AI) based ECG analysis, yet limitations in annotation quality, size, and scope remain major challenges. Here we present CODE-II, a large-scale real-world dataset of 2,735,269 12-lead ECGs from 2,093,807 adult patients collected by the Telehealth Network of Minas Gerais (TNMG), Brazil. Each exam was annotated using standardized diagnostic criteria and reviewed by cardiologists. A defining feature of CODE-II is a set of 66 clinically meaningful diagnostic classes, developed with cardiologist input and routinely used in telehealth practice. We additionally provide an openly available subset: CODE-II-open, a public subset of 15,000 patients, and the CODE-II-test, a non-overlapping set of 8,475 exams reviewed by multiple cardiologists for blinded evaluation. A neural network pre-trained on CODE-II achieved superior transfer performance on external benchmarks (PTB-XL and CPSC 2018) and outperformed alternatives trained on larger datasets.

## Introduction

Cardiovascular diseases are the leading cause of death worldwide, causing about 17.9 million deaths annually^1^. The electrocardiogram (ECG) is a simple, non-invasive, low-cost, and widely available tool for screening and monitoring cardiovascular conditions. Despite its broad accessibility, manual interpretation requires highly skilled and well-trained cardiologists, which can be a limiting factor in many healthcare settings. Automated ECG interpretation has a history spanning more than six decades, with pioneering computer-based analyses introduced in the 1960s. While widely used in practice, the modest performance of rule-based algorithms limits their clinical usage as a tool and relegates them to an ancillary role^2^. In recent years, however, the convergence of large-scale data and artificial intelligence (AI) has transformed the field. Deep neural networks now achieve cardiologist-level performance in clinically relevant diagnostic tasks, including the detection of six abnormalities^3^, and have further enabled novel applications beyond traditional interpretation, such as Chagas disease screening^4^, atrial fibrillation risk prediction^5^, and AI-derived ECG age as a predictor of mortality risk^6^. These advances underscore the transformative potential of AI-based ECG analysis, while also highlighting the critical need for large, well-annotated datasets with clinically meaningful labels to support further progress.

There is an intrinsic connection between telehealth and AI. Telehealth generates large volumes of data required to train AI algorithms, and its fully digital format provides an ideal setting for integrating AI-based tools to enhance clinical workflows. The Telehealth Network of Minas Gerais (TNMG)^7^ is a consolidated public tele-electrocardiography (tele-ECG) service in operation since 2005, currently providing more than 8000 ECG reports daily to over 1400 counties across 14 states in Brazil. A total of more than 11 million digital ECGs have been performed (see https://telessaude.hc.ufmg.br). From this service, the CODE dataset (CODE-I)^3^ was established, comprising ECGs and annotations provided by doctors in the TNMG from 2010 to 2017. This dataset has been linked to mortality data, the Clinical Outcomes in Digital Electrocardiography (CODE) study^8^, with many publications regarding the prognostic value of the ECG, using both standard ECG classification and AI-based methods^6,9–13^.

Computerized ECG analysis has been data-driven since its inception in the 1960s, although the availability of large, high-quality datasets emerged only later. Landmark resources shaped the field, from early cohorts such as the MIT-BIH Arrhythmia Database^14^ to more recent large-scale datasets like the PTB-XL^15^ and the Harvard–Emory^16^. The CODE dataset^3^ shaped the field in its own way. It comprises more than 2 million ECGs from over 1 million patients. Published in 2020, it is now available for more than 70 research groups. A subset of it, the CODE-15%, was made completely open and was downloaded more than 86 thousand times by the time of the writing of this paper. The models pre-trained on it (also made openly available at https://github.com/antonior92/automatic-ecg-diagnosis) had a huge impact on research and have been downloaded thousands of times.

One of the main limitations of the original CODE dataset was the restricted number of annotated abnormalities. We focused on six representative classes, which were extracted from free-text medical reports and underwent several rounds of revision to ensure consistency. However, the effort required to curate these six classes made it infeasible, at the time, to extend the process to all abnormalities routinely considered by the telehealth center. Thanks to major improvements in the center’s internal operations, such as standardizing report formats and resolving annotation inconsistencies^7^, CODE-II now offers high-quality labels for comprehensive abnormality classes.

The heterogeneity of ECG reports has been an issue since the inception of epidemiological studies in the 1930s. The lack of standardization in ECG classes has impaired comparisons between different populations worldwide. In this setting, ECG classification systems were developed, such as the Minnesota Code^17^ and NovaCode^18^, but their use was primarily limited to research, with limitations in clinical practice. The last ECG statement issued by the American Heart Association (AHA) and the American College of Cardiology (ACC) and endorsed by the Heart Rhythm Society and the International Society for Computerized Electrocardiology was released in 2007 to examine the relation of the resting ECG to its technology, increase understanding of how the modern ECG is derived and recorded, and promote standards that will improve the accuracy and usefulness of the ECG in practice^19^. Since then, to the best of our knowledge, no other comprehensive international guideline on ECG reporting has been published.

With over 40 cardiologists involved in the TNMG tele-ECG service, standardizing the ECG report was essential to ensure consistent, high-quality, and reliable interpretations. This standardization had to balance two priorities: alignment with internationally accepted criteria and adaptation to Brazil’s regional and epidemiological context, for instance, accounting for the high prevalence of Chagas disease in certain states. To this end, the ECG CODE classes were defined based on the 2007 guidelines from the AHA and the ACC^19^, and the Brazilian guideline for reporting ECG^20,21^. Moreover, AI-based classification within the tele-ECG workflow may help prioritize urgent cases with life-threatening abnormalities, improving the efficiency of the reporting process. The CODE-II dataset, collected between January 2019 and December 2022, fully benefits from this standardized approach, enabling high-quality annotation across a wide range of abnormalities.

Our contributions are: (1) we curate and describe a large-scale ECG database: the CODE-II dataset, (2) we describe the classification system used in the telehealth service, (3) we develop a deep neural network-based classification model using this dataset, showcasing its utility for AI applications, (4) we offer a subset of this dataset, the CODE-II-open, as an open public benchmark for AI-based ECG classification models, with annotations derived from the 66 expert-defined CODE diagnostic classes, and (5) we provide a high-quality test set, the CODE-II-test, containing 66 ECG classes and annotated by multiple cardiologists, to support the evaluation of AI algorithms. The proposed CODE classes are clinically meaningful and well-suited for AI-based ECG analysis. This resource also facilitates comparisons with other electronic cohorts and encourages international collaboration in ECG research. The high-quality data will serve as a valuable foundation for developing robust AI models for automated ECG interpretation.

## Results

### CODE-II dataset

CODE-II is a large-scale, real-world dataset of 12-lead ECG exams collected and annotated by the TNMG from January 2019 to December 2022, originally comprising over 3 million exams. After applying the filtering and quality-control procedures described in the “Methods” section, a curated set of 2,735,269 exams from 2,093,807 unique patients was retained. This curated set constitutes the CODE-II dataset.

**Fig. 1:**
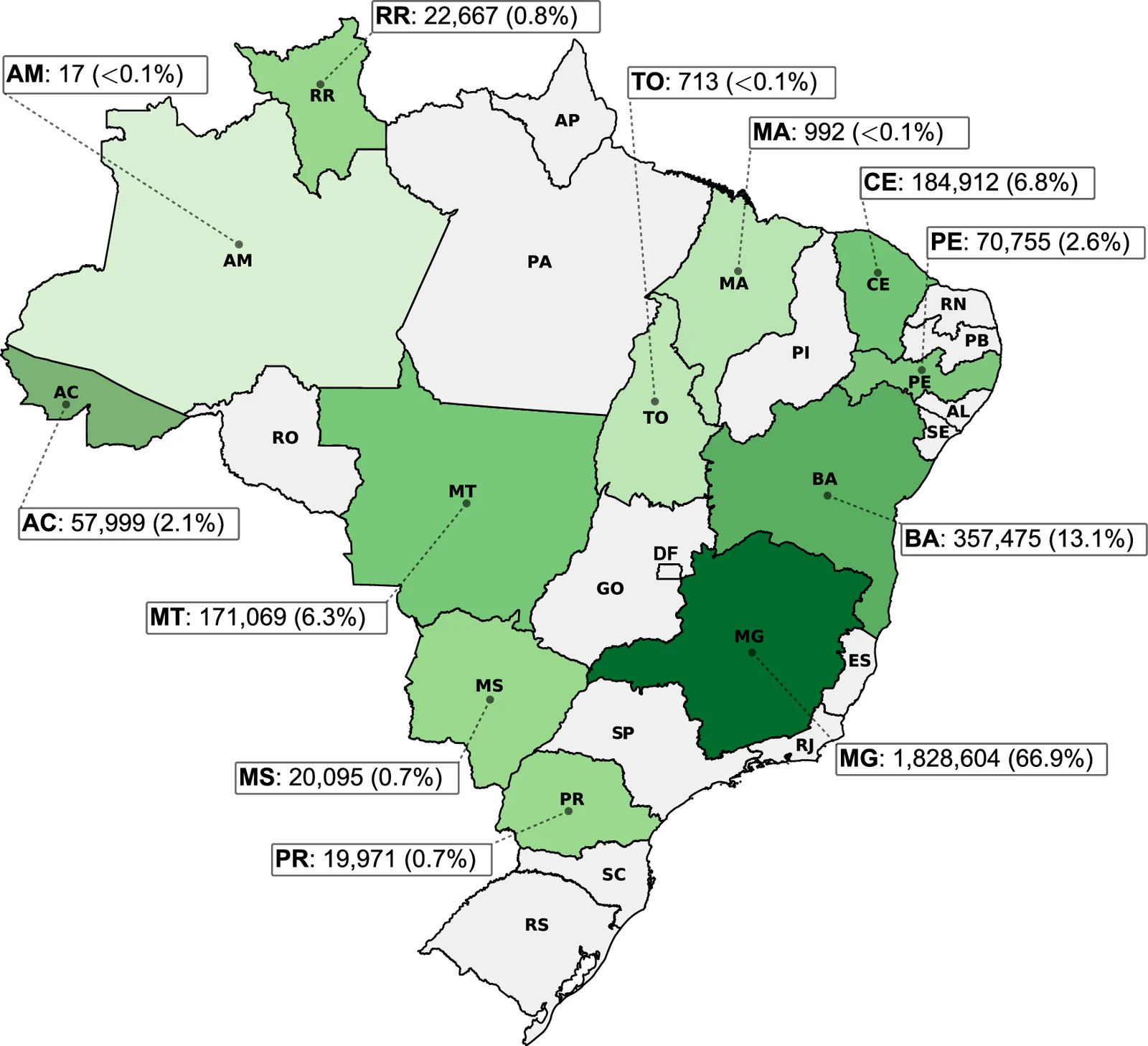
Map of Brazil illustrating the absolute and relative numbers of ECGs for each state served by TNMG in the CODE-II dataset. Basemap polygons (Brazil state boundaries; brazil-states.geojson) were obtained from the Click That 'Hood project (MIT License). The figure was generated using Python/GeoPandas and finalized using Inkscape.

During the CODE-II collection period, TNMG’s tele-ECG service supported 12 Brazilian states, with exam volumes across states shown in Fig. 1. Based on the indications provided by the healthcare professionals who submitted the exams to the TNMG analysis center, approximately 73.2% of the exams were related to elective cases, 25.3% to urgent cases, and 1.5% to preferential cases. These exams were collected mainly in primary healthcare centers, followed by hospitals, emergency departments, and ambulances.

Among the 2,093,807 patients, 59.1% (1,237,632) were female, and 40.9% (856,175) were male. As some patients underwent multiple exams—1,663,122 had a single ECG, and 430,685 had two or more ECGs (Supplementary Fig. 4a)—only the first exam per patient was considered in analyses involving clinical or demographic characterization of the population. The overall mean age was 53.6 years, with a mean of 53.0 years for female patients and 54.4 years for male patients.

Self-reported clinical comorbidities of patients, categorized by age (18–59 years and 60 years or older at the time of the exam) and sex, are summarized in Supplementary Table 1. Hypertension was the most prevalent comorbidity, affecting nearly half of the study population (47.7%), followed by diabetes mellitus (14.0%), smoking (8.2%), dyslipidemia (4.8%), previous myocardial infarction (1.8%), Chagas disease (1.1%), and chronic obstructive pulmonary disease (0.7%). Patients could report more than one comorbidity, and medication usage was also considered to infer the presence of hypertension, dyslipidemia, and diabetes, as detailed in the Supplementary Materials.

In addition to these comorbidities, clinical indications for ECG exams were recorded using checkbox fields, allowing multiple symptoms to be reported per patient. The most frequent reasons were routine examinations and chest pain, followed by preoperative risk assessment, palpitations, dyspnea, and “other.” The full distribution of clinical indications is presented in Supplementary Fig. 3. Notably, routine exams and chest pain were the leading causes across all demographic groups, with younger female patients (aged 18–59) showing the highest proportions across all categories. A more granular characterization and further descriptive details of the CODE-II dataset, including extended tables and figures on patient- and exam-level attributes, are provided in the Supplementary Materials.

### The CODE diagnostic classes and their distribution in the population

Each ECG in the CODE-II dataset was annotated by a certified cardiologist with one or more diagnostic labels from a standardized set of 66 classes, known as the CODE diagnostic classes, of which 65 are not mutually exclusive, and 1 (Normal case) is assigned exclusively. These classes were developed to ensure consistency and clinical relevance across all ECG reports, enabling the analysis of cardiovascular findings on a large scale. As described in the “Methods” section, these labels encompass a wide spectrum of normal and abnormal findings. They can be grouped into 10 clinically meaningful categories: Pacemaker, Normal, Technical issues, Sinus Rhythm, Arrhythmia, Atrioventricular Conduction Disorders, Chamber Hypertrophy, Intraventricular Conduction Disturbances, Ischemia/Infarction, and Miscellaneous Conditions.

**Fig. 2:**
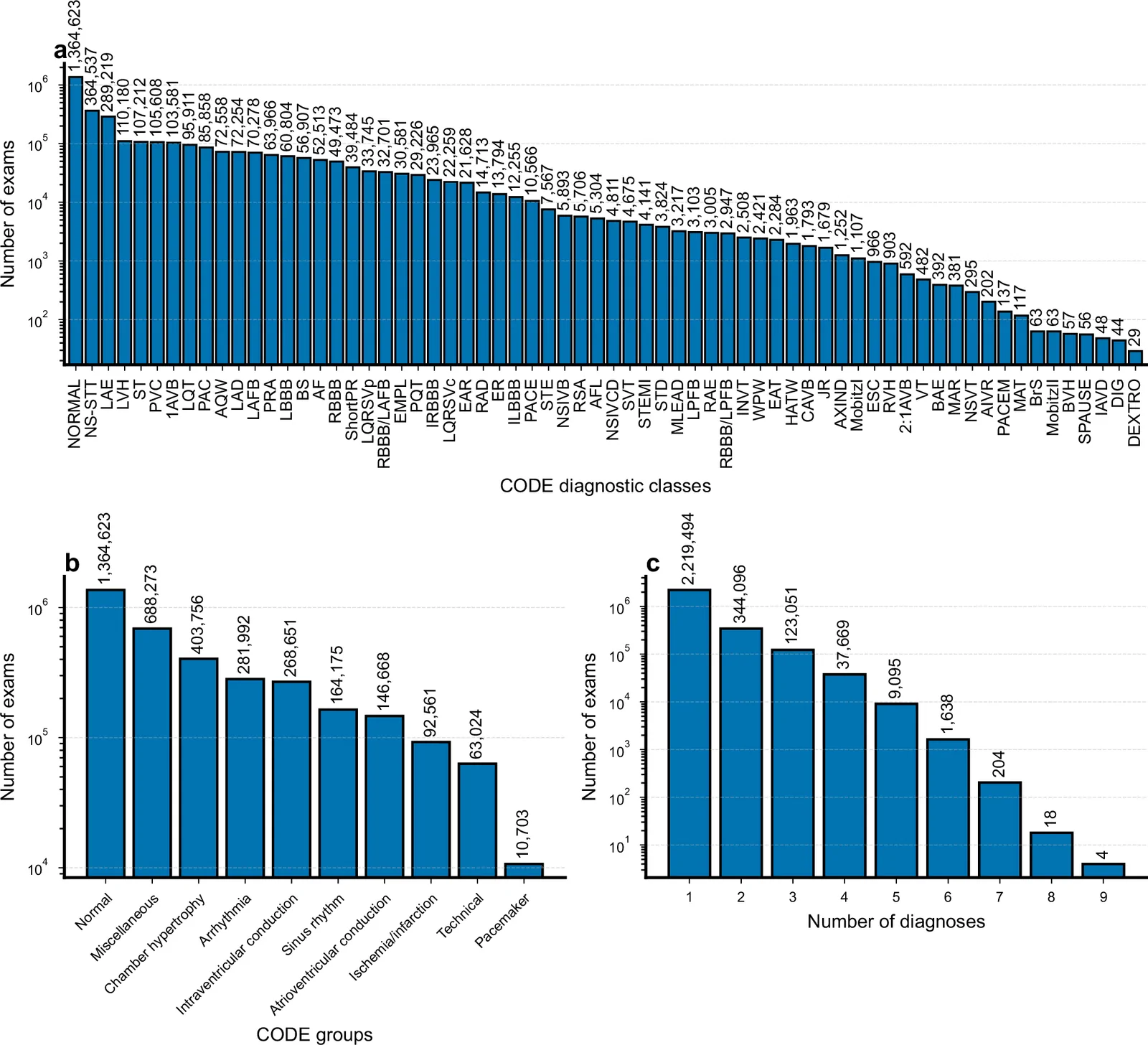
Summary of diagnostic class frequencies and group combinations in CODE-II. **a** Number of ECG exams associated with each diagnostic class in the CODE-II dataset. Diagnostic classes are not mutually exclusive; multiple diagnoses may be assigned to a single exam, except for Normal ECGs, which are exclusive. **b** Number of exams for each CODE diagnostic group. Exams may belong to more than one group. **c** Distribution of the number of diagnostic classes assigned per exam. *All panels use a logarithmic scale on the y-axis*.

Figure 2a shows the distribution of all 66 CODE diagnostic classes according to their frequency in the dataset. For visualization purposes, each class is referenced by a shorthand, hereafter referred to as the CODE label; the corresponding formal diagnostic statements appear in Fig. 8, and the label–statement mapping is given in Supplementary Table 2. The most common findings included normal ECG (NORMAL: 1,364,623 exams, 49.9%), nonspecific ST-T abnormality (NS-STT: 364,537 exams, 13.3%), left atrial enlargement (LAE: 289,219 exams, 10.6%), left ventricular hypertrophy (LVH: 110,180 exams, 4.0%), and sinus tachycardia (ST: 107,212 exams, 3.9%). These findings illustrate the diverse clinical spectrum captured by the dataset, ranging from routine screenings to structural and electrical abnormalities. Less frequent but clinically relevant conditions, such as atrial fibrillation (AF: 52,513 exams, 1.9%) and complete bundle branch blocks—left bundle branch block (LBBB: 60,804 exams, 2.2%) and right bundle branch block (RBBB: 49,473 exams, 1.8%)—were also well represented. The complete distribution of all 66 classes, together with their associated labels and the training-validation split that will be used in subsequent analysis, is provided in Supplementary Table 2.

The distribution of exams across the 10 CODE diagnostic groups was also analyzed. As illustrated in Fig. 2b, normal was the most prevalent category (1,364,623 exams, 49.9%), followed by miscellaneous conditions (688,273 exams, 25.2%), chamber hypertrophy (403,756 exams, 14.8%), arrhythmia (281,992 exams, 10.3%), and intraventricular conduction disturbances (268,651 exams, 9.8%). Since normal is the only CODE group composed of a single diagnosis that is mutually exclusive with the remaining 65 diagnostic classes, we further examined the number of exams exclusively assigned to each group. Supplementary Fig. 6 presents this distribution, the 10 most frequent combinations of non-exclusive group assignments (among 251 distinct patterns observed), and the distribution of the number of CODE groups assigned per exam. This group-level analysis highlights not only the clinical diversity of the dataset but also its potential for developing and evaluating diagnostic models across a wide range of cardiovascular patterns, from benign findings to complex arrhythmogenic and structural conditions.

Additionally, we evaluated the number of diagnostic classes assigned per ECG. As illustrated in Fig. 2c, most exams were annotated with only one CODE class, with decreasing frequencies for exams containing multiple labels. This distribution reflects the fact that, while many ECGs show isolated findings, a substantial subset presents with two or more co-occurring abnormalities. This highlights the need for AI models capable of handling multilabel classification tasks.

To minimize bias due to repeated exams from the same individuals, we performed an additional analysis using only the first ECG per patient (Supplementary Table 3). The relative frequencies remained consistent.

### CODE-II-test: a curated test set for model evaluation

The CODE-II-test dataset consists of 8475 ECGs collected between 2018 and 2025 from unique patients not present in the CODE-II dataset. Each exam was annotated by multiple certified cardiologists using the 66 CODE diagnostic classes. Final labels were assigned following predefined agreement and majority-based rules: 6309 exams (74.4%) achieved complete concordance among reviewers, while 2166 exams (25.6%) were adjudicated by a label-wise majority vote, retaining all diagnostic classes receiving at least two votes (with the Normal class assigned exclusively when applicable, consistent with its mutually exclusive definition). Labeling consistency was further quantified via per-class pairwise inter-rater agreement: 50/66 (75.8%) classes achieved median Cohen’s kappa ≥0.90, and no class fell below 0.70 (Supplementary Table 16). To ensure balanced representation, additional care was taken to include cases covering rare diagnostic classes. This curated dataset was specifically designed to provide a reliable benchmark for evaluating the performance of AI models using high-quality, expert-reviewed ECGs.

Among the 8475 patients included in the CODE-II-test, 57.5% (4871) were female and 42.5% (3604) were male. The overall mean age was 55.5 years, with similar averages for female (55.3 years) and male (55.7 years) patients. Patients were stratified into two age groups: 18–59 years, comprising 58.6% of the cohort (2898 females and 2068 males), and 60 years or older, comprising 41.4% (1973 females and 1536 males). No patients under 18 years of age were included.

The distribution of the 66 CODE diagnostic classes and their aggregation into 10 diagnostic groups highlight the broad spectrum of electrocardiographic findings represented in the dataset. Normal ECGs were the most frequent (3398 exams, 40.1%), followed by abnormalities such as nonspecific ST-T changes (969 exams, 11.4%) and left atrial enlargement (912 exams, 10.7%), alongside a wide range of other clinically relevant abnormalities. A complete description and detailed characterization of this dataset, including extended tables and figures on patient- and exam-level attributes, are provided in the Supplementary Materials.

### Baseline AI model for CODE diagnostic classification

We developed a deep neural network to classify ECG exams according to the 66 CODE diagnostic classes (Fig. 8). It is built upon a convolutional residual architecture previously employed by the TNMG research group to identify clinically relevant abnormalities in rhythm and morphology. Adapted from the ResNet model originally introduced for image classification^22,23^, the architecture was modified to process one-dimensional ECG signals and has demonstrated expert-level performance in earlier studies^3^.

The model was trained on the large-scale CODE-II dataset and evaluated on the independent, curated CODE-II-test set. The datasets originate from the same telehealth network (TNMG), but are non-overlapping, containing exams collected from different patients to allow for an unbiased assessment. In addition to evaluations based on the CODE diagnostic classes, we also explored the utility of this model’s encoder when applied to external ECG datasets annotated with alternative diagnostic schemes.

### Performance of the model on the CODE-II-test

For threshold-independent metrics, we report the Area Under the Receiver Operating Characteristic Curve (AUROC) and the Area Under the Precision–Recall Curve (AUPRC, also referred to as Average Precision, AP). These metrics quantify the model’s discriminative ability by evaluating how well it separates and ranks exams according to the likelihood of each diagnostic class, and are therefore useful for benchmark characterization across different potential clinical workflows (e.g., screening, triage, or diagnostic assistance), without committing to a specific operating point. The model achieved a micro-averaged AUROC (micro-AUROC) of 0.983 and AUPRC of 0.776, and a macro-averaged AUROC (macro-AUROC) of 0.978 and AUPRC of 0.561, indicating robust discriminative ability, with consistently strong performance across common classes and greater variability among rare categories. Stratified analyses by sex, age group, state (Minas Gerais vs all other Brazilian states represented in CODE-II-test), and comorbidity burden (0 vs ≥1 of seven pre-specified clinical conditions) yielded consistently strong discrimination, with micro-AUROC ranging from 0.976 to 0.989 and micro-AUPRC from 0.727 to 0.817 across subgroups (Supplementary Table 17). Macro-averaged results were likewise stable across strata (macro-AUROC 0.970–0.983; macro-AUPRC 0.551–0.573). We additionally evaluated probability calibration on the CODE-II-test using Expected Calibration Error (ECE) and Brier score, computed directly from the raw predicted probabilities. These results are reported in the Supplementary Information (Supplementary Tables 18 and 19; Supplementary Fig. 29) as a characterization of model reliability in this benchmark setting, without any post-hoc recalibration.

**Fig. 3:**
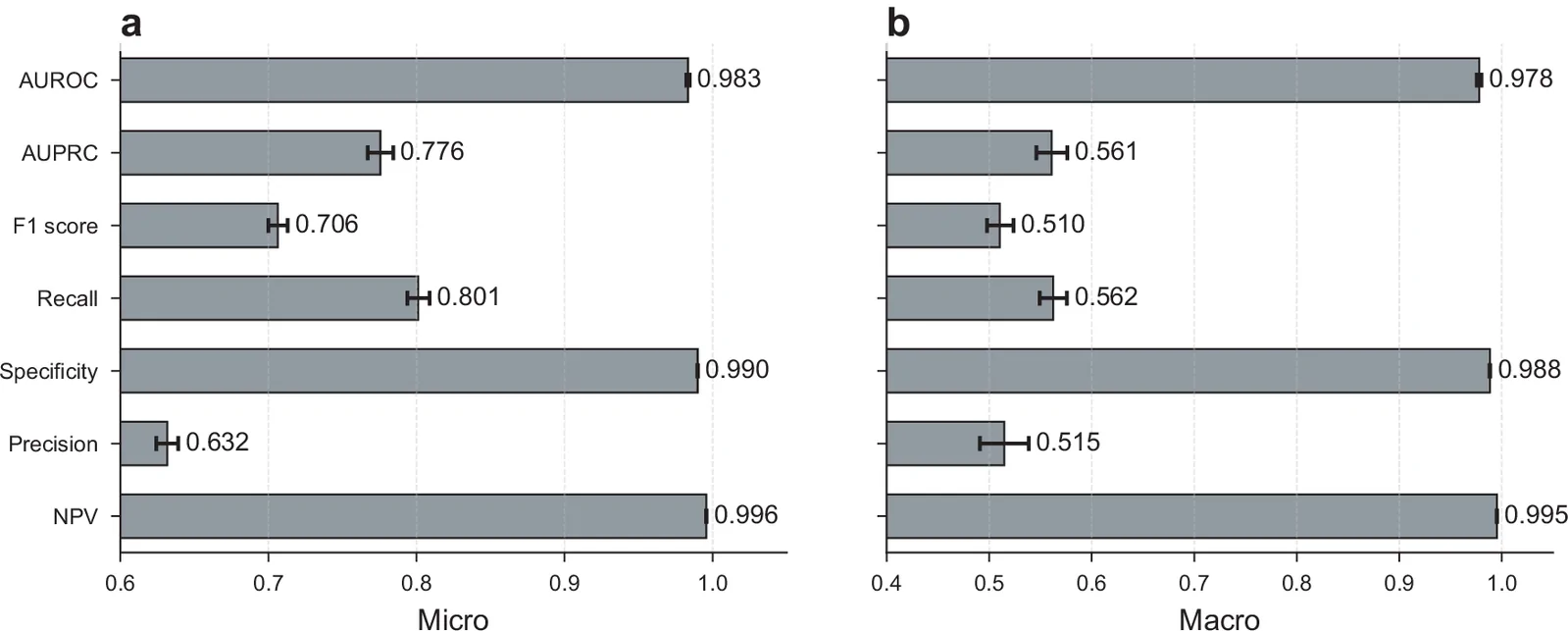
Global performance of the model on the CODE-II-test dataset. Panels show **a** micro- and **b** macro-averaged results for AUROC, AUPRC, F1-score, Recall, Specificity, Precision, and NPV. Threshold-dependent metrics were computed after applying class-specific thresholds selected to maximize the F1-score. Bars represent the mean metric values, with numerical values shown to the right of each bar, and horizontal lines denoting 95% confidence intervals estimated from 1000 bootstrap resamples.

For threshold-dependent metrics, operating points must be defined according to the intended use case and the relative cost of false positives and false negatives (Discussion). As a standardized baseline for benchmarking, when applying class-specific thresholds that maximize the F1-score, the model reached a micro-F1 of 0.706, precision of 0.632, recall (sensitivity) of 0.801, specificity of 0.990, and negative predictive value (NPV) of 0.996, reflecting solid global performance with a tendency toward higher sensitivity at the cost of lower precision. These thresholds were selected on validation and applied unchanged to the test set, ensuring a fair evaluation while making threshold-dependent metrics sensitive to shifts in prevalence and calibration between datasets. Macro-averaged scores were lower, with a macro-F1 of 0.510, precision of 0.515, recall of 0.562, specificity of 0.988, and NPV of 0.995, highlighting the greater difficulty of detecting rare conditions when all classes are equally weighted. These metrics are summarized in Fig. 3, where horizontal lines represent 95% confidence intervals computed from 1000 bootstrap resamples. Overall, the model performs robustly across the diagnostic spectrum, although results for rare classes remain more variable and should be interpreted with caution due to class imbalance.

**Fig. 4:**
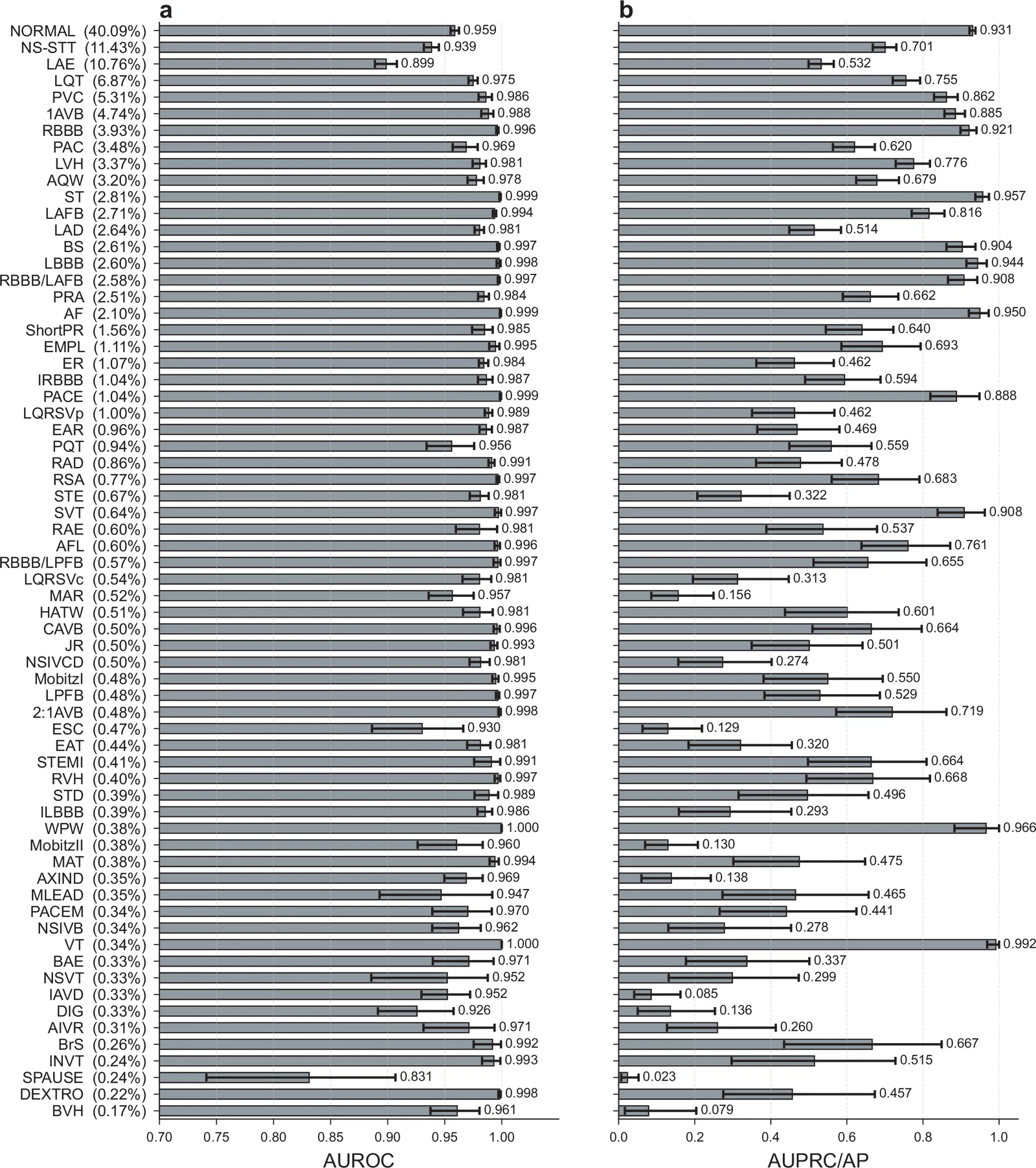
Per-class threshold-independent performance of the model on the CODE-II-test. Panels show **a** AUROC and **b** AUPRC (average precision) with 95% confidence intervals computed from 1000 bootstrap resamples. Bars indicate the mean metric value for each diagnostic class, with the numerical value shown to the right of each bar. The horizontal lines represent the corresponding confidence intervals. Diagnostic classes are ordered by their prevalence in the test set (shown in parentheses), where prevalence divided by 100 corresponds to the expected AUPRC of a random classifier.

Per-class analysis revealed strong discriminative ability across both common and rare diagnoses (Fig. 4). For example, the ECG within normal limits for age and sex (NORMAL class), representing approximately 40.1% of the test set, achieved an AUPRC of 0.931—more than twice its prevalence (0.401)—underscoring the model’s reliability in identifying exams without abnormalities. The model also performed well for moderately prevalent but clinically important abnormalities: LBBB (2.6%) reached an AUPRC of 0.944, and atrial fibrillation (AF, 2.1%) achieved an AUPRC of 0.950. Even in rare but critical conditions such as ST-elevation myocardial infarction (0.4%), the model obtained an AUPRC of 0.664, substantially above prevalence, demonstrating its ability to prioritize time-sensitive cases despite limited representation. By contrast, performance was more modest in extremely rare classes; for instance, isorhythmic atrioventricular dissociation (IAVD, 0.3%) achieved an AUPRC of only 0.085, illustrating ongoing challenges in detecting sparsely represented conditions. The horizontal lines in Fig. 4 represent 95% confidence intervals computed from 1000 bootstrap resamples. Full per-class results, including AUPRC, AUROC, prevalence, and threshold-dependent metrics, are provided in Supplementary Table 9.

All primary threshold-dependent results reported here use the predefined class-specific F1-max thresholding protocol (Methods), with thresholds selected on the CODE-II validation set and applied unchanged to CODE-II-test. As a sensitivity analysis (Supplementary Information), we compared F1-max with an alternative threshold-selection rule based on Youden’s J statistic. Under Youden’s J, micro-averaged precision decreased to 0.228 and recall increased to 0.946, yielding a lower F1 of 0.368 (macro precision 0.182, recall 0.945, F1 0.274). These results are reported alongside those obtained with F1-max thresholds in Supplementary Table 10 (micro/macro). At the class level, we compare just 3 of 66 diagnostic classes—multifocal atrial tachycardia, IAVD, and digitalis effect (DIG)—for which F1-max thresholds selected on validation produced degenerate values on Test (precision, recall, and F1 equal to zero) and show that Youden’s J yielded non-zero values. In these cases, the Youden-selected thresholds were several orders of magnitude smaller than the validation F1-max thresholds, enabling positive predictions and non-zero precision/recall/F1 (Supplementary Table 11). Taken together, these comparisons show that thresholds chosen on validation can materially alter threshold-dependent performance estimates when applied to a different test distribution. Full per-class metrics under the primary protocol are provided in Supplementary Table 9; comparative results for threshold choice appear in Supplementary Tables 10 and 11.

Regarding cardiologist performance on the CODE-II-test, results are uniformly high under a fair-scope protocol aligned with the agreement/majority labeling rules used to define the ground-truth labels (Supplementary Table 8). Because the reference labels are constructed by adjudication from the same pool of certified cardiologists, these metrics should be interpreted as agreement with the adjudicated benchmark (inter-reader consistency under the CODE framework), rather than clinical diagnostic accuracy against an independent gold standard. Among high-volume reviewers (≥100 exams; *n* = 20 cardiologists), the mean micro-F1 was 0.966 (mean micro-recall 0.978; mean micro-precision 0.956), and the mean macro-F1 was 0.944, with macro precision and recall likewise high. Performance varies with workload and breadth of classes encountered: the highest-volume reviewer (8368 exams; 65 classes) achieved micro-F1 0.869 (precision 0.821; recall 0.924) and macro-F1 0.844 (precision 0.805; recall 0.915), lower than the high-volume mean and reflecting more realistic conditions under greater workload and broader diagnostic coverage. The model, in turn, maintains strong ranking ability (AUROC/AUPRC), while threshold-dependent aggregate metrics are sensitive to thresholds chosen on validation and applied unchanged to the CODE-II-test set with different class prevalence and score calibration—under F1-max thresholds, micro-F1 0.706 (precision 0.632; recall 0.801) and macro-F1 0.512 (precision 0.517; recall 0.562) (Supplementary Tables 9–11).

### Scaling laws in CODE-II: How training dataset size influences model performance

**Fig. 5:**
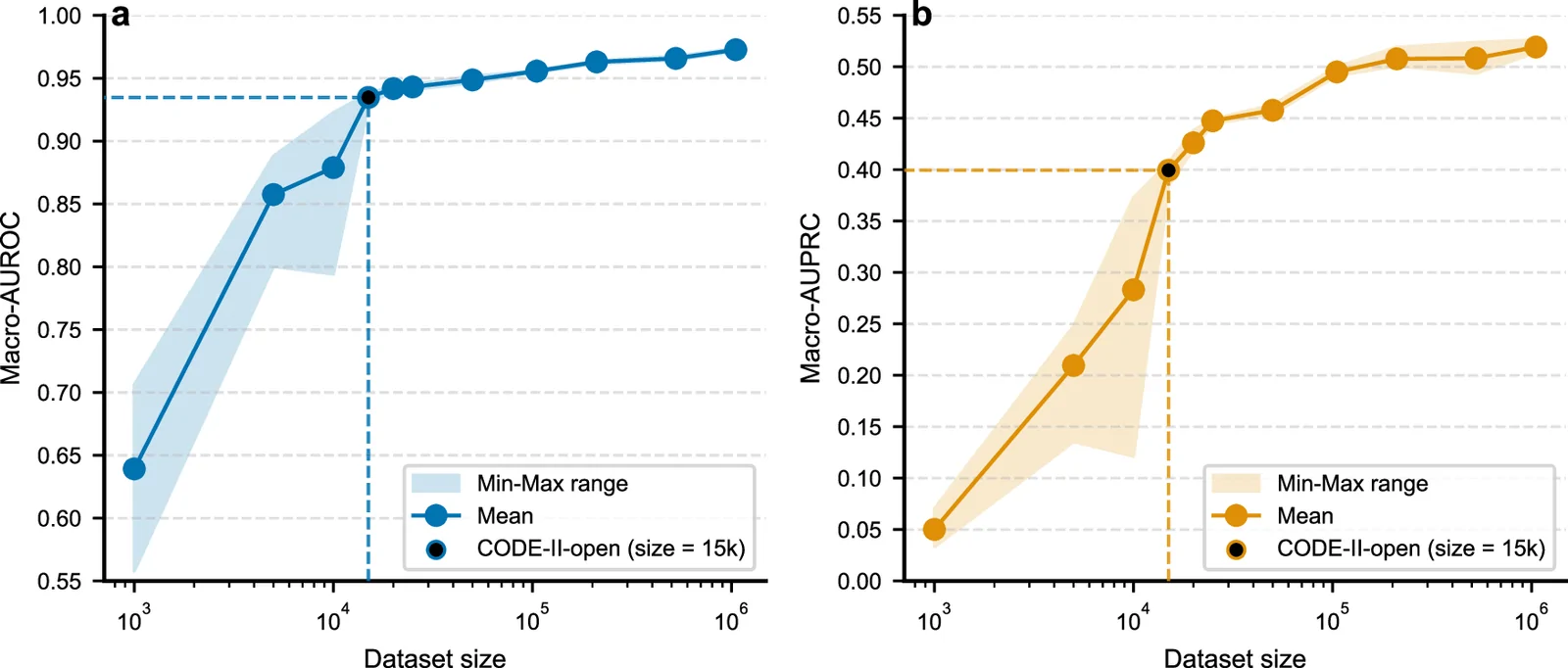
Scaling law analysis of model performance. **a** Macro-AUROC and **b** macro-AUPRC as a function of dataset size on the fixed CODE-II-test. The curves represent the mean performance across three independent runs, with shaded areas indicating the range between the minimum and maximum values.

The effect of dataset size on model performance, a behavior consistent with previously observed scaling laws in deep learning^24^, is shown in Fig. 5 for both macro-AUROC and macro-AUPRC/AP on the fixed CODE-II-test. Dataset sizes ranged from small subsets of 1000 patients up to 1,050,000 patients, corresponding to approximately 50% of the patient-level dataset, with the CODE-II-open also included in the analysis. As expected, larger datasets for model development consistently led to improved performance, although gains diminished as dataset size increased. Each curve represents the mean performance across three independent runs for a given dataset size, while the shaded area indicates the range between the minimum and maximum values observed across these runs.

As shown in Fig. 5, model performance (macro-AUROC and macro-AUPRC) improved sharply with increasing dataset size up to around 15,000 patients, after which the rate of improvement became progressively smaller. This transition coincides with the CODE-II-open, confirming it as the inflection point of the scaling curve. Variability across runs was higher at the smallest dataset sizes and narrowed as the dataset grew, supporting the robustness of the observed trend beyond the inflection region. These findings provide empirical evidence for the importance of dataset scale in ECG classification and quantify the performance gap between publicly available and full-scale training resources. Importantly, they also demonstrate that most of the attainable performance can be achieved with substantially fewer data than the total available.

In particular, as shown in Fig. 5, training on CODE-II-open (15,000 patients) achieves macro-AUROC values close to those obtained with substantially larger training subsets, while macro-AUPRC continues to improve at larger scales due to improved representation of rare diagnostic classes. The absolute performance achieved when training on the full CODE-II dataset, reported in the section “Performance of the model on the CODE-II-test,” approaches the upper bound of this scaling behavior. Despite these expected absolute differences, the CODE-II-open subset reproduces the main performance trends and relative model behavior observed across the full scaling curve, supporting its use as a public and reproducible benchmark.

Together with the analysis on CODE-II-open, these results highlight the practical trade-off between dataset size and marginal performance gains, offering guidance for future model development and data-sharing initiatives in ECG research.

### Comparative evaluation on external ECG benchmarks

To further assess the generalizability of the representations learned by our model, we fine-tuned the model pre-trained on CODE-II on two external ECG classification benchmark datasets: PTB-XL^15^, comprising 21,837 clinical 12-lead ECG recordings, and CPSC 2018^25^, containing 9831 12-lead recordings. For PTB-XL, experiments were conducted under two training settings: (i) full-data training, using 100% of the training data, and (ii) few-shot learning, using only 5% and 10% of the training data, in order to evaluate the ability of the pre-trained model to adapt and generalize from limited labeled data. This design simulates real-world scenarios with limited labeled data and enables a comprehensive assessment of model transferability.

We benchmark our model against a set of supervised models trained from scratch and publicly available pre-trained ECG models. Full model names, citations, and implementation details are provided in the “Methods” section. To evaluate the impact of pre-training, we also compare each pre-trained model with a version trained from scratch on the external dataset using randomly initialized weights.

**Fig. 6:**
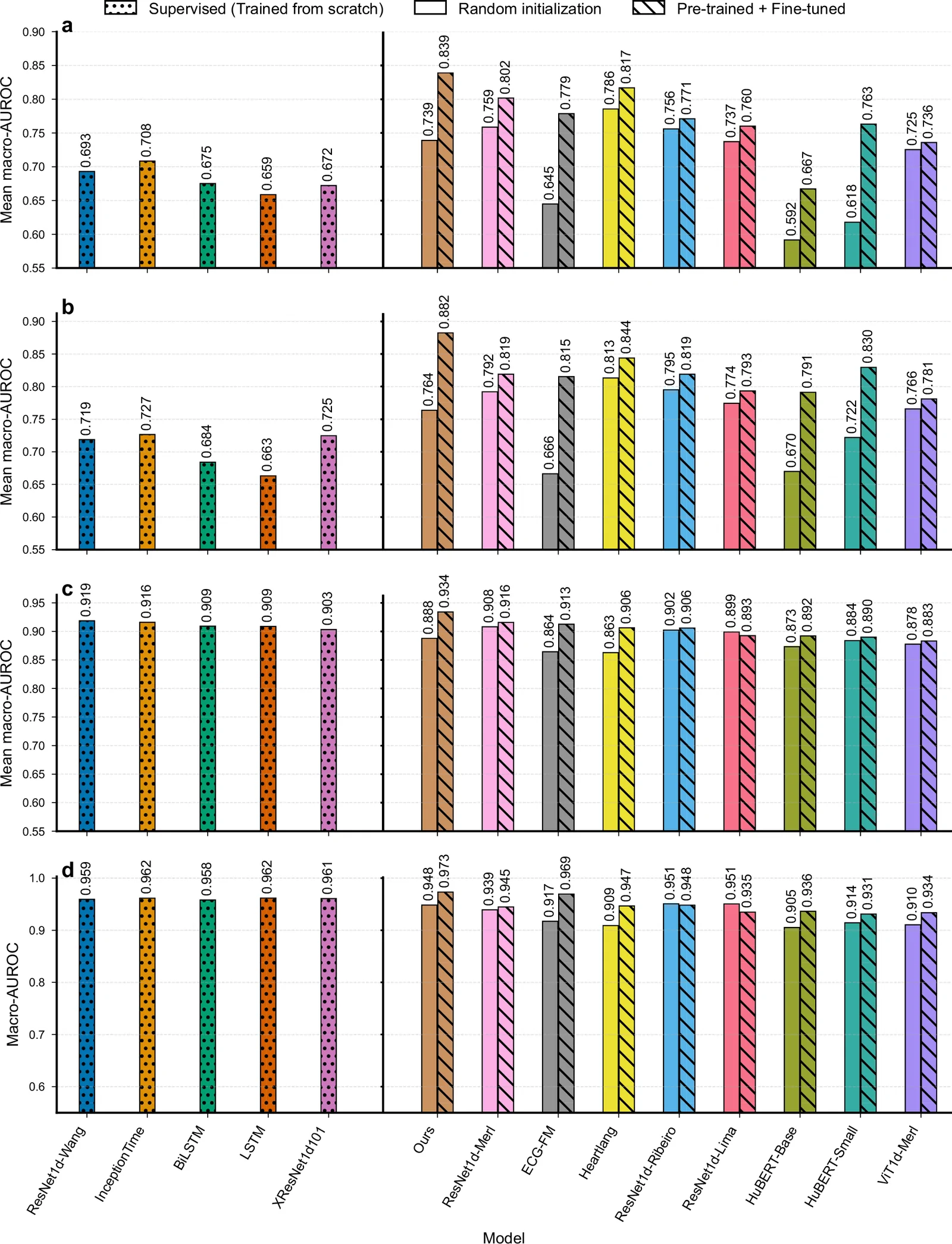
Generalization performance of supervised and pre-trained models across external ECG benchmarks. Each panel summarizes model performance across training regimes ranging from few-shot to full-data settings: **a**–**c** PTB-XL mean macro-AUROC, obtained by averaging each model’s macro-AUROC across the five diagnostic taxonomies (diagnostic, subclass, superclass, format, and rhythm) when trained with 5%, 10%, and 100% of the data; and **d** CPSC 2018 macro-AUROC. Colors denote distinct model architectures, while bar patterning indicates the training regime: dotted (supervised models trained from scratch), diagonal-hatched (pre-trained models fine-tuned on the target dataset), and solid (same architecture trained from scratch, with random initialization, used to assess the effect of pre-training). Across most architectures, fine-tuned pre-trained models achieve higher performance than their randomly initialized counterparts.

In Supplementary Data 6, we report the average macro-AUROC across three independent runs for all evaluated models, with the performance range (maximum–minimum) indicated in parentheses. The number of model parameters is also reported in Supplementary Data 6. As shown in Supplementary Table 12, our model consistently achieved the highest average macro-AUROC across all 5 PTB-XL diagnostic categories and the CPSC 2018 dataset (average macro-AUROC of 0.9405) under full-data training, demonstrating strong robustness as reflected by an average range of 0.003. It outperformed both the supervised and pre-trained baselines. Under the more challenging few-shot learning setting, our model ranked first or second in 8 out of 10 scenarios. Notably, although supervised models perform competitively under full-data training, their performance declined significantly under few-shot conditions, highlighting the superior transferability of pre-trained models. Overall, our model was the only model that achieved an average macro-AUROC above 0.9 across all datasets (0.9071), surpassing the second-best model, Heartlang, by 3.27%. Compared to the randomly initialized counterpart, our pre-trained model showed a substantial improvement of 8.68%, thereby demonstrating the effectiveness of pre-training on the CODE-II dataset (see Supplementary Data 6). Importantly, CODE-II is only 30.01% the size of HuBERT’s pre-training datasets (9.1 million ECGs), and our model achieved superior performance with only 7.85% of ECG-FM, 18.27% of Heartlang, and 23.62% of the parameters of HuBERT-Small. Figure 6 summarizes model performance from the few-shot to full-data regimes, reporting the mean macro-AUROC across all 5 PTB-XL diagnostic categories for 5%, 10%, and 100% of the training data (Fig. 6a–c) and the results for the CPSC 2018 dataset (Fig. 6d).

### CODE-II-open: benchmark dataset and a brief model comparison

The CODE-II-open dataset, which will be publicly released as part of this work, comprises 15,000 unique patients and constitutes a carefully curated subset of the CODE-II dataset. Its main characteristics are similar to those of the full dataset, with a detailed description provided in the Supplementary Materials. Despite being smaller than the full CODE-II dataset, CODE-II-open is a substantial public benchmark, comparable to existing open datasets, including PTB-XL (21,837 ECGs from 18,885 patients, Germany) and CPSC 2018 (9831 ECGs from 9458 patients, China). Crucially, CODE-II-open inherits the rigorous data-curation procedures and the clinically meaningful CODE diagnostic classes established by certified cardiologists at TNMG, which have been applied and tested in routine telecardiology practice for over a decade.

**Fig. 7:**
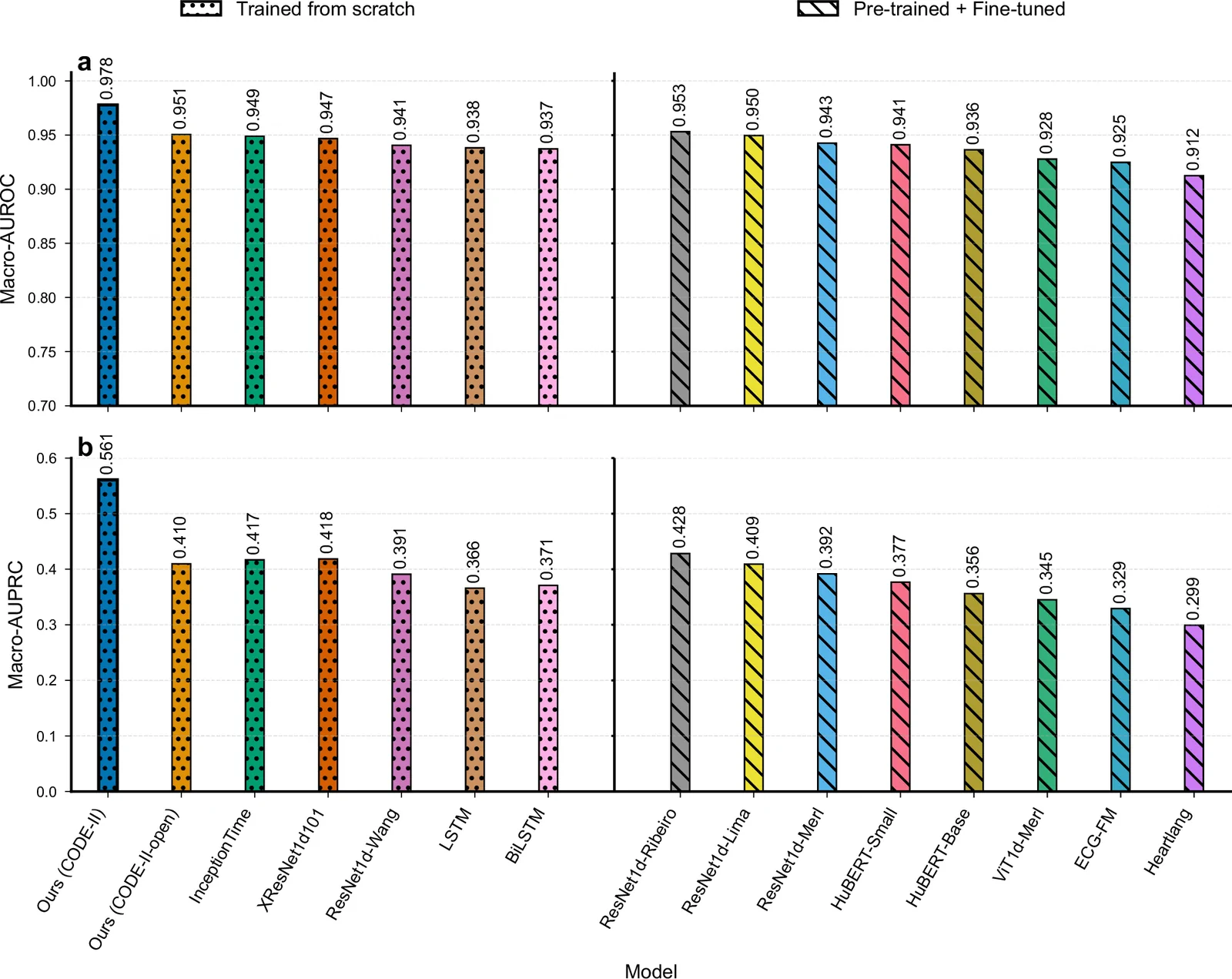
Models evaluated on CODE-II-open and CODE-II-test. Panels show **a** macro-AUROC and **b** macro-AUPRC for all models trained on the CODE-II-open dataset and evaluated on the CODE-II-test set. Models were either trained from scratch or fine-tuned from pre-trained versions. Colors indicate distinct model architectures, while bar patterning denotes the training regime: dotted for models trained from scratch and diagonal-hatched for pre-trained models fine-tuned on CODE-II-open. For reference, the baseline model trained from scratch on the full CODE-II dataset is also included.

Using the same evaluation pipeline and external models described in the previous subsection, we conducted a concise analysis to assess the representativeness of the CODE-II-open dataset as a benchmark. Supplementary Data 7 reports macro-AUROC and macro-AUPRC for three independent runs of all evaluated models, along with the performance range (maximum–minimum) in parentheses. The mean values across runs are summarized in Supplementary Table 15 and illustrated in Fig. 7. Models were trained either from scratch or fine-tuned from pre-trained versions, and the best-performing model based on validation macro-AUROC on CODE-II-open was evaluated on CODE-II-test. Our baseline architecture trained from scratch on CODE-II-open achieved a macro-AUROC of 0.951 and a macro-AUPRC of 0.410, whereas the same architecture pre-trained on the full CODE-II dataset reached 0.978 and 0.561, respectively. Despite being trained on only approximately 0.72% of the available data, the CODE-II-open model matched or outperformed several well-established ECG models under their best-performing regimes, indicating that the dataset is sufficiently rich and diverse to support the development of competitive ECG classifiers. The performance gap observed between our model trained on the full CODE-II dataset and the same architecture trained on CODE-II-open follows the scaling-law trends presented in Fig. 5.

Together, the scaling-law analysis including CODE-II-open (Fig. 5) and the benchmark experiments restricted to CODE-II-open (Fig. 7; Supplementary Table 15; Supplementary Data 7) show that the key benchmark conclusions of this work, including the main performance trends and relative model behavior, can be reproduced using only the public subset. In particular, training on CODE-II-open yields performance that approaches our full-dataset model in macro-AUROC, whereas macro-AUPRC continues to benefit from larger-scale training due to improved representation of rare diagnostic classes.

These results position the CODE-II-open as both a realistic and valuable public benchmark for ECG classification research. Beyond enabling transparent model evaluation, it provides an accessible foundation for future studies on model pre-training, fine-tuning, and cross-dataset generalization.

## Discussion

Our work provides a comprehensive resource for advancing AI-based ECG analysis. A key strength is the CODE-II dataset, characterized by high-quality annotations derived from standardized diagnostic criteria and reviewed by cardiologists with extensive expertise in ECG interpretation. The introduction of 66 diagnostic classes—the CODE diagnostic classes—developed in collaboration with clinical experts, captures a broad spectrum of ECG findings while providing a clinically meaningful and interpretable label space that aligns with cardiological reasoning and remains suitable for model development. The open release of the CODE-II-open dataset, a representative subset, and the rigorously curated CODE-II-test, reviewed by multiple cardiologists, provides the research community with robust benchmarks for developing and evaluating automated ECG classification models within this framework. Together, these resources combine diagnostic breadth, clinical heterogeneity, and expert curation to establish a new reference standard for automated ECG analysis. The performance on CODE-II-test provides a rigorous benchmark characterization of model behavior in an expert-adjudicated setting and can guide the development and evaluation of algorithms intended for telecardiology workflows, without implying deployment-ready optimization.

Building on these foundations, CODE-II represents a substantial advance in scope and design compared with the original CODE dataset (CODE-I)^3^. CODE-I was a landmark study showing that deep neural networks could outperform clinicians on 12-lead ECG interpretation, but it was restricted to six rhythm and conduction classes, such as atrial fibrillation, first-degree atrioventricular block, sinus bradycardia and tachycardia, and bundle branch blocks. CODE-II overcomes these limitations by expanding the label space from 6 to 66 classes, capturing a broader range of ECG abnormalities not represented in CODE-I. It also introduces new subsets: while CODE-I provided the CODE-15% subset and a test set of 827 ECGs, CODE-II adds two purpose-built resources—the CODE-II-open, a public subset of 15,000 unique patients, and the CODE-II-test, a non-overlapping collection of 8475 unique patients designed for blind evaluation under standardized criteria. By pre-defining clinically stratified training and validation splits in CODE-II-open, the dataset mitigates sampling bias and enables fairer algorithmic comparisons, analogous to PTB-XL^15^. Initial evaluations suggest that models trained on CODE-II achieve high performance across many more diagnostic categories than what was possible with CODE-I, highlighting the advantages of a richer and more informative label space.

A key demonstration of the value of the CODE-II dataset lies in its ability to support models that generalize effectively to external datasets. When fine-tuned on established ECG benchmarks such as PTB-XL^15^ and CPSC 2018^25^, our proposed model, pre-trained on CODE-II, consistently outperformed both supervised models trained from scratch and other publicly available pre-trained ECG models. This advantage was evident not only under full-data training but also in few-shot settings, where CODE-II pre-training enabled strong performance even when only a small fraction of the available training data was used. Such robustness highlights the transferability of the representations learned from CODE-II, reflecting the breadth and clinical fidelity of its diagnostic classes. Importantly, our results demonstrate that models pre-trained on CODE-II consistently outperform alternatives—even those with substantially more parameters or trained on larger datasets such as HuBERT^26^, ECG-FM^27^, or Heartlang^28^. These improvements can be attributed primarily to the quality of the CODE-II data and the breadth of its 66 diagnostic classes rather than to architectural or training innovations, as underscored by the limited gains observed when training comparable models on the six classes of CODE-I. This underscores the combined value of a well-designed model architecture and a high-quality, clinically curated dataset, highlighting that label quality and a clinically meaningful diagnostic system may prove more important than dataset size alone in achieving robust and generalizable AI tools for ECG clinical analysis.

CODE-II adds value beyond existing open ECG datasets by combining large-scale, real-world telecardiology data with clinically standardized, expert-defined annotations. Each exam is labeled by certified cardiologists within the TNMG tele-ECG workflow using the 66 CODE diagnostic classes, a reporting system aligned with international (AHA/ACC) and Brazilian ECG reporting guidelines, and refined through routine clinical use with ongoing quality audits. The TNMG tele-ECG platform integrates automated ECG measurements with tools for expert review and correction, and includes a measurement-aware decision-support constraint that restricts label selection to diagnoses compatible with the original or corrected measurements, strengthening annotation consistency at scale. This yields a medically interpretable and consistent multilabel space spanning a broad spectrum of ECG abnormalities, including many low-prevalence findings that are typically underrepresented in smaller public datasets. In contrast, widely used public benchmarks such as PTB-XL and CPSC 2018 provide valuable annotations but differ in dataset size and/or label taxonomy; moreover, PTB-XL diagnostic statements may originate, for a subset of records, from automated device interpretation as part of the clinical reporting pipeline. Large-scale resources such as the Harvard–Emory ECG database explicitly report that their annotations are generated by commercial interpretation software (Marquette 12SL, GE Healthcare), which differs from cardiologist-assigned diagnoses under a standardized, clinically governed diagnostic framework. These distinctions affect annotation provenance and interpretability in clinically meaningful label spaces, and are consistent with our empirical findings that CODE-II pre-training supports strong transfer to external open benchmarks (PTB-XL and CPSC 2018) under both full-data and few-shot regimes.

There is a heterogeneity in the meaning of an ECG diagnosis, or diagnostic class as defined in this study, since it may reflect a direct measurement of the ECG intervals (for example, QT prolongation or a first-degree AV block), a disease diagnosed primarily through ECG, like atrial fibrillation or complete AV block, or a disease or condition that requires confirmation by non-electrocardiographic means, like an acute myocardial infarction, pericarditis or hyperkalemia^29^. This blending of signal patterns and clinical diagnoses can hinder both interpretability and generalizability, especially in the context of machine learning. The CODE diagnostic classes address this limitation by providing a clearer separation: they are primarily defined based on recognizable electrocardiographic patterns, leaving the recognition of a specific medical condition to the clinical reasoning, taking into account age, sex, clinical symptoms, and comorbidities. This structure preserves the richness of expert interpretation while offering a more consistent and algorithm-friendly label space. Notably, the CODE diagnostic classes have been tested and refined over more than a decade of use in a large-scale telehealth service, ensuring their practicality and robustness in real-world settings.

While machine learning is enabling novel applications of the ECG—such as predicting patient prognosis or detecting conditions traditionally beyond the reach of standard electrocardiography—it is important to emphasize that these advances build upon, rather than diminish, the value of conventional ECG interpretation. Traditional diagnostic frameworks encapsulate nearly a century of accumulated clinical knowledge, with human experts having defined categories of abnormalities such as atrial fibrillation, atrioventricular block, and ischemic changes that remain central to both clinical reasoning and machine learning. Training AI models on these well-established electrocardiographic categories allows algorithms to inherit a wealth of prior knowledge that guides pattern recognition and ensures interpretability. Indeed, even the most recent ECG foundation models rely on classic diagnostic labels curated by experts^30^. For example, a recent *Nature Communications* study defined 60 arrhythmia and ECG finding classes for an ECG model^31^, while the ECGFounder model was trained on 150 categories from the Harvard–Emory ECG database, which reflect established ECG diagnostic standards^32^. These examples highlight that cutting-edge AI initiatives extend rather than replace the traditional ECG interpretive framework. The CODE-II diagnostic classes were developed in this same spirit, embedding accumulated clinical wisdom into a structure that is both meaningful for physicians and suitable for model development using AI.

Beyond methodological advances, the implications of CODE-II have clear relevance to telecardiology workflows and potential clinical decision support. By enabling the development of AI models that combine diagnostic accuracy with operational efficiency, the dataset opens new possibilities for optimizing workflows in large-scale telehealth services. Such models can facilitate scheduling systems that prioritize urgent cases, reduce the likelihood of diagnostic errors, and allow physicians to respond more rapidly to straightforward exams—for example, by automatically identifying normal tracings and directing specialist attention to those requiring further evaluation. They can also serve as valuable decision-support tools for non-specialists working in resource-limited settings, providing a reliable second opinion and enabling earlier recognition of critical conditions. However, effectively deploying these tools requires going beyond conventional performance metrics. In particular, AUROC and AUPRC provide threshold-independent characterization of discrimination, but clinical utility depends on use-case–specific operating points that reflect the relative cost of false positives and false negatives. For instance, when the focus is on classifying abnormalities, a false positive—when the model erroneously labels a normal ECG as abnormal—may generate unnecessary alarms, additional referrals, or redundant testing, potentially overwhelming services with benign cases. Conversely, a false negative—when a true abnormality such as a serious arrhythmia or ischemic change goes undetected—is even more concerning, as it can delay urgent treatment with potentially severe consequences. Recent studies illustrate this trade-off, showing that AI models can substantially reduce false negatives compared to human readers while modestly increasing false positives in ambulatory ECG monitoring^33^. To ensure that AI models trained on CODE-II genuinely benefit telehealth workflows, it is essential to evaluate not only global performance metrics but also the clinical and operational impact of errors at both macro and micro levels. In designing and evaluating AI for telecardiology, sensitivity (recall) and precision (positive predictive value) should be balanced according to clinically motivated thresholding strategies tailored to specific clinical applications. For example, we previously reported preliminary results for normal-ECG detection^34^, highlighting that a high-precision threshold is particularly relevant for minimizing missed abnormalities and avoiding delays in clinical intervention. Conversely, a high-recall threshold is more appropriate for urgent abnormalities such as ST-elevation patterns, where capturing all potential cases—even at the expense of precision—is essential to avoid missing life-threatening events. To complement discrimination metrics, we also report calibration on CODE-II-test using ECE and Brier score computed directly from the raw predicted probabilities (without post-hoc recalibration; Supplementary Tables 18 and 19 and Supplementary Fig. 29), which supports interpretation of probabilistic outputs in this benchmark setting. In future work, we will formalize these operating modes and quantify their impact on workflow efficiency; deployment-oriented optimization will require workflow-specific evaluation, potentially including decision-analytic analyses, under distribution shift.

At the class level, however, persistent challenges remain for a subset of low-prevalence diagnoses. Despite the scale of the full CODE-II dataset, several diagnostic classes remain extremely rare in the expert-adjudicated CODE-II-test benchmark, and some of these exhibit low AUPRC values. This pattern can arise from multiple, non-mutually exclusive factors. First, when prevalence is very low, AUPRC is inherently sensitive to a small number of false positives and to uncertainty in the estimated precision–recall curve, typically yielding wider bootstrap confidence intervals even when ranking ability (AUROC) remains high (Fig. 4; Supplementary Table 9). Second, consistent with the heterogeneity in what an “ECG diagnosis” represents, some labels are intrinsically harder to infer from the waveform alone, as they may involve subtle or borderline morphologies, transient manifestations, overlap with related classes, or a clinical context that extends beyond the electrocardiographic trace. Third, for low-prevalence classes, limited cardiologist coverage and inter-rater variability can contribute to effective label noise: in CODE-II-test, we report per-class cardiologist coverage and pairwise agreement (including Cohen’s kappa when defined), and show that both coverage and the fraction of cardiologist pairs with defined versus undefined kappa differ by class. For the lowest-prevalence diagnoses, Cohen’s kappa is often undefined for a non-trivial fraction of cardiologist pairs because shared-exam annotations can be degenerate (all-negative or all-positive), which is expected when shared positive counts are very small (Supplementary Table 16). Finally, threshold-dependent metrics can become degenerate for a small subset of classes under fixed validation-selected operating points, reflecting distribution shift in prevalence and calibration between validation and test rather than absence of signal (Supplementary Table 11). Together, these observations suggest that persistent low AUPRC in a subset of rare classes is not necessarily explained by a single cause (e.g., data volume alone), but may reflect a combination of scarcity, intrinsic ambiguity, and inter-reader variability. More broadly, learning from long-tailed multilabel data is an active area of research, and rare-class performance may be improved with imbalance-aware strategies (e.g., re-sampling or loss reweighting), as illustrated in recent ECG foundation-model work^32^. However, a detailed exploration of these approaches is beyond the scope of this paper, and we intentionally use standard binary cross-entropy to minimize inductive bias and preserve comparability across all experiments. Addressing these limitations is an important direction for future work, including targeted enrichment of rare cases, focused re-auditing and refinement of class definitions for ambiguous labels, and evaluation strategies that incorporate clinically meaningful grouping or hierarchical structure when appropriate.

Beyond these per-class challenges, certain limitations should be acknowledged. CODE-II is derived from a single national telehealth system, and although it encompasses millions of exams, its population characteristics may not fully capture the diversity of global clinical settings. Moreover, the CODE diagnostic classes were intentionally designed to reflect recognizable electrocardiographic patterns that are well-suited for machine learning but do not replace the broader clinical reasoning that integrates symptoms, comorbidities, and complementary examinations. The dataset also reflects the inherent variability of real-world ECG acquisition, including artifacts (e.g., noise from movement or electrode contact) and device heterogeneity, which, while increasing ecological validity, can introduce additional challenges for AI model development. Recognizing these limitations does not diminish the value of CODE-II; rather, it highlights key directions for future research.

As demonstrated in our external evaluations on datasets from Germany (PTB-XL) and China (CPSC 2018), models pre-trained on CODE-II showed good cross-dataset performance on independent cohorts with different label taxonomies, underscoring the transferability of the learned representations under cross-dataset domain shift. These external datasets also differ from CODE-II in healthcare settings and acquisition contexts (e.g., routine clinical ECG workflows in Germany and China versus a large-scale national telecardiology service in Brazil), providing additional evidence of model robustness across distinct care environments. At the same time, external validation remains necessarily incomplete: PTB-XL and CPSC 2018 cannot exhaustively represent variability across all healthcare systems, demographic subgroups, and ECG acquisition devices and recording protocols. Moreover, key factors required for deeper stratified analyses are not consistently available as standardized metadata in the datasets used here. For example, ethnicity is not systematically recorded in CODE-II and is also not provided in a uniform, structured way across the external benchmarks used in this study, precluding robust ethnicity-stratified evaluation. Similarly, detailed vendor/protocol information (e.g., device model) is not consistently available to support comprehensive stratification. These limitations motivate broader multi-center evaluations and richer stratified analyses as such metadata becomes available in a standardized manner. Future multinational collaborations will be important to extend validation across additional healthcare systems, populations, and acquisition environments and to better characterize robustness across diverse devices and protocols. Beyond cross-dataset validation, another promising avenue is the integration of ECG with multimodal clinical data, extending the utility of CODE-II beyond diagnostic classification toward prognostic modeling and risk stratification—similar to prior efforts in CODE-I, where a subset of records was linked to mortality outcomes^3^. Finally, further investigation into threshold selection and calibration—including probability scaling and prevalence-aware adjustment—is an important next step, given the observed shifts in class prevalence and score calibration between the validation and test sets, which make threshold-dependent metrics particularly sensitive.

Comprehensive dataset descriptions—including patient demographics, comorbidities, symptoms, and ECG interval measurements stratified by sex and age—are provided in the Supplementary Materials to facilitate broader scientific use. A brief analysis of these electrocardiographic measurements revealed physiologically consistent sex- and age-related differences, in line with prior population-based studies and known physiological patterns^35–38^. Such consistency underscores the clinical validity of the CODE-II dataset and supports its potential for secondary research beyond diagnostic modeling, including studies on electrophysiological variability, population health, and disease risk profiling. Taken together, the rigorously curated CODE-II dataset, its 66-class diagnostic system refined through more than a decade of telehealth practice, and the publicly available CODE-II-open and CODE-II-test subsets establish a foundation for global collaboration and continuous progress in AI-driven cardiology. In the era of emerging *ECG foundation models* trained on increasingly large datasets, CODE-II offers a curated, clinically meaningful, and expertly annotated benchmark that can strengthen this growing research area by providing robust, interpretable diagnostic labels grounded in real-world practice.

## Methods

### Tele-electrocardiogram system

The TNMG tele-ECG system was developed by the network’s in-house team. Instead of transmitting images of the ECG tracings, the system captures electrocardiographic signals directly from digital electrocardiographs of different models, enabling efficient management and allowing further processing of the acquired ECG data^7^.

A primary care professional performs the ECGs remotely using digital electrocardiograms. This professional, typically a nursing technician, applies a standardized clinical questionnaire that includes the type of exam (urgent, elective or preferential), date of birth, sex (recorded as male or female, corresponding to biological sex), clinical indication, symptoms, current medications, and self-reported comorbidities: smoking, hypertension, diabetes, dyslipidemia, Chagas disease, previous myocardial infarction, and chronic obstructive pulmonary disease. Electrocardiography machines record standard 12-lead ECG signals, typically sampled at rates of 300 Hz, 500 Hz, 600 Hz, or 1000 Hz. Each exam may consist of multiple tracings, in general, 2–4, each lasting 7–12 s, captured successively.

An in-house software platform was developed and is integrated with electrocardiography machines to capture ECG tracings, upload them with the patient’s clinical history, and transmit them via the Internet to the Telehealth Center at the Hospital das Clínicas of UFMG. All received ECGs are converted to a custom format that meets the current needs of healthcare operations and academic research. These exams are preprocessed for automatic measurement extraction and then organized into a priority queue based on the user-suggested priority, time of receipt, and ECG data. The web platform for ECG reports displays the ECG tracings, clinical data, and automatic measurements of the ECG waves and intervals provided by the University of Glasgow ECG system and integrated into our software. The platform has several tools for magnifying, filtering, and correcting the automatic measurements. Urgent cases are indicated, with abnormal measurements displayed in red, and borderline values highlighted in orange. All ECGs are analyzed by a certified cardiologist, who selects one or more ECG CODE classes. A decision support system was developed to allow the selection of only those diagnosis classes compatible with the original or corrected measurements^39^. When the cardiologist completes the report, a PDF file becomes available for download at the original remote point, including the identification, clinical data, ECG tracings, and the full report. A monthly audit by senior cardiologists of a sample of randomly selected ECGs is conducted to ensure the quality and consistency of the cardiological expertise.

### CODE diagnostic classes

**Fig. 8:**
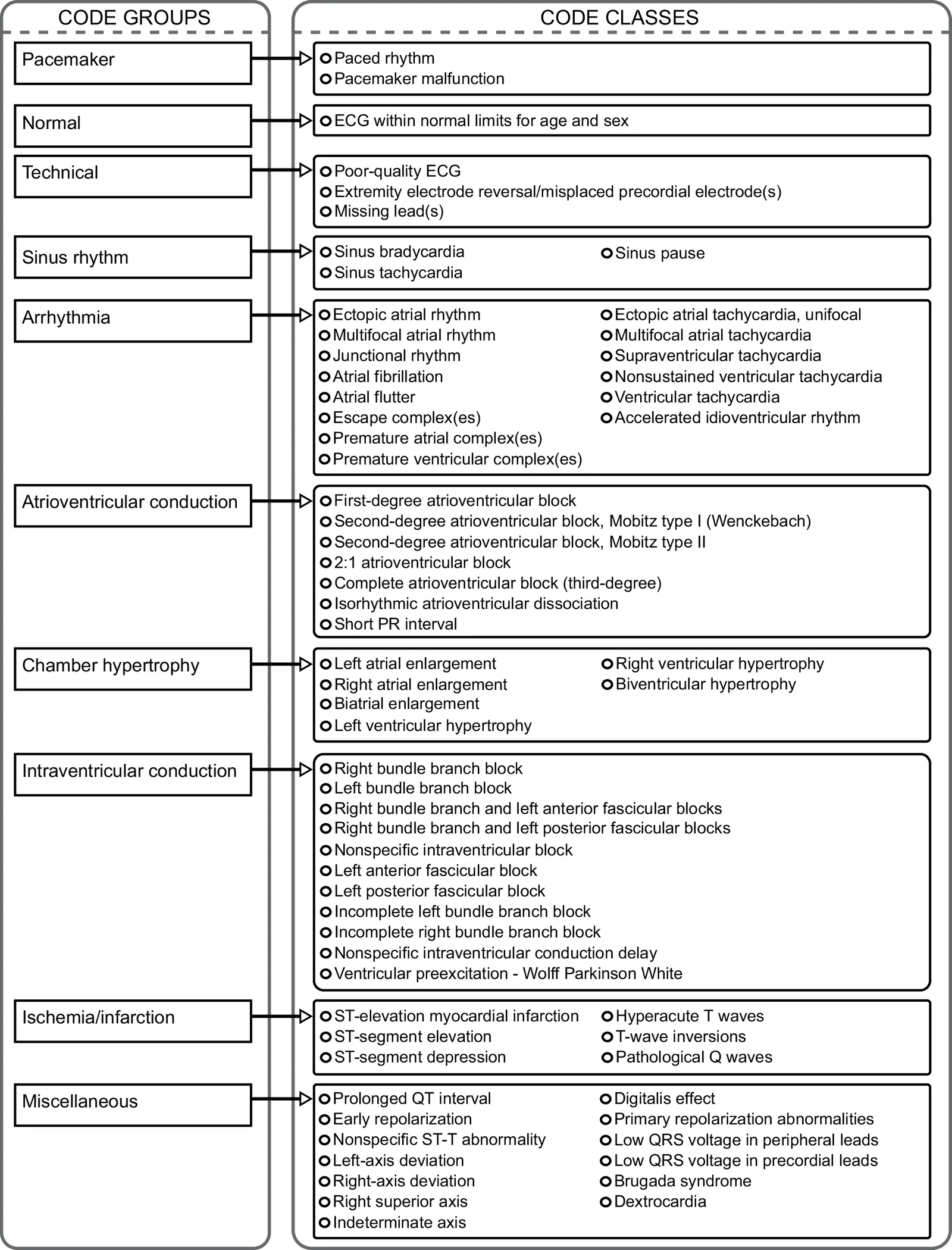
Schematic representation of the 10 groups and their corresponding 66 ECG CODE classes.

The CODE diagnostic classes were introduced in 2018 to replace free-text ECG reports. The main goal was to standardize ECG diagnosis among the cardiologist team and establish a homogeneous report for primary care physicians. The criteria used for ECG diagnosis were based on the standards of the AHA, ACC, the Heart Rhythm Society, and the International Society for Computerized Electrocardiology for ECG interpretation^19^ and the Brazilian guidelines for reporting ECG^20,21^. A total of 66 ECG classes were defined and grouped into 10 categories: Pacemaker, Normal, Technical, Sinus Rhythm, Arrhythmia, Atrioventricular Conduction, Chamber Hypertrophy, Intraventricular Conduction, Ischemia/Infarction, and Miscellaneous (see Fig. 8). The distribution and prevalence of exams for each of the 66 CODE classes are shown in Supplementary Table 2. A comprehensive description and representative examples of all CODE classes are available at: https://code.telessaude.hc.ufmg.br/.

This standardized labeling framework ensured consistent annotation of ECG exams at scale, supporting both routine clinical workflows and the development of AI models. All diagnostic labels used in this study were derived from this coding system, ensuring alignment with clinical standards and reproducibility of findings.

### Inclusion criteria and organization of the CODE-II dataset

All ECGs performed between January 2019 and December 2022 were included for evaluation. The dataset underwent two main preprocessing stages: one to assess the consistency of key exam and report information, and another to ensure the technical integrity of the ECG tracings.

**Fig. 9:**
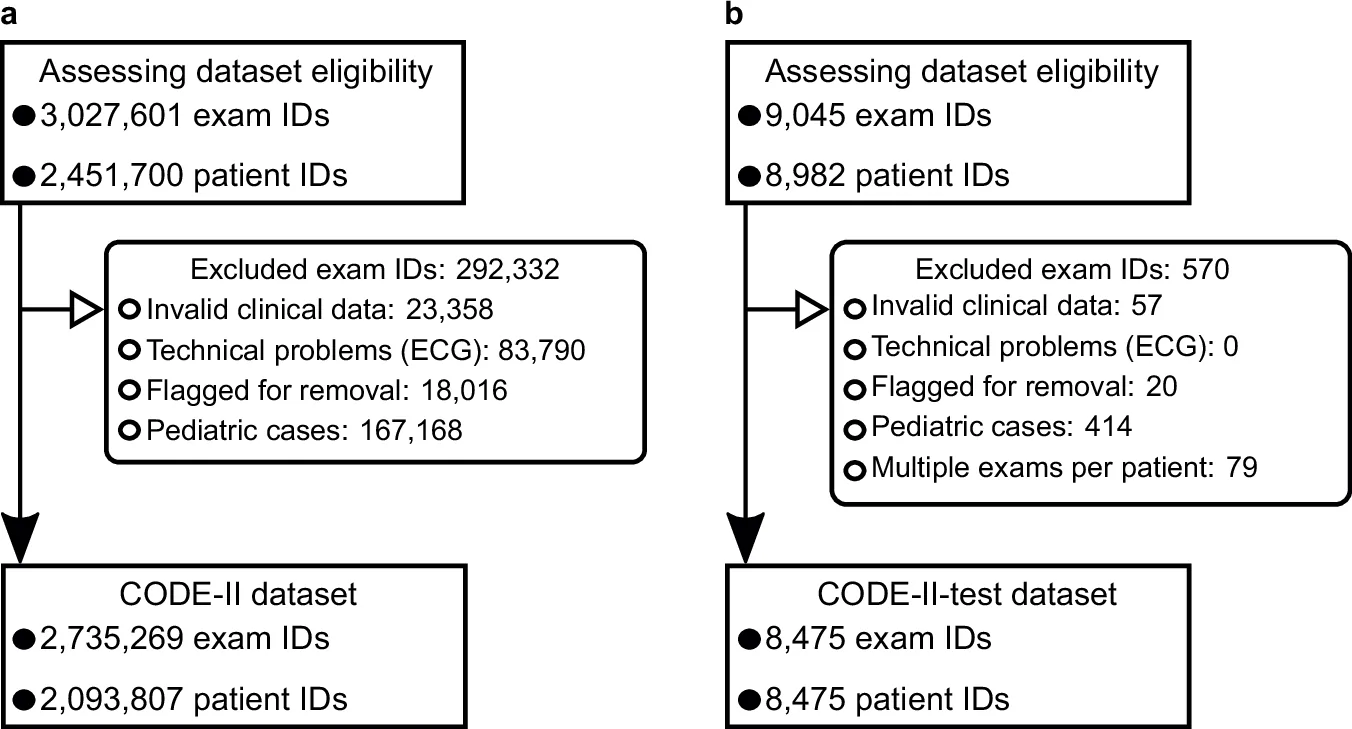
Flowcharts describing dataset curation. **a** CODE-II (full dataset) and **b** CODE-II-test. Each panel summarizes eligibility checks, exclusions, and the final number of exams and unique patients.

In the first preprocessing stage, we applied exclusion criteria based on the exam date, the patient’s date of birth, and sex. Exams with inconsistent information were removed by discarding the corresponding exam identifiers (IDs), which were deemed invalid clinical data. In the second stage, we removed exam IDs for which ECG tracings were missing or had insufficient duration in their essential leads (I, II, V1–V6) for analysis. These cases were deemed technical problems. Additionally, exams previously flagged for removal due to being linked to external research projects or because their medical reports consisted solely of medical observations or requests were removed. Exams from patients under 18 years old were also removed and deemed pediatric cases (Fig. 9).

Notably, prior to applying the preprocessing stages described above, all records underwent an internal curation process conducted by a TNMG data specialist. This process aimed to identify and link records belonging to the same individual and assign a unique patient ID, even when discrepancies in patient identifiers were present. This step helped correct inconsistencies and prevent unnecessary data loss. The specialist had access to personally identifiable information on patients from both the TNMG system and the Brazilian Public Health System (SUS), which enabled the application of multiple verification steps to accurately detect, link, and correct records. Importantly, these personal identifiers are not included in the final dataset due to privacy regulations and are accessible only to authorized personnel involved in internal data validation.

The resulting dataset, named CODE-II, is organized into three components. (i) Clinical/exam metadata: exam ID and date; patient identifiers (ID, date of birth, sex); exam location; clinical history; current medications; comorbidities; and the clinical indication for the ECG. Patient age was computed as the difference between exam date and date of birth, converted to years by dividing by 365.25 to account for leap years. (ii) Reporting and labeling data: exam and report IDs (linking to the metadata), report upload dates, cardiologist IDs, report type (original or revised), diagnostic labels and their CODE class IDs, and ECG-derived cardiac measurements. (iii) Raw signal data: 12-lead ECG waveforms accompanied by technical metadata, including the exam identifier; a sequential index for each tracing within the exam (starting at 1 and preserving the acquisition order); the sampling rate; and the signal resolution. Each tracing corresponds to a consecutive recording from the same clinical session. The total number of tracings in an exam can be inferred from the highest sequential index observed for that exam.

### The CODE-II-test

To enable the development and benchmarking of new AI applications, we curated a high-quality test dataset, named CODE-II-test, from audited ECG cases within TNMG. The dataset comprises 8475 12-lead ECGs from unique patients and was specifically designed as an independent benchmark for evaluating AI-based diagnostic models. Special attention was taken to include exams spanning also the rarer diagnostic classes, thereby covering the full spectrum of the 66 CODE classes—normal findings, technical issues that preclude analysis, and diverse abnormalities. To prevent information leakage, the CODE-II-test is non-overlapping with the CODE-II dataset at the patient level (i.e., no patient appears in both), avoiding any transfer of patient-specific features when evaluating models trained on CODE-II. The same preprocessing pipeline used for CODE-II (Fig. 9a) was applied, with an additional exclusion criterion retaining only the first ECG per patient (Fig. 9b).

A total of 46 certified cardiologists independently participated in the annotation process, each contributing to a variable number of exams depending on availability and audit allocation. Diagnostic classes were assigned using the standardized 66 CODE diagnostic classes. The Normal class is defined as mutually exclusive (i.e., it is assigned only when no abnormal diagnostic class is selected), whereas all other classes may co-occur. To characterize labeling consistency beyond the adjudication rules below, we also quantify per-class pairwise inter-rater agreement among cardiologists (Cohen’s kappa; Supplementary Table 16).

The final labels were determined using two predefined decision rules:

(i) *Agreement*. If all reviewers assigned exactly the same set of CODE diagnostic classes to an exam (complete concordance across the full multilabel set), this set was directly adopted as the final label set.
(ii) *Majority rule (label-wise)*. Otherwise, we formed the final multilabel set by applying a label-wise vote count across reviewers: for each diagnostic class, we counted how many reviewers assigned that class to the exam, and we included in the final label set every non-Normal class receiving at least two votes. If no abnormal class received ≥2 votes but the Normal class received ≥2 votes, then Normal was assigned exclusively, consistent with its mutually exclusive definition. By construction, the majority rule is applied only to exams in which at least one diagnostic class receives ≥2 votes, ensuring a non-empty final label set.

The CODE-II-test dataset was curated to include only essential metadata: exam ID, exam date, patient ID, date of birth, sex, age, specialist IDs, and the final assigned diagnoses. This design ensures both high-quality annotations and strict patient-level separation from the training and validation sets, enabling its use as an independent benchmark for AI evaluation.

### Architecture and training of the AI model for CODE classification

**Fig. 10:**
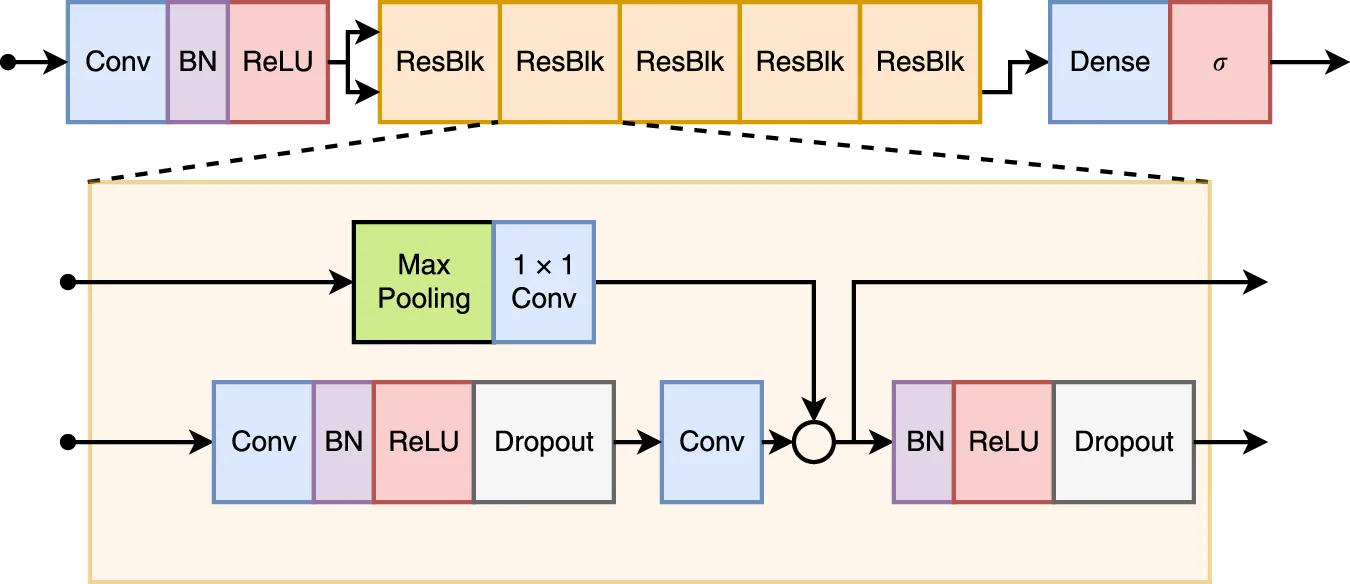
Deep neural network model architecture. Adapted from Fig. 3 in Ribeiro et al.^3^ (CC BY 4.0).

To showcase the potential of the dataset for AI applications and also for establishing a baseline for future developments, we train and test a baseline model. The architecture of the baseline model, illustrated in Fig. 10, is a deep convolutional neural network based on a residual architecture adapted from ResNet^3,22,23^. The model receives as input 8-lead ECG signals of 4096 samples and begins with an initial convolutional layer that applies 64 filters while preserving the number of samples. This is followed by batch normalization and ReLU activation, and then includes 5 residual blocks. All convolutional layers in the model, including the initial layer and those within the residual blocks, use a kernel size (filter length) of 17. Each residual block consists of 2 convolutional layers, followed by batch normalization, ReLU activations, and dropout (with a rate of 0.5). Subsampling by a factor of 4 is applied in each block, reducing the number of samples to [4096, 1024, 256, 64, 16] across the network. The number of filters increases across the blocks according to the configuration [64, 128, 196, 256, 320]. Max pooling and 1 × 1 convolutions are included in the skip connections to ensure that the number of samples and filters matches those from the signals in the main branch. The output from the final residual block is flattened and passed to a fully connected layer, which produces independent probabilities for each diagnostic class through a sigmoid activation function, in line with the multilabel nature of this classification task.

A preprocessing pipeline was applied to ensure data quality and input consistency. Since each ECG exam may contain multiple tracings recorded during the same clinical session, each lasting between 7 and 12 s and associated with the same set of diagnostic classes (CODE labels), we retained up to 4 tracings per exam to provide representative and balanced inputs (see Supplementary Fig. 5). All signals were first filtered at their native sampling rate to remove common acquisition artifacts and then resampled to 400 Hz using polyphase filtering (resample_poly): baseline drift was removed using a zero-phase elliptic high-pass filter with passband cutoff *f*_*c*_ = 0.8 Hz and stopband edge *f*_*st*_ = 0.2 Hz (passband ripple *r*_*p*_ = 0.5 dB; stopband attenuation *r*_*s*_ = 40 dB; filter order determined by ellipord and implemented in second-order sections form, with the resulting order deterministically defined by these parameters and the sampling rate and reproducible using the released preprocessing scripts), and powerline interference was suppressed using a 60 Hz notch filter with quality factor *Q* = 30 (applied with zero-phase filtering). Signals were then adjusted to a fixed length of 4096 samples per lead by centered symmetric padding or trimming: if the resampled tracing was shorter than 4096, we zero-padded it symmetrically; if longer, we center-cropped it. When an odd number of samples had to be padded/removed, the extra sample was assigned to the right by convention. From the standard 12-lead ECG, we selected the 8 essential leads (I, II, and V1–V6), preserving core diagnostic information while reducing input dimensionality. The resulting 8-lead signals for each tracing were then used as inputs to the model. The preprocessing scripts used in this study are publicly available at https://github.com/antonior92/ecg-preprocessing.

The model was trained using a binary cross-entropy loss function with equal weight assigned to each class. Model training was implemented in PyTorch using a batch size of 128, with the random seed fixed to 2 for reproducibility. Optimization was performed using the Adam algorithm with an initial learning rate of 0.001 and no weight decay. A learning rate scheduler monitored the validation loss and reduced the learning rate by a factor of 0.1 when the validation loss plateaued for 5 consecutive epochs, down to a minimum learning rate of 1 × 10^−7^. The training was conducted for up to 70 epochs or until the learning rate dropped below this minimum predefined value, and the final model was selected based on the lowest validation loss observed during this process.

Training and validation were conducted using the CODE-II dataset, comprising a total of 2,735,269 ECG exams. All partitions were constructed at the *patient level* using unique patient identifiers: each patient was assigned to exactly one split (training or validation) *before* any exam- or tracing-level expansion. Consequently, although a patient may contribute multiple ECG exams (and each exam may include up to four tracings), all records from that patient are confined to a single split, preventing patient-level leakage during model development. To obtain a validation set with broad diagnostic coverage while preserving patient exclusivity, we targeted approximately 20% of positive exams per diagnostic class in validation. Because exams are multilabel and patients may have multiple exams, we used a complexity-aware patient assignment heuristic that processes diagnostic classes from least to most frequent and prioritizes patients by diagnostic complexity (patient-level mean number of positive labels per exam), using the number of exams per patient as a secondary criterion. Under these priorities, we iterated over the ordered patients and alternated their assignment between validation and training until the per-class validation targets were reached; all remaining patients (and their exams) were then assigned to training. The resulting split comprises 2,226,443 exams (81.4%) for training and 508,826 exams (18.6%) for validation. We additionally performed a leakage check, confirming that the intersection of patient IDs between training and validation is empty. The per-diagnosis distributions achieved by this splitting strategy are reported in Supplementary Table 2. Separately, CODE-II-test is a fixed, patient-unique benchmark designed exclusively for performance evaluation (one ECG per patient) and is therefore not split for model development. We verified that all patient identifiers are unique within CODE-II-test and that the intersection of patient IDs between CODE-II and CODE-II-test is empty, ensuring strict patient-level separation between training/validation data and the independent test benchmark.

Given that each ECG exam may include up to 4 tracings, it is important to clarify how they are handled throughout model development. During training and validation, each tracing, comprising 8 leads and associated with the same diagnostic classes, is treated as an independent input, effectively serving as a form of data augmentation. Consequently, exams with more available tracings yield more training instances. During testing, inference is performed independently for each tracing, and the class-specific outputs are aggregated by averaging the predicted probabilities across all tracings, so that each exam contributes a single prediction to the reported metrics. These aggregated probabilities (i.e., the model’s output), which range from 0 to 1, are then used to determine which diagnostic classes are considered present in the exam. A class is classified as present when its aggregated probability exceeds a predefined threshold. In this work, the threshold for each class was selected from its precision–recall curve on the CODE-II validation set, which is part of the model development process. The final performance was then assessed on the independent CODE-II-test dataset. The threshold selection strategies, along with the evaluation metrics, are detailed in the following subsection.

### Evaluation metrics and threshold selection

To evaluate model performance on the CODE-II-test, we employed metrics commonly used in multilabel classification tasks. Threshold-independent metrics included the AUROC and the AUPRC. Both were computed per diagnostic class as well as in micro- and macro-averaged forms. AUROC quantifies the model’s ability to discriminate between positive and negative instances across all decision thresholds and is less affected by class imbalance. In contrast, AUPRC emphasizes the balance between precision and recall (sensitivity), making it particularly informative for rare diagnostic classes. To contextualize AUPRC values, we report the prevalence of each diagnostic class in the test set, which serves as a theoretical reference point for interpretation, as it represents the AUPRC expected from a random classifier. For example, an AUPRC of 0.60 is considered strong for a class with 5% prevalence, since the ratio between the AUPRC and the class prevalence (0.60/0.05) indicates that the model performs 12 times better than random. In contrast, for a class with 50% prevalence, the same score corresponds to a much smaller improvement (0.60/0.50 = 1.2), suggesting only a modest gain over chance.

Threshold-dependent metrics comprised precision, recall, specificity, F1-score, and NPV. These were calculated per diagnostic class and summarized globally using two complementary schemes: micro-averaging, which aggregates true positives, false positives, and false negatives across all classes; and macro-averaging, which averages metrics computed independently for each class. Importantly, these threshold-dependent metrics require selecting operating points that should be guided by the intended clinical use case (e.g., screening, triage, or diagnostic assistance) and by the relative costs of false positives and false negatives.

For threshold selection, our *primary* evaluation protocol adopts a class-specific strategy that maximizes the F1-score (F1-max), a widely used approach that identifies operating points balancing precision and recall within each diagnostic class. This serves as a standardized baseline, particularly suitable when both false positives and false negatives carry clinical relevance. Class-specific thresholds are selected on the CODE-II validation set and then fixed and applied unchanged to the CODE-II-test set without further tuning. While this preserves an honest evaluation, it also makes threshold-dependent metrics sensitive to shifts in class prevalence and score calibration between validation and test. As a sensitivity analysis (reported in the Supplementary Information), we compare F1-max with an alternative thresholding rule based on Youden’s J statistic, defined as *J* = sensitivity + specificity − 1, where sensitivity is equivalent to recall, for micro/macro aggregates and a small subset of classes. This analysis is provided to illustrate threshold sensitivity and does not change the primary protocol above.

### Evaluation protocol on external ECG benchmarks

To assess the generalizability of the representations learned by our model, we conducted a series of experiments using our model pre-trained on CODE-II. The goal was to evaluate whether this model, originally developed for multilabel classification with CODE diagnostic classes, could yield transferable and discriminative features when applied to external public ECG datasets with different populations and diagnostic labels.

We selected two publicly available ECG datasets for this evaluation. The first, the Physikalisch-Technische Bundesanstalt ECG dataset (PTB-XL), comprises 21,837 clinical 12-lead ECGs from 18,885 patients collected in Germany^15^. Diagnostic annotations are provided at multiple levels of granularity, including 44 distinct diagnostic classes, which can be aggregated into broader superclass and subclass categories. Additionally, this dataset includes 19 form statements describing ECG waveform morphology and 12 rhythm statements related to cardiac rhythm. To further evaluate model transferability, we constructed more challenging training scenarios by using only 5% and 10% subsets of the PTB-XL training set. These subsets maintain a label distribution similar to that of the full dataset, allowing for a controlled assessment of performance under limited data availability. The second dataset, the China Physiological Signal Challenge 2018 (CPSC 2018), consists of 9831 ECG recordings from 9458 patients in China, annotated into 9 diagnostic classes, covering common arrhythmias and conduction disorders^25^. Both datasets were selected due to their expert-reviewed labels, diagnostic diversity, and frequent use in benchmarking ECG classification methods. Importantly, they differ from CODE-II not only in labeling schemes but also in patient populations and acquisition environments, making them well-suited for evaluating the robustness and transferability of the learned representations. Moreover, the ability of the model to generalize across these heterogeneous datasets indirectly reflects the diagnostic breadth and quality of the CODE-II dataset used for training.

We compared our trained model with two baseline settings: (i) supervised models trained from scratch, and (ii) large pre-trained ECG models fine-tuned on our target dataset. The supervised models are both effective and lightweight, with parameter counts ranging from 0.45 million to 2.35 million. These include LSTM^40^, BiLSTM^41^, XResNet1d101^42^, ResNet1d-Wang^43^, and InceptionTime^44^. The pre-trained ECG models, including ResNet1d-Lima^6^, ResNet1d-Ribeiro^3^, ResNet1d-Merl, and ViT1d-Merl^45^, ECG-FM^27^, Heartlang^28^, and the HuBERT-Small and HuBERT-Base models^26^, are publicly accessible and encompass both Transformer-based and ResNet-based architectures, with parameter counts ranging from 3.87 million to 92.83 million. These models have demonstrated strong generalization capabilities and were pre-trained on both public and access-on-demand ECG datasets. For example, HuBERT was pre-trained on 9.1 million ECG recordings from datasets such as CODE-I^3^, MIMIC-IV ECG^46^, and the Chapman-Shaoxing ECG dataset^47^, which is nearly 4 times the size of CODE-II. ResNet1d-Merl and ViT1d-Merl were both trained using the MERL framework, which applies a multimodal learning strategy to approximately 0.8 million ECG-report pairs from the MIMIC-IV ECG dataset. ResNet1d-Merl employs a 1D ResNet18 as the ECG encoder, while ViT1d-Merl substitutes this component with a Vision Transformer-based encoder.

We established a standardized evaluation pipeline to ensure fair and reproducible comparisons. Following the PTB-XL setup^15^, we adopted the same 8:1:1 train/validation/test split and preprocessing pipeline. Models were trained for up to 100 epochs with early stopping based on validation macro-AUROC, using a patience of 5. The model with the highest validation AUROC was selected for final evaluation. We used binary cross-entropy loss and optimized with Rectified Adam. For supervised baselines, we adopted implementations and hyperparameters from ref. ^48^, fixing the learning rate to 0.001 for consistency. For pre-trained models, we applied full-model fine-tuning and replaced the original projection head with a linear classification layer to enable downstream evaluation. To ensure compatibility with the respective pre-training settings, ECG signals were resampled to match the frequency and lead configurations used in each model: 100 Hz for Heartlang, 500 Hz for ResNet1d-Merl, ViT1d-Merl, HuBERT-Small, HuBERT-Base, and ECG-FM, and 400 Hz for ResNet1d-Lima, ResNet1d-Ribeiro, and ours. Given the sensitivity of large models to learning rate, we performed a grid search over [0.001, 0.0001, 0.00001, 0.000001] on the validation set to identify the optimal value for each model. We adopted macro-AUROC as the primary evaluation metric on the test set, as it is threshold-independent and widely used, thereby avoiding bias introduced by fixed threshold selection. Detailed descriptions and configurations of the baselines are provided in Supplementary Tables 13 and 14.

### CODE-II-open

The CODE-II-open dataset is a curated subset of the full CODE-II, developed as part of this study to serve as a public benchmark for training, validation, and reproducibility of deep learning models for ECG classification based on the 66 expert-defined CODE diagnostic classes. While the CODE-II dataset was used for model development and internal evaluation, the CODE-II-open was specifically created to support reproducible experimentation and external use.

CODE-II-open comprises 15,000 12-lead ECG exams, each corresponding to the first exam of a unique patient, representing approximately 0.72% of all patients included in the full CODE-II dataset. Although this may appear to be a limited sample, this sample size was deliberately chosen to align with the inflection point observed in the scaling law curves (see Fig. 5), where model performance transitions from steep to incremental gains, as detailed in the sections “Scaling laws in CODE-II: How training dataset size influences model performance” and “Scaling law experiments.” In addition to this empirical motivation, rigorous selection criteria were applied to ensure the dataset supports reproducible experimentation and serves as a representative benchmark subset of the full CODE-II dataset used to develop our baseline model for the 66 CODE diagnostic classes. Specifically, CODE-II-open was partitioned into 12,000 training and 3,000 validation exams (80/20 split), sampled exclusively from the patient-exclusive training and validation subsets of the full dataset after restricting the dataset to the first exams per patient. Diagnoses were processed from rarest to most common, and within each diagnostic class, exams were randomly sampled using a fixed random seed up to class-guided targets constrained by availability, aiming to improve class coverage while preserving the intended 80/20 split. When additional exams were needed to reach the target size of 15,000, they were filled using exams from the most frequent class (NORMAL), consistent with its high prevalence in routine clinical ECGs, while maintaining the original training/validation partition. The resulting class-wise distribution is presented in Supplementary Table 5.

Each exam in the CODE-II-open dataset is accompanied by two main sets of information. The first includes metadata related to the ECG acquisition, such as the exam ID, exam date, patient ID, date of birth, sex, reported comorbidities, and clinical indication for the ECG. Patient age was recalculated as the difference between exam and birth dates, expressed in years by dividing the result in days by 365.25 to account for leap years. The second set comprises diagnostic and reporting data, including the report upload date, type of report (original or revised), electrocardiographic measurements, and the set of diagnostic classes assigned to the exam. These diagnoses correspond to the 66 CODE diagnostic classes, including their respective class IDs and labels.

For each exam, up to four *raw* 12-lead ECG tracings are provided, corresponding to recordings captured during the same clinical session and lasting between 7 and 12 s. Depending on the acquisition device, tracings were sampled at 300 Hz, 500 Hz, 600 Hz, or 1000 Hz. To ensure a minimum quality standard in the released files, tracings were selected using an automated pipeline that excluded corrupted or structurally inconsistent signals, with up to four recordings retained per exam. The preprocessing configuration used in our baseline experiments (including re-sampling, length normalization, filtering, and lead selection) is specified in the “Methods” section (“Architecture and training of the AI model for CODE classification”) and can be reproduced using the publicly available scripts at https://github.com/antonior92/ecg-preprocessing.

### Scaling law experiments

We investigated the effect of the dataset size used for model development on model performance through a series of scaling law experiments. In this analysis, we restricted the CODE-II dataset to the first ECG recorded for each patient, resulting in 2,093,807 unique patients and corresponding exams. From this patient-level dataset, we generated multiple training-validation splits of increasing size, including subsets ranging from 1000 to 50,000 patients, the publicly released CODE-II-open (15,000 patients), and approximately 5%, 10%, 25%, and 50% of the full patient-level dataset. All splits maintained an approximately 80/20 train-validation ratio by applying the splitting heuristic proposed in this study, which aims to preserve this proportion within each diagnosis class and ensure patient exclusivity across training and validation sets. CODE-II-open was designed with 15,000 patients to align with the inflection point of the scaling curve, where performance transitions from steep to incremental gains. To better capture this transition and evaluate post-inflection behavior, experiments were extended up to approximately 50% of the dataset. All evaluations were performed on the fixed CODE-II-test set to enable fair and consistent comparisons.

For all scaling law experiments, the primary experimental variable was the dataset size used for model development, while the model architecture and training hyperparameters were kept fixed. For each dataset size, three independent runs were performed using different random seeds (1, 2, and 3) to assess variability arising from model weight initialization and the random ordering of training examples into mini-batches at the start of each epoch. Model performance was evaluated on the fixed CODE-II-test set using macro-AUROC and macro-AUPRC. For each dataset size, we report the mean, minimum, and maximum values of these metrics across the three runs.

### Ethics statement

This study was conducted in accordance with all relevant ethical regulations and was approved by the Research Ethics Committee of the Universidade Federal de Minas Gerais (CAAE: 85892325.1.0000.5149). Analyses were performed using two datasets from the TNMG: the CODE-II dataset, used for model development, and the CODE-II-test dataset, used for independent evaluation. Both datasets consist of anonymized 12-lead ECG recordings and associated metadata collected during routine clinical care. As this was a secondary analysis of anonymized data, the Research Ethics Committee waived the requirement for individual informed consent. No identifiable images of human participants are included in this study. All researchers involved in this work signed confidentiality and data use agreements prior to accessing the data.

## Supplementary information

Supplementary Information

Supplementary Data 1

Supplementary Data 2

Supplementary Data 3

Supplementary Data 4

Supplementary Data 5

Supplementary Data 6

Supplementary Data 7

## Data Availability

The CODE-II-open dataset, a subset of the full CODE-II database comprising 15,000 unique-patient ECG exams annotated with the 66 expert-defined CODE diagnostic classes, is publicly available via PhysioNet for non commercial use. The ECG signals from the CODE-II-test dataset (8475 unique patients) are also publicly available via PhysioNet for non commercial use. The corresponding expert annotations for CODE-II-test are reserved for benchmarking and are not publicly released; however, any user may submit model predictions for evaluation under controlled access. See https://github.com/PetrusAbreu/code-ii for the link and instructions. Access to the full CODE-II dataset is restricted. Requests to access these data are considered on an individual basis by the Telehealth Network of Minas Gerais (telessaude.hc-ufmg@ebserh.gov.br). Requests should be sent by email with the corresponding authors in copy. Any data use is restricted to non-commercial research purposes and requires the execution of appropriate data use agreements. Supplementary Information provides extended descriptions of the datasets, along with additional figures and tables detailing patient- and exam-level characteristics. Source data underlying the figures and tables in the main text are provided as a Source Data file, except for those derived from restricted datasets, which are available upon request under the same access conditions described above.

## Code availability

Code for ECG signal preprocessing is openly available at https://github.com/antonior92/ecg-preprocessing. The neural network architecture used in this study is based on the model originally proposed for the CODE-I dataset and available at https://github.com/antonior92/automatic-ecg-diagnosis. In addition, we evaluated several publicly available large pre-trained ECG models, including ResNet1d-Lima https://github.com/antonior92/ecg-age-prediction, ResNet1d-Ribeiro https://github.com/antonior92/automatic-ecg-diagnosis, ResNet1d-Merl and ViT1d-Merl https://github.com/cheliu-computation/MERL-ICML2024, ECG-FM https://github.com/bowang-lab/ecg-fm, HeartLang https://github.com/PKUDigitalHealth/HeartLang, and HuBERT-Small and HuBERT-Base https://github.com/Edoar-do/HuBERT-ECG. Corresponding references are provided throughout the manuscript. The full model weights trained on the complete CODE-II dataset are licensed to UFMG, which is responsible for their stewardship and controlled distribution. Basemap polygons used to generate Fig. 1 were obtained from the Click That ’Hood project (brazil-states.geojson; MIT License) and retrieved from https://raw.githubusercontent.com/codeforamerica/click_that_hood/master/public/data/brazil-states.geojson.
