# Supplementary material for: CODE-II: a large-scale dataset for artificial intelligence in ECG analysis
Source: NPJ Digit Med. 2026 May 26;9:658. doi: 10.1038/s41746-026-02704-4 (PMC13519028; doi:10.1038/s41746-026-02704-4)
Supplement: Supplementary file 1 [file 41746_2026_2704_MOESM1_ESM.pdf]

Supplementary Information

CODE-II: A large-scale dataset for artificial intelligence in ECG analysis

Petrus E. O. G. B. Abreu[1,*], **Gabriela M. M. Paixão**[1], **Jiawei Li**[2], **Paulo R. Gomes**[1], **Peter W. Macfarlane**[3], **Ana C. S. Oliveira**[1], **Vinícius T. Carvalho**[1], **Thomas B. Schön**[2], **Antonio Luiz P. Ribeiro**[1,4], **and Antônio H. Ribeiro**[1,2,*]

[1]Universidade Federal de Minas Gerais (UFMG), Brazil
[2]Uppsala University, Sweden
[3]University of Glasgow, Scotland
[4]Telehealth Center, Hospital das Clínicas, UFMG, Brazil

[*]Corresponding authors: petrusabreu@ufmg.br, antonio.ribeiro@ebserh.gov.br, antonio.horta.ribeiro@it.uu.se

A CODE-II Dataset: Extended Description

This section provides supplementary information supporting the description presented in the main manuscript about the curated CODE-II dataset, composed of 2,735,269 ECG exams from 2,093,807 unique patients collected and annotated by TNMG between January 2019 and December 2022. The figures and tables below expand the dataset characterization by offering a more granular view of patient-level attributes and exam characteristics, as well as presenting additional information available in the dataset but not detailed in the main text.

A.1 General Characteristics

Supplementary Fig. 1 presents the distribution of the 2,735,269 ECG exams across the four-year data collection period, providing temporal context. The annual volume remained relatively stable, with a slight decline in 2020, likely reflecting the impact of the COVID-19 pandemic on routine healthcare services.

We also stratified the 2,093,807 patients by age group and sex, considering only their first ECG exam. Among patients aged 18–59 years (62.2% of all patients), females accounted for 796,170 (61.1%) and males for 506,382 (38,9%). In the older group (≥ 60 years; 37.8% of patients), there were 441,462 females (55.8%) and 349,793 males (44.2%). Overall, based on first exams, 59.1% of patients were female (1,237,632) and 40.9% were male (856,175). The full age distribuition by sex is shown in Supplementary Fig. 2.

Self-reported clinical comorbidities of the CODE-II dataset, stratified by age (18–59 years and ≥ 60 years) and sex, are detailed in Supplementary Table 1. As reported in the main text, hypertension was the most prevalent comorbidity, affecting 47.7% of patients, followed by diabetes mellitus (14.0%), smoking (8.2%), dyslipidemia (4.8%), previous myocardial infarction (1.8%), Chagas disease (1.1%), and chronic obstructive pulmonary disease (0.7%). Patients could report more than one comorbidity. Comorbidities and medication usage were recorded using checkboxes marked only when present, meaning that unmarked fields may indicate either missing information or the true absence of the condition or medication use. Medication data were also used to infer the presence of certain conditions: patients reporting the use of calcium channel blockers, diuretics, and/or angiotensin-converting enzyme inhibitors were regarded as having hypertension; those using statins as having dyslipidemia; and those taking insulin or oral hypoglycemic agents as having diabetes.

Clinical indications for ECG exams were recorded using checkbox fields, allowing multiple symptoms to be reported per patient. The most frequently indicated reasons were routine examinations and chest pain, followed by preoperative risk assessment, palpitations, dyspnea, and "other". The "other" category corresponded to a free-text field in which healthcare professionals could specify additional symptoms not covered by predefined options; this field was completed in 6.4% of the exams. The full distribution of clinical indications, stratified by age and sex, is shown in Supplementary Fig. 3.

In addition, we investigated how many ECG exams each patient underwent over the study period. As illustrated in Supplementary Fig. 4a, a majority (1,663,122 patients, 79.4%) had a single exam, although a significant proportion underwent multiple recordings, with some patients having more than ten exams. Supplementary Fig. 4b shows the number of signal records per exam. These represent multiple consecutive tracings (lasting between 7-12 seconds each) recorded during a single clinical session. All tracings were available to TNMG physicians for diagnostic interpretation, and each exam was annotated with one or more labels from the 66 CODE diagnostic classes.

To illustrate the final number of tracings retained in the CODE-II dataset after applying the pipeline that selects up to four tracings per exam to ensure representative and balanced inputs, Supplementary Fig. 5 presents a summary of this distribution.

The 66 CODE diagnostic classes used by physicians to annotate ECGs in the CODE-II dataset were introduced in the main manuscript, together with an overview of their clinical relevance and frequency distribution. Here, we provide a detailed summary of these diagnostic classes, including their descriptions, an associated label (defined solely for ease of reference in figures), their overall prevalence in the dataset, and the class-wise distribution across the training and validation sets (approximately 80/20 split), based on the patient-exclusive split strategy adopted during model development. These details are presented in Supplementary Table 2.

We also present a group-level analysis of diagnostic label assignments. The 66 classes are grouped into 10 clinically meaningful diagnostic groups. Supplementary Fig. 6 provides an analysis of: (a) the number of exams exclusively assigned to a single diagnostic group, (b) the 10 most frequent non-exclusive group combinations observed across all exams, and (c) the distribution of the number of CODE groups assigned per exam. This figure complements the main text by quantifying the combinatorial diversity of diagnostic group assignments.

To further characterize the diagnostic landscape of the dataset, we analyzed the distribution of diagnostic groups at the patient level. Supplementary Table 3 presents this analysis using only the first ECG exam per patient, stratified by age and sex. Group assignments are not mutually exclusive—except for the Normal group, which is exclusive by definition—allowing patients to appear in multiple categories. This table complements the population-level description in the main manuscript and provides additional insight into the prevalence of diagnostic groups across demographic subgroups.

We also analyzed electrocardiographic interval measurements extracted from the 12-lead ECG signals in the CODE-II dataset. These measurements revealed clear sex- and age-related differences, consistent with known physiological patterns and prior population-based studies [1;2]. Female patients consistently exhibited higher resting heart rates and longer corrected QT intervals (QTc), while male patients showed longer depolarization and conduction intervals, including P-wave duration, PR interval, and QRS duration. For instance, median heart rates among women were 73 bpm (18-59 years) and 72 bpm (60+ years), compared to 68 bpm in both age groups for men. Median QTc intervals ranged from 420 to 427 ms in women and from 410 to 420 ms in men. In contrast, median PR intervals and QRS durations were longer in men across both age groups (e.g., PR: 152–158 ms vs 146–152 ms; QRS: 96–98 ms vs 90–92 ms in men vs women). These findings are consistent with prior Brazilian population studies, such as the ELSA-Brasil study and a large primary care cohort [1;2], and large international studies have likewise emphasized the impact of age and sex on ECG norms [3;4]. Additionally, we observed an age-related leftward shift of the frontal QRS axis, particularly among women, in line with known aging effects on cardiac electrophysiology. Although these electrocardiographic measures are not the central focus of this study, their internal consistency and physiological plausibility further support the representativeness of the CODE-II dataset. Readers interested in more in-depth analyses of ECG interval characteristics are referred to comprehensive population-based studies [3;4]. Supplementary Fig. 7 displays the full distribution of ECG intervals stratified by age group and sex. Notably, the proportion of missing values for these measurements is very low in the CODE-II dataset, with fewer than 0.16% missing in any subgroup when analyzed by sex.

B CODE-II-open Dataset Characterization

This supplementary section complements the overview provided in the main manuscript, offering a detailed characterization of the CODE-II-open dataset. We describe the demographic and clinical attributes of the 15,000 patients included, along with their associated diagnostic annotations. Additional information is provided regarding comorbidity prevalence, clinical indications for the ECG exams, and electrocardiographic measurements, all stratified by age and sex whenever relevant. Figures and tables included herein support reproducibility and transparency for studies leveraging the CODE-II-open dataset as a public benchmark.

B.1 General Characteristics

To provide a comprehensive overview of the CODE-II-open dataset, we analyzed key characteristics of its 15,000 unique patients and their corresponding ECG exams. These exams were selected from the full CODE-II dataset and span the period between January 2019 and December 2022, thus preserving the

original dataset's temporal coverage, as illustrated in Supplementary Fig. 8. While the full dataset includes exams from 12 Brazilian states, the CODE-II-open subset covers exams from 11 states; see Supplementary Fig. 9. This geographic distribution reflects the dataset's targeted selection strategy, which prioritized diagnostic class diversity and patient uniqueness, without explicitly enforcing territorial representation.

Among the 15,000 patients included in the CODE-II-open dataset, 52.9% (7,937) were female and 47.1% (7,063) were male. The overall mean age was 55.2 years, with similar distributions for female and males (55.1 and 55.4 years, respectively), as illustrated in Supplementary Fig. 10. Patients were categorized into two age groups: 18–59 years (57.8% of the cohort), with 4,727 (54.5%) females and 3,943 (45.5%) males; and 60 years or older (42.2% of the cohort), with 3,210 (50.71%) females and 3,120 (49.29%) males. No patients under 18 years of age were included.

The CODE-II-open dataset contains detailed information on patient comorbidities, recorded through checkbox fields marked only when a condition was present. These conditions were either self-reported or inferred from medication use, while unmarked fields indicated either the absence of the condition or missing information. Hypertension was the most frequently reported comorbidity, affecting approximately 47.5% of patients, followed by diabetes mellitus (13.3%), smoking (8.9%), and dyslipidemia (4.8%). Less common conditions included previous myocardial infarction (2.3%), Chagas disease (2.1%), and chronic obstructive pulmonary disease (COPD, 1.0%). Comorbidity prevalence increased with age, as evidenced by the higher number of reported cases among patients aged 60 or older. For instance, hypertension cases rose by 39% among females (from 1,676 to 2,326) and by 72% among males (from 1,151 to 1,974). Similarly, diabetes cases increased by 80% in females (from 429 to 771) and by 106% in males (from 260 to 535) when comparing patients aged 18–59 to those aged 60 or older. Regarding exam priority, 69.7% of the ECGs were recorded as elective, 29.3% as urgent, and 1.0% as preferential. Supplementary Table 4 details comorbidity distributions across age groups and sex.

As previously mentioned, the CODE-II-open dataset includes only the first ECG for each of the 15,000 patients. Each exam may contain multiple consecutive tracings, recorded during a single clinical session and lasting between 7-12 seconds (Supplementary Fig. 12). In accordance with the preprocessing procedure outlined in the main manuscript, we retained up to four tracings per exam (Supplementary Fig. 13), selected to ensure representative and balanced inputs, given that all tracings share the same diagnostic labels. This standardized subset of tracings constitutes the final version of the dataset made available for model development and evaluation.

Each ECG exam in the CODE-II-open dataset may be associated with one or more diagnostic labels from the 66 expert-defined CODE classes (Supplementary Fig. 14), which were introduced by TNMG cardiologists to replace free-text ECG reports and promote diagnostic standardization. A comprehensive summary of these diagnostic classes—including their descriptions, overall prevalence in the dataset, and class-wise distribution across training and validation sets—is presented in Supplementary Table 5. Importantly, the training and validation subsets in CODE-II-open were derived exclusively from the corresponding partitions of the full CODE-II dataset (approximately 80/20 split), which were used to develop the baseline model described in the main manuscript. This approach ensures consistency with the original study design and full compatibility between datasets.

The 66 diagnostic classes can be grouped into 10 clinically meaningful CODE categories, for which we also analyzed exam distributions within the CODE-II-open dataset. The most frequent diagnostic groups were Normal (44.1%), Miscellaneous (21.1%), and Arrhythmia (18.7%), followed by Intraventricular Conduction Disorders, Chamber Hypertrophy, and Atrioventricular Conduction Disorders (Supplementary Fig. 15a). Most exams (85.8%) were associated with one or two diagnostic classes, while only a small fraction (1.7%) included five or more (Supplementary Fig. 15b). To provide a more detailed group-level view of diagnostic label assignment, we further examined the number of exams exclusively assigned to each group. Notably, the Normal group is the only mutually exclusive CODE category, composed of a single diagnostic class. Supplementary Fig. 16 presents the number of exams exclusively assigned to each group, the ten most frequent combinations of non-exclusive group assignments (out of 147 observed patterns), and the distribution of the number of groups assigned per exam.

The CODE-II-open dataset also includes electrocardiographic interval measurements derived from the 12-lead ECG signals. These include heart rate, P-wave duration, PR interval, QRS duration, QRS axis, and corrected QT interval (QTc), with distributions stratified by age group and sex. Although not the primary focus of this dataset, these cardiac measures provide additional evidence of internal consistency. As observed in the full CODE-II dataset, the distributions in CODE-II-open reveal physiologically plausible differences by age and sex. For instance, resting heart rate and QTc intervals were generally higher in females, while males showed longer conduction intervals such as PR and QRS durations. The median QTc ranged from 421–430 ms in women and 412–424 ms in men; QRS durations were longer in males

across both age groups. These findings mirror those from the full dataset and are consistent with known population-level trends. The proportion of missing values for these measurements is very low, with fewer than 0.2% missing in any age-sex subgroup. Supplementary Fig. 17 summarizes the full distribution of cardiac measurements across age and sex groups.

C CODE-II-test Dataset Overview

This supplementary section provides an expanded description of the CODE-II-test dataset, developed as a high-quality benchmark for evaluating AI-based ECG classification models. It comprises 8,475 exams from unique patients, collected by TNMG between 2018 and 2025, all distinct from those included in the CODE-II dataset. To ensure data quality and consistency, the same rigorous preprocessing pipeline used for CODE-II was applied. Each exam was reviewed by multiple certified cardiologists and annotated using the standardized set of 66 CODE diagnostic classes. Final diagnoses were derived according to predefined agreement and majority rules. Briefly, if all reviewers assigned the same full multi-label set, that set was adopted; otherwise, a label-wise vote was applied and the final label set included every CODE class receiving at least two votes (with the Normal class assigned exclusively when applicable; see Methods in the main manuscript). In addition to dataset characterization, we report cardiologist performance and baseline model results on this test set. The supplementary figures and tables provide a comprehensive overview, reinforcing the role of CODE-II-test as a robust and transparent benchmark for model evaluation under expert annotation.

C.1 General Characteristics

To provide a comprehensive characterization of the CODE-II-test dataset, we analyzed the demographic and clinical attributes of its 8,475 unique patients. The exams were collected between 2018 and 2025, with their annual distribution shown in Supplementary Fig. 18, and originated from 15 Brazilian states, as illustrated in Supplementary Fig. 19. Both the collection period and the geographic coverage are broader than those of the CODE-II dataset, reflecting the design of the CODE-II-test to capture a diverse set of exams with multiple high-quality annotations.

The dataset comprised 57.5% females (4,871) and 42.5% males (3,604), with an overall mean age of 55.5 years (55.3 in females and 55.7 in males). No patients under 18 years of age were included. Most patients (58.6%) were aged 18–59 years (2,898 females and 2,068 males), while 41.4% were 60 years or older (1,973 females and 1,536 males). These demographic distributions are broadly consistent with those of the CODE-II training and validation cohorts but reflect the distinct curation process of the CODE-II-test, designed as an independent benchmark under expert annotation. The age distribution stratified by sex is presented in Supplementary Fig. 20.

We next examined clinical comorbidities, which were recorded through checkbox fields, completed only when a condition was present, either self-reported or inferred from medication use. Hypertension (43.9%), Chagas disease (14.4%), and diabetes mellitus (11.6%) were the most frequent conditions, while smoking (7.3%), dyslipidemia (6.0%), previous myocardial infarction (2.2%), and COPD (0.9%) were less common. Prevalence patterns varied by age: several conditions, such as hypertension and diabetes, were more frequent among older patients, whereas smoking showed higher prevalence in younger groups. For example, hypertension was reported in 1,244 females aged 60+ compared with 1,006 in younger females, while smoking affected 212 younger males compared with 144 older males. Regarding exam priority, most ECGs were recorded as elective (73.5%), followed by urgent (26.1%) and preferential (0.4%). Supplementary Table 6 provides full comorbidity distributions stratified by age and sex.

The clinical indications for performing ECG exams were also recorded through checkbox fields, with multiple indications allowed per patient. Routine examinations were the most frequent (40.9%), followed by chest pain (16.8%), "other" reasons (9.1%)—a free-text field that allowed healthcare professionals to specify indications not covered by predefined options—palpitations (8.0%), dyspnea (6.8%), and preoperative surgical risk assessment (6.7%). These patterns were generally consistent across age and sex groups, with some variation, such as dyspnea being more common in older patients. The full distribution of indications stratified by sex and age is shown in Supplementary Fig. 21.

Each patient contributed only one ECG exam, consistent with the role of the CODE-II-test as a fixed, patient-exclusive evaluation set. Each exam may comprise multiple consecutive tracings, recorded during a single clinical session and lasting between 7 and 12 seconds (Supplementary Fig. 22). In accordance with the preprocessing strategy described in the main manuscript, up to four tracings were retained per exam in the final dataset to ensure representativeness while preserving standardized inputs (Supplementary

Fig. 23). This design guarantees strict patient-level independence from the training and validation datasets, reinforcing its role as a reproducible benchmark for model evaluation.

Diagnostic annotations were based on the 66 standardized CODE classes (Supplementary Fig. 24). Of these, 65 are not mutually exclusive, while the Normal class is uniquely assigned. This standardized structure ensures consistency and clinical interpretability across annotations. Supplementary Table 7 summarizes all classes with their labels, descriptions, prevalence, and dataset distribution.

As outlined in the main manuscript, the 66 diagnostic classes can be aggregated into 10 clinically meaningful CODE groups. Within the CODE-II-test dataset, the most frequent groups were Normal, Miscellaneous, and Arrhythmia (Supplementary Fig. 25a). Most exams were associated with one or two diagnostic classes, whereas only a small proportion involved more complex profiles with multiple labels (Supplementary Fig. 25b). At the group level, the Normal category remained the only mutually exclusive group, consisting of a single diagnostic class. Supplementary Fig. 26 presents the number of exams exclusively assigned to each group, the ten most common non-exclusive group combinations, and the overall distribution of group counts per exam.

C.2 Cardiologist Performance Evaluation

A total of 46 certified cardiologists participated in the annotation of the CODE-II-test dataset, each contributing to a variable number of exams depending on their availability and audit allocation. Participation was markedly heterogeneous: while some specialists reviewed thousands of exams, others contributed to only a few (Supplementary Fig. 27). Every exam was independently reviewed by at least two cardiologists, with most being assessed by two or three reviewers, and a smaller fraction by four or more (Supplementary Fig. 28). The diagnostic process relied on the standardized set of 66 CODE classes, which include normal findings, technical issues preventing analysis, and a broad range of abnormalities. Importantly, the Normal class was assigned exclusively, while all other classes could co-occur within the same exam.

Final diagnoses were determined using two predefined decision rules. Complete concordance among all reviewers across the full assigned multi-label set defined the agreement rule, which accounted for 6,309 exams (74.4%). For the remaining 2,166 cases (25.6%), we applied a label-wise majority vote: for each CODE class, we counted the number of reviewers assigning that class and included in the final label set every class receiving at least two votes. Consistent with its mutually exclusive definition, whenever the Normal class received at least two votes it was assigned exclusively. As a result, every exam in the dataset was assigned a definitive diagnostic label set, ensuring comprehensive coverage under expert supervision. Most exams in the CODE-II-test dataset were associated with one or two diagnostic labels, while a smaller subset presented more complex profiles with multiple abnormalities (Supplementary Fig. 25b). This distribution, described in detail in the General Characteristics section, reflects the variability of clinical findings captured in the dataset.

To further characterize labeling consistency at the class level, we quantified pairwise inter-rater agreement among cardiologists for each CODE diagnostic class (Supplementary Table 16). For a given class, we computed agreement for that class on the shared exams of each cardiologist pair (i.e., exams reviewed by both cardiologists), restricting to pairs with at least 30 shared exams to ensure stable estimates and reduce noise from very small overlaps. For each eligible pair, we computed the observed agreement (proportion of matching binary annotations for that class) and Cohen's kappa, and summarized these quantities across pairs as median [IQR]. Kappa is undefined for degenerate pairs with no label variability on the shared exams (all-positive or all-negative); such pairs are excluded from the kappa summary but retained in the shared-exam counts and observed-agreement summaries, as they still reflect overlap and concordance structure in the audit process. Overall, class-wise agreement is high: 50/66 (75.8%) classes achieve median kappa ≥ 0.90, 10/66 (15.1%) fall in $[0.80, 0.90)$, and 6/66 (9.1%) fall in $[0.70, 0.80)$; no class has median kappa < 0.70. Notably, 40/66 (60.6%) classes exhibit median kappa $= 1.000$. Observed agreement is consistently high across classes, whereas kappa spans a wider range—particularly for rarer and highly imbalanced labels—highlighting residual uncertainty after accounting for marginal labeling tendencies under class imbalance. For example, AXIND (prevalence 0.35%; 8 cardiologists) shows near-perfect observed agreement (0.995 [0.986, 1.000]) but a lower median kappa (0.701 [0.438, 0.904]), consistent with settings where raw agreement can be dominated by concordant negatives. Similar behavior is seen for RAD and ILBBB, which exhibit observed agreement of 1.000 [0.991, 1.000] and 1.000 [0.997, 1.000], respectively, yet lower median kappa (0.748 and 0.766) alongside a non-trivial number of degenerate pairs (RAD: 14; ILBBB: 24). By contrast, several common abnormalities show both high observed agreement and near-perfect kappa (e.g., PAC, RBBB, 1AVB, PVC, and LQT; median kappa = 1.000), although the IQR may extend below 1.000 (e.g., PAC: 1.000 [0.848, 1.000]), reflecting expected

heterogeneity across reviewer pairs. Taken together, the high observed agreement and predominantly high Cohen's kappa values indicate strong inter-rater reliability, with remaining variability concentrated in rare and highly imbalanced classes. These findings support the use of CODE-II-test as a robust expert-adjudicated benchmark for model evaluation.

Cardiologist performance was computed by comparing each individual review with the adjudicated label set of the corresponding exam (agreement rule or label-wise $\geq$ 2-vote majority). Because the reference labels are derived from the same pool of certified cardiologists, these metrics quantify agreement with the adjudicated benchmark (inter-reader consistency under the CODE framework), rather than diagnostic accuracy against an independent external gold standard. Under a fair-scope protocol, we evaluated only diagnostic classes that a cardiologist effectively encountered: for a given cardiologist, classes never assigned by that cardiologist and never present in the adjudicated labels among the exams they reviewed were excluded from per-class and aggregate computations. We derived TP/FP/FN/TN per class and summarized cardiologist-level performance using micro- and macro-averaged precision, recall (sensitivity), specificity, F1-score, NPV, and related metrics (Supplementary Table 8).

Supplementary Table 8 summarizes cardiologist-level performance under the fair-scope definition. Because very small workloads can inflate—sometimes perfect—scores, cardiologists with few exams should be interpreted with caution; we therefore highlight those with $\geq$ 100 reviewed exams (n = 20 cardiologists). Among these cardiologists, the mean micro-F1 score was 0.966 (mean micro-recall 0.978; mean micro-precision 0.956), and the mean macro-F1 score was 0.944, with macro-precision and recall likewise high. Specificity and NPV were near-perfect across cardiologists, which is expected given the exam-level sparsity of labels: most exams carry only one or two diagnostic labels, and multiple labels per exam are relatively uncommon, so for any given class the majority of exam-class pairs are true negatives. The adjudication procedure (complete agreement on the full label set, or otherwise a label-wise majority vote with a $\geq$ 2-vote threshold) guarantees at least one positive label per exam while leaving most other classes negative, reinforcing high specificity and NPV. This adjudication aligns the final ground-truth labels with cardiologist agreement and therefore helps explain the uniformly strong performance observed among high-volume annotators.

C.3 Model Performance Evaluation

This subsection compiles the full evaluation tables for the baseline model on the CODE-II-test set and provides brief context to interpret them. Detailed interpretation appears in the main manuscript. We report both threshold-independent metrics—Area Under the Receiver Operating Characteristic Curve (AUROC) and Area Under the Precision–Recall Curve (AUPRC, also referred to as Average Precision, AP)—and threshold-dependent metrics—precision, recall (sensitivity), specificity, F1-score, and NPV. Our *primary* thresholding protocol uses class-specific F1-max thresholds selected on the CODE-II validation split and applied unchanged to CODE-II-test; unless otherwise noted, all threshold-dependent results in this Supplementary section follow this protocol.

At the aggregate level, the model shows strong discriminative ability (micro-AUROC 0.983, micro-AUPRC 0.776; macro-AUROC 0.978, macro-AUPRC 0.552), with consistently high performance in common classes and greater variability across rare classes. With F1-max thresholds, the model attains micro-F1 0.706 (precision 0.632, recall 0.801) and macro-F1 0.512 (precision 0.517, recall 0.562), reflecting the expected gap when all classes—common and rare—are weighted equally. Stratified analyses by sex, age group, and state (Minas Gerais [MG] vs non-MG) yielded similarly strong discrimination across subgroups, with micro-AUROC ranging from 0.976 to 0.989 and micro-AUPRC from 0.727 to 0.817, and stable macro-averaged performance (macro-AUROC 0.970–0.983; macro-AUPRC 0.551–0.573) (Supplementary Table 17). Confidence intervals largely overlap across strata, supporting the robustness of threshold-independent performance in these subgroup slices.

To complement discrimination metrics and to better interpret the model's probabilistic outputs, we additionally assessed calibration on CODE-II-test using two standard measures: Expected Calibration Error (ECE) and the Brier score (lower values indicate better calibration). Both metrics were computed directly from the raw predicted probabilities (without any post-hoc recalibration). We report macro- and micro-averaged ECE and Brier scores with 95% confidence intervals estimated from 1,000 exam-level bootstrap resamples (Supplementary Table 19). Overall, calibration was strong at the aggregate level (micro-ECE 0.0022 and macro-ECE 0.0043; micro/macro Brier score 0.0087). We further report per-class results for all 66 CODE diagnostic classes (Supplementary Table 18), with ECE ranging from 0.0002 to 0.0259 and Brier score from 0.0002 to 0.0788; absolute Brier values are influenced by class prevalence and therefore are not directly comparable across classes. To provide an intuitive visual summary, Supplementary

Fig. 29 shows reliability diagrams for three representative classes (NORMAL, LBBB, and AF), alongside a panel summarizing each class's prevalence, ECE, and Brier score. Importantly, these analyses characterize calibration in this benchmark setting and should not be interpreted as deployment-ready optimization; a systematic evaluation of post-hoc calibration strategies and clinically defined operating points under distribution shift is outside the scope of this work.

Per-class results illustrate both common and rare diagnoses. For example, the Normal class (40.1% of CODE-II-test) achieves AUPRC 0.931 (well above prevalence 0.401), while clinically important abnormalities such as LBBB (2.6%) and AF (2.6%) reach AUPRC 0.944 and 0.950, respectively. Even in rare but time-critical conditions such as STEMI (0.4%), the model attains AUPRC 0.647, substantially above prevalence (see Supplementary Table 9).

Because threshold choice can materially affect threshold-dependent metrics when validation and test distributions differ in class prevalence and score calibration, we also provide a *sensitivity analysis* comparing the primary F1-max thresholds with an alternative rule based on Youden's J statistic ($J =$ sensitivity + specificity − 1), with both sets of thresholds selected on validation and applied unchanged to CODE-II-test. As expected, AUROC/AUPRC remain unchanged, whereas precision/recall/F1 shift notably; these micro/macro comparisons are reported in Supplementary Table 10.

At the class level, three rare diagnoses—multifocal atrial tachycardia (MAT), isorhythmic atrioventricular dissociation (IAVD), and digitalis effect (DIG)—yield degenerate metrics under the F1-max rule on the Test set (precision, recall, and F1 equal to zero). For these classes, Youden's J selects substantially smaller thresholds, permitting positive predictions and non-zero precision/recall/F1. Side-by-side results are reported in Supplementary Table 11.

As brief context relative to specialists, the previous subsection ("Cardiologist Performance Evaluation") reports uniformly high reviewer performance under a fair-scope protocol (see Supplementary Table 8), which is expected because the CODE-II-test ground-truth was adjudicated by agreement/majority rules—thereby aligning reference labels with cardiologist concordance and yielding near-perfect specificity/NPV at the exam-class level. By contrast, the model's threshold-dependent metrics reflect class-specific thresholds selected on the validation split and applied unchanged to Test, which differs in class prevalence and score calibration; this depresses precision/recall/F1 in some settings, as shown by the Youden's-J sensitivity analysis (Supplementary Table 10) and by the three rare classes with degenerate values under F1-max (Supplementary Table 11). A formal head-to-head comparison is therefore beyond scope here; in future work we will examine threshold-selection and calibration strategies under distribution shift, alongside clinically motivated operating modes tailored to specific use cases.

D External Evaluation and Model Benchmarking

This supplementary section provides additional details on the external evaluation experiments described in the main manuscript. We assessed the generalization capabilities of the model pre-trained on the CODE-II dataset by fine-tuning it on two widely used public ECG classification benchmarks: PTB-XL and CPSC 2018. These datasets differ from CODE-II in terms of patient population, diagnostic labeling schemes, and acquisition settings, thus offering a robust framework for evaluating the transferability of the learned representations. We conducted experiments under both full-data and few-shot training scenarios (using 5% and 10% of the training data) and compared our model against supervised models trained from scratch as well as state-of-the-art pre-trained ECG models. The results presented in the following figures and tables demonstrate how the combination of a high-quality, expertly curated dataset and a robust model architecture can yield highly transferable and diagnostically meaningful representations across diverse clinical scenarios.

D.1 General Characteristics

Detailed performance results for all evaluated models on PTB-XL and CPSC 2018 under full-data training are summarized in Supplementary Table 12, illustrating differences across model families, including end-to-end supervised baselines and pre-trained approaches. The complete set of results—including additional few-shot training experiments (5% and 10%) on PTB-XL, improvements over random initialization, and per-run values—is provided in Supplementary Data 6 (external Excel file, *Supplementary_Data_6.xlsx*).

Supplementary Table 13 summarizes the number of parameters, as well as the size and source of the pre-training datasets used for each model included in the benchmarking. These details help contextualize the representational capacity of the models and the scale of data involved in their original training, which may influence downstream performance in external evaluation tasks.

Details about the fine-tuning configurations used for each pre-trained model—including learning rates, input signal properties, and fine-tuning strategies—are provided in Supplementary Table 14. These parameters were selected to ensure comparability while accommodating model-specific requirements and published recommendations.

E Benchmarking on CODE-II-open

This supplementary section provides additional details on the benchmarking experiments performed using the CODE-II-open dataset, as described in the main manuscript. The purpose of this analysis was to illustrate the representativeness of CODE-II-open as a benchmark dataset and to compare the performance of our baseline architecture with reference ECG models under consistent evaluation settings. The same evaluation pipeline adopted for the external benchmarking experiments was used here to ensure comparability across studies.

E.1 General Characteristics

Supplementary Table 15 reports the results of our baseline model and of models from the literature under their best-performing regime on CODE-II-open, either trained from scratch or fine-tuned from pre-trained versions. For reference, the table also includes the performance of our baseline model when trained on the full CODE-II dataset. These results demonstrate that the CODE-II-open dataset is sufficiently rich and diverse to train competitive ECG classifiers and support its role as a public benchmark for future model development.

F List of Supplementary Figures

Supplementary Fig. 1: Number of ECG exams per year in the CODE-II dataset.

Supplementary Fig. 2: Age distribution of patients at their first ECG exam in the CODE-II dataset, stratified by sex.

Supplementary Fig. 3: Clinical indications for exams, stratified by age group and sex, in the CODE-II dataset.

Supplementary Fig. 4: Summary of exam and signal record distributions in the CODE-II dataset. (a) Distribution of the number of ECG exams per patient. (b) Distribution of the number of signal records per exam. Each exam may include multiple consecutive tracings recorded during the same clinical session.

Supplementary Fig. 5: Final distribution of the number of tracings per exam in the CODE-II dataset after applying the pipeline that retains up to four tracings per exam.

Supplementary Fig. 6: Analysis of group-level diagnostic label assignments in the CODE-II dataset. (a) Number of exams exclusively assigned to each of the 10 CODE groups. (b) Ten most frequent non-exclusive group combinations, among 251 distinct combinations observed. Group names were abbreviated (using the first four letters or a combination of initials) and joined by "+" to denote co-occurrence. (c) Distribution of the number of CODE groups assigned per exam, indicating how frequently exams belong to multiple groups.

Supplementary Fig. 7: Median and interquartile range (IQR) of cardiac measurements stratified by age group and sex in the CODE-II dataset. (a) Heart rate (beats per minute); (b) P wave duration (milliseconds), reflecting atrial depolarization; (c) PR interval (ms), reflecting atrioventricular conduction time; (d) QRS duration (ms), representing ventricular depolarization; (e) QRS axis (degrees), showing the mean frontal plane electrical axis; and (f) Corrected QT interval (QTc, ms), adjusted for heart rate.

Supplementary Fig. 8: Number of ECG exams per year in the CODE-II-open dataset.

Supplementary Fig. 9: Geographic distribution of ECG exams in the CODE-II-open dataset across Brazilian states.

Supplementary Fig. 10: Age distribution of patients in the CODE-II-open dataset, stratified by sex.

Supplementary Fig. 11: Distribution of ECG exam indications in the CODE-II-open dataset by age group and sex.

Supplementary Fig. 12: Distribution of the number of signal records per exam in the CODE-II-open dataset. Each exam may include multiple consecutive tracings recorded during the same clinical session.

Supplementary Fig. 13: Final distribution of the number of tracings per exam in the CODE-II-open dataset after applying the pipeline that retains up to four tracings per exam.

Supplementary Fig. 14: Number of exams per diagnostic class in the CODE-II-open dataset. Class assignments are not mutually exclusive: a single exam may be associated with multiple diagnoses, except for Normal ECGs, which are assigned exclusively.

Supplementary Fig. 15: Distribution of CODE-II-open ECG exams by diagnostic group and number of assigned diagnoses. (a) Number of exams per CODE diagnostic group (note that exams may appear in multiple groups), and (b) the number of exams with a given number of assigned CODE diagnoses.

Supplementary Fig. 16: Analysis of group-level diagnostic label assignments in the CODE-II-open dataset. (a) Number of exams exclusively assigned to each of the 10 CODE groups. (b) Ten most frequent non-exclusive group combinations, among 147 distinct combinations observed. Group names were abbreviated (using the first four letters or a combination of initials) and joined by "+" to denote co-occurrence. (c) Distribution of the number of CODE groups assigned per exam, indicating how frequently exams belong to multiple groups.

Supplementary Fig. 17: Median and interquartile range (IQR) of electrocardiographic measurements in the CODE-II-open dataset, stratified by age group and sex. (a) Heart rate (beats per minute); (b) P wave duration (milliseconds), reflecting atrial depolarization; (c) PR interval (ms), reflecting atrioventricular conduction time; (d) QRS duration (ms), representing ventricular depolarization; (e) QRS axis (degrees), showing the mean frontal plane electrical axis; and (f) Corrected QT interval (QTc, ms), adjusted for heart rate.

Supplementary Fig. 18: Annual distribution of ECG exams in the CODE-II-test dataset (2018–2025).

Supplementary Fig. 19: Geographic distribution of ECG exams in the CODE-II-test dataset across Brazilian states.

Supplementary Fig. 20: Age distribution of patients in the CODE-II-test dataset, stratified by sex.

Supplementary Fig. 21: Distribution of ECG exam indications in the CODE-II-test dataset by age group and sex.

Supplementary Fig. 22: Number of signal records per exam in the CODE-II-test dataset.

Supplementary Fig. 23: Final distribution of the number of tracings per exam in the CODE-II-test dataset after applying the pipeline that retains up to four tracings per exam.

Supplementary Fig. 24: Distribution of ECG exams per diagnostic class in the CODE-II-test dataset. Class assignments are not mutually exclusive: a single exam may be associated with multiple diagnoses, except for Normal ECGs, which are assigned exclusively.

Supplementary Fig. 25: Distribution of ECG exams in the CODE-II-test dataset by diagnostic group and by number of assigned diagnoses. (a) Number of exams per CODE diagnostic group (exams may appear in multiple groups). (b) Distribution of exams by number of assigned CODE diagnoses.

Supplementary Fig. 26: Group-level diagnostic label assignments in the CODE-II-test dataset. (a) Number of exams exclusively assigned to each of the 10 CODE groups. (b) Ten most frequent non-exclusive group combinations, among 106 distinct combinations observed. Group names were abbreviated (using the first four letters or a combination of initials) and joined by "+" to denote co-occurrence. (c) Distribution of the number of CODE groups assigned per exam, indicating how frequently exams belong to multiple groups.

Supplementary Fig. 27: Distribution of the number of exams annotated per cardiologist in the CODE-II-test dataset (n = 46 cardiologists).

Supplementary Fig. 28: Distribution of the number of cardiologists per exam in the CODE-II-test dataset.

Supplementary Fig. 29: Calibration curves for three representative diagnostic classes in the CODE-II-test dataset. Panels (a–c) show reliability diagrams for NORMAL, LBBB, and AF, respectively, plotting observed frequency against predicted probability. The dashed diagonal line indicates perfect calibration, while the solid line represents the empirical calibration curve obtained by binning predicted probabilities. Panel (d) summarizes, for these classes, the test-set prevalence, expected calibration error (ECE), and Brier score.

G List of Supplementary Tables

Supplementary Table 1: Self-reported clinical comorbidities of patients in the CODE-II dataset, stratified by age group (18–59 years and $\geq$ 60 years) and sex, based on the first ECG exam. For hypertesion, dyslipidemia, and diabetes, comorbidity status also inferred from reported medication usage.

Supplementary Table 2: Summary of the 66 CODE diagnostic classes in the CODE-II dataset, including class descriptions, labels, and the number and percentage of exams in the full dataset (n = 2,735,269), as well as class-wise counts of unique patients and ECG exams in the training and validation sets obtained using the proposed patient-exclusive stratified split. The complete table is also provided as Supplementary Data 1 (external Excel file, *Supplementary_Data_1.xlsx*).

Supplementary Table 3: Group-level distribution of CODE diagnostic assignments at the patient level, stratified by age (18–59 and $\geq$ 60 years) and sex. Columns report counts by stratum, totals, the percentage of patients within each diagnosis group (by sex and overall), and the percentage relative to all patients in CODE-II. Diagnostic groups are not mutually exclusive; patients may belong to multiple groups, except for the Normal group, which is exclusive by definition.

Supplementary Table 4: Prevalence of self-reported or inferred comorbidities across age and sex groups in the CODE-II-open dataset.

Supplementary Table 5: Summary of the 66 CODE diagnostic classes in the CODE-II-open dataset, including class descriptions, labels, and the number and percentage of exams in the dataset (n = 15,000), as well as class-wise counts of unique patients and ECG exams in the training and validation sets obtained using the proposed patient-exclusive stratified split. The complete table is also provided as Supplementary Data 2 (external Excel file, *Supplementary_Data_2.xlsx*).

Supplementary Table 6: Prevalence of self-reported or inferred comorbidities across age and sex groups in the CODE-II-test dataset.

Supplementary Table 7: Summary of the 66 CODE diagnostic classes in the CODE-II-test dataset, including class descriptions, labels, and the number and percentage of exams in the dataset (n = 8,475). The complete table is also provided as Supplementary Data 3 (external Excel file, *Supplementary_Data_3.xlsx*).

Supplementary Table 8: Cardiologist-level performance on the CODE-II-test under the fair-scope definition. For each cardiologist, we report the number of exams reviewed, the number of diagnostic classes encountered, and micro- and macro-averaged precision, recall, specificity, F1 score, and NPV computed only over classes actually seen by that cardiologist. To avoid small-sample inflation, the table lists cardiologists with $\geq$ 100 reviewed exams. The complete list, including all cardiologists, is provided in Supplementary Data 4 (external Excel file, Supplementary_Data_4.xlsx).

Supplementary Table 9: Per-class model performance on the CODE-II-test under the F1-max thresholding rule. For each diagnostic class, we report test prevalence (number and percentage), threshold-independent metrics (AUROC, AUPRC), and threshold-dependent metrics (F1-score, Recall/Sensitivity, Specificity, Precision, and NPV) computed using class-specific thresholds selected on the CODE-II validation split and applied unchanged to the test set. All metrics are reported with 95% confidence intervals estimated from 1,000 bootstrap resamples (shown in parentheses). The complete table is also provided as Supplementary Data 5 (external Excel file, *Supplementary_Data_5.xlsx*).

Supplementary Table 10: Aggregate model performance on the CODE-II-test under two threshold rules: the primary F1-max thresholds and an alternative based on Youden's J (both selected on the CODE-II validation split and applied unchanged to the Test set). Micro- and macro-averaged metrics are reported—Precision, Recall, F1-score, AUROC, and AUPRC—for each rule.

Supplementary Table 11: Per-class model performance on the CODE-II-test for the three diagnostic classes that produced degenerate metrics under the F1-max rule (precision, recall, and F1 equal to zero). For each class, we report Test-set prevalence (number and %), AUROC and AUPRC, and—under two thresholding rules, F1-max and Youden's J, both selected on the CODE-II validation split and applied unchanged to the Test set—the selected threshold and the resulting Precision, Recall, Specificity, F1-score, and NPV.

Supplementary Table 12: Macro-AUROC results for all supervised and pre-trained models on the five PTB-XL tasks—diagnosis, subclass, superclass, form, and rhythm—and on the full CPSC 2018 dataset. All models were trained using 100% of the available training data. For each model, we report the mean macro-AUROC over three runs, with the run-to-run variation shown in parentheses (maximum minus minimum). The table also includes the average macro-AUROC across tasks and the total number of model parameters. The complete set of results, including each model under few-shot training (5% and 10%) on PTB-XL and the improvement relative to its randomly initialized counterpart (when applicable), is provided in Supplementary Data 6 (external Excel file, *Supplementary_Data_6.xlsx*).

Supplementary Table 13: Model sizes and pre-training datasets for baseline ECG models. The table reports the number of parameters (estimated from full-data training on PTB-XL diagnostic classes), the size of the pre-training set (in ECG records or ECG–report pairs), and the corresponding data sources used for model pre-training.

Supplementary Table 14: Training and fine-tuning configurations for pre-trained ECG models. For each model, we report the learning rates used under full-data and few-shot scenarios, the input frequency and segment length, and the adopted fine-tuning strategy. When applicable, model-specific exceptions are noted. The same configurations were used for both PTB-XL and CPSC 2018 experiments unless otherwise specified. Input signals shorter than the target input length were symmetrically zero-padded, while longer signals were symmetrically truncated.

Supplementary Table 15: Performance of the baseline and literature ECG models on the CODE-II-open dataset under their best-performing configuration, either trained from scratch or fine-tuned from pre-trained versions. The same evaluation pipeline used for external benchmarking was applied to ensure comparability across experiments. Metrics are reported as macro–AUROC and macro–average precision (macro–AP). The performance of the baseline model trained on the full CODE-II cohort is shown for reference.

Supplementary Table 16: Per-class inter-rater agreement among cardiologists (pairwise). For each CODE diagnostic class, we report exam-level prevalence and cardiologist coverage, including the number of cardiologists who reviewed the class and the number of cardiologist pairs with defined or undefined Cohen's kappa. For each cardiologist pair, agreement is computed on their shared exams (including only pairs with $\geq$30 shared exams) and summarized across pairs as median [IQR] for the number of shared exams and observed agreement. Cohen's kappa is summarized only over non-degenerate pairs (i.e., excluding pairs whose shared-exam annotations are single-label: all-positive or all-negative), whereas these degenerate pairs are retained in the shared-exam and observed agreement summaries. Exams reviewed by more than two cardiologists contribute to multiple pairwise comparisons (one per cardiologist pair). All 66 classes are shown and ordered by median Cohen's kappa (descending).

Supplementary Table 17: Stratified model performance on the CODE-II-test. Results are reported by sex, age group, state (Minas Gerais [MG] vs non-MG, where non-MG includes all other Brazilian states represented in CODE-II-test), and comorbidity burden (defined as the presence of zero or at least one of seven pre-specified clinical conditions). For each subgroup, we report the number of exams and its proportion within the corresponding stratum, along with micro- and macro-averaged AUROC and AUPRC. Values are shown as point estimates with 95% confidence intervals (in parentheses) estimated from 1,000 bootstrap resamples at the exam level.

Supplementary Table 18: Per-class calibration results on the CODE-II-test. For each of the 66 CODE diagnostic classes, we report test prevalence (number of exams and percentage) and two calibration

metrics: Expected Calibration Error (ECE) and Brier score. Values are shown as point estimates with 95% confidence intervals (in parentheses) estimated from 1,000 exam-level bootstrap resamples.

Supplementary Table 19: Overall calibration performance on the CODE-II-test. We report macro- and micro-averaged Expected Calibration Error (ECE) and Brier score, with 95% confidence intervals estimated from 1,000 exam-level bootstrap resamples (shown in parentheses).

H List of Supplementary Data (external files)

Supplementary Data 1: Full distribution of the 66 CODE diagnostic classes in the CODE-II dataset, including class descriptions, labels, and the number and percentage of exams in the full dataset (n = 2,735,269), as well as class-wise counts of unique patients and ECG exams in the training and validation sets obtained using the proposed patient-exclusive stratified split. *Data are provided as a separate downloadable Excel file* (File: `Supplementary_Data_1.xlsx`).

Supplementary Data 2: Full distribution of the 66 CODE diagnostic classes in the CODE-II-open dataset, including class descriptions, labels, and the number and percentage of exams in the dataset (n = 15,000), as well as class-wise counts of ECG exams in the training and validation sets obtained using the proposed patient-exclusive stratified split. *Data are provided as a separate downloadable Excel file* (File: `Supplementary_Data_2.xlsx`).

Supplementary Data 3: Full distribution of the 66 CODE diagnostic classes in the CODE-II-test dataset, including class descriptions, labels, and the number and percentage of exams in the dataset (n = 8,475). *Data are provided as a separate downloadable Excel file* (File: `Supplementary_Data_3.xlsx`).

Supplementary Data 4: Complete cardiologist-level performance on the CODE-II-test under the fair-scope definition, including all reviewers (n = 46 cardiologists). Columns include cardiologist ID, exams reviewed, diagnostic classes seen, and micro- and macro-averaged precision, recall, specificity, F1 score, and NPV computed over classes encountered by each cardiologist. *Data are provided as a separate downloadable Excel file* (File: `Supplementary_Data_4.xlsx`).

Supplementary Data 5: Per-class model performance on the CODE-II-test under the F1-max thresholding rule. For all 66 diagnostic classes, we report Test prevalence (number and percentage), threshold-independent metrics (AUROC, AUPRC), and threshold-dependent metrics (F1-score, Recall/Sensitivity, Specificity, Precision, and NPV) computed using class-specific thresholds selected on the CODE-II validation split and applied unchanged to the test set. All metrics are reported with 95% confidence intervals estimated from 1,000 bootstrap resamples (shown in parentheses). *Data are provided as a separate downloadable Excel file* (File: `Supplementary_Data_5.xlsx`).

Supplementary Data 6: Macro-AUROC results for all supervised and pre-trained models on PTB-XL (across the diagnostic, subclass, superclass, form, and rhythm categories) and on CPSC 2018. Both datasets were evaluated under full-data training, while few-shot training (5% and 10%) was applied only to PTB-XL. Reported values are means over three runs, with the run-to-run range width (maximum minus minimum) shown in parentheses. For each model, we also report the average macro-AUROC across tasks, the improvement relative to its randomly initialized counterpart (when applicable), and the total number of model parameters. A second worksheet provides per-run results. *Data are provided as a separate downloadable Excel file* (File: `Supplementary_Data_6.xlsx`).

Supplementary Data 7: Performance of the baseline and reference ECG models on the CODE-II-open dataset under their best-performing configurations, either trained from scratch or fine-tuned from pre-trained weights. The same evaluation pipeline used for external benchmarking was applied to ensure comparability across experiments. Metrics are reported as macro-AUROC and macro-AUPRC for each of the three independent runs, together with their mean and performance range (maximum–minimum) across runs. *Data are provided as a separate downloadable Excel file* (File: `Supplementary_Data_7.xlsx`).

Supplementary Figures

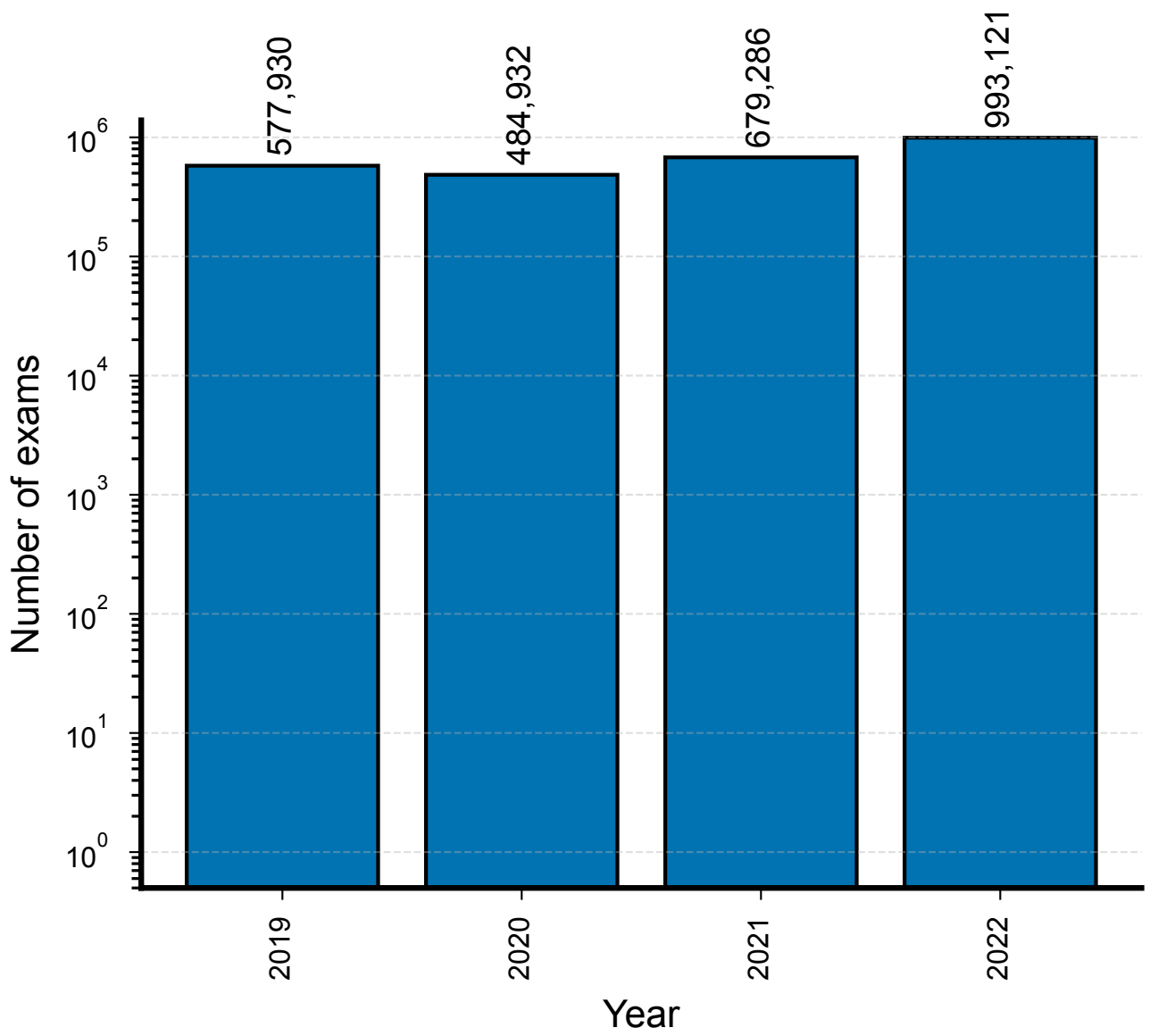

Supplementary Fig. 1. Number of ECG exams per year in the CODE-II dataset.

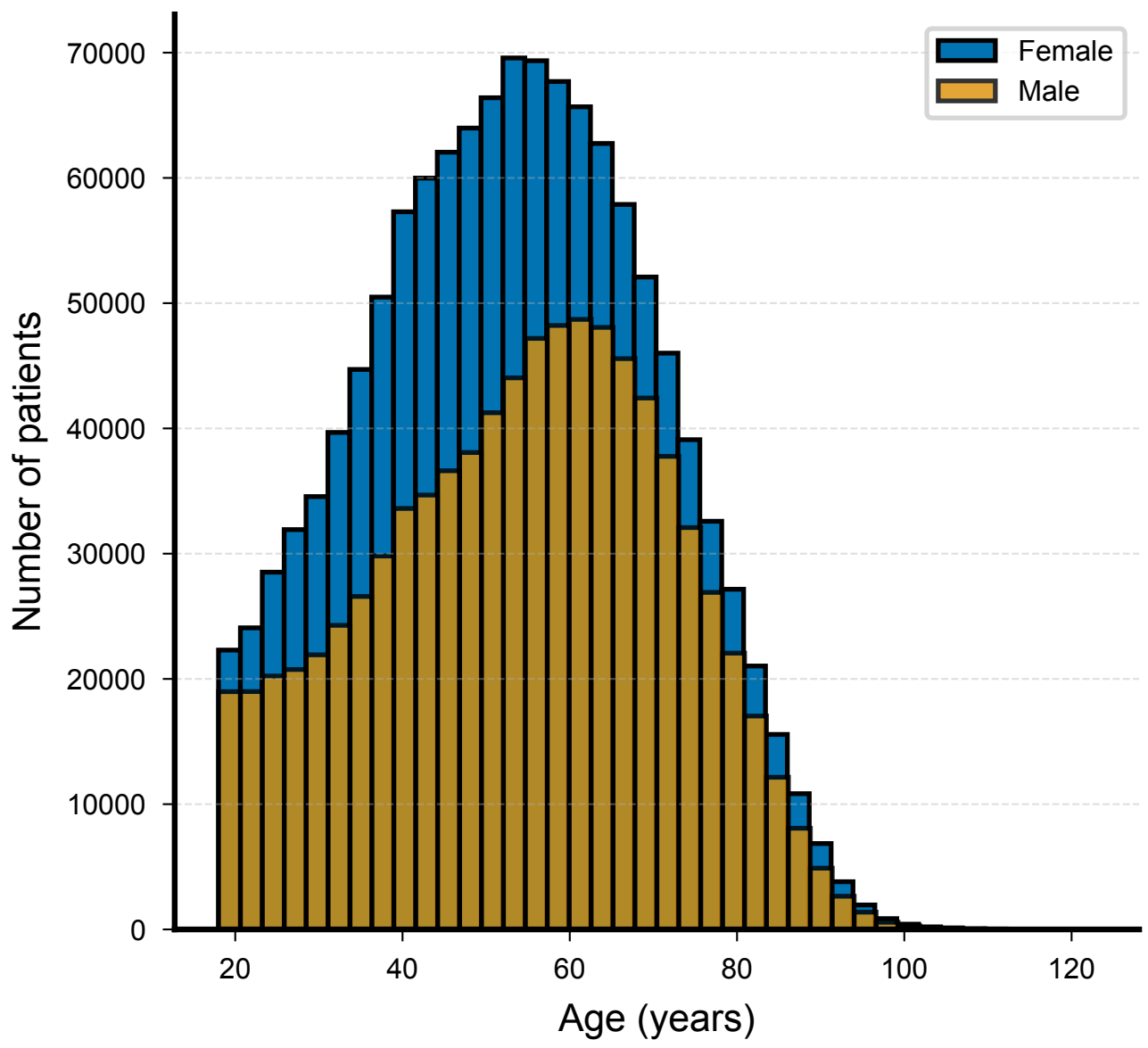

Supplementary Fig. 2. Age distribution of patients at their first ECG exam in the CODE-II dataset, stratified by sex.

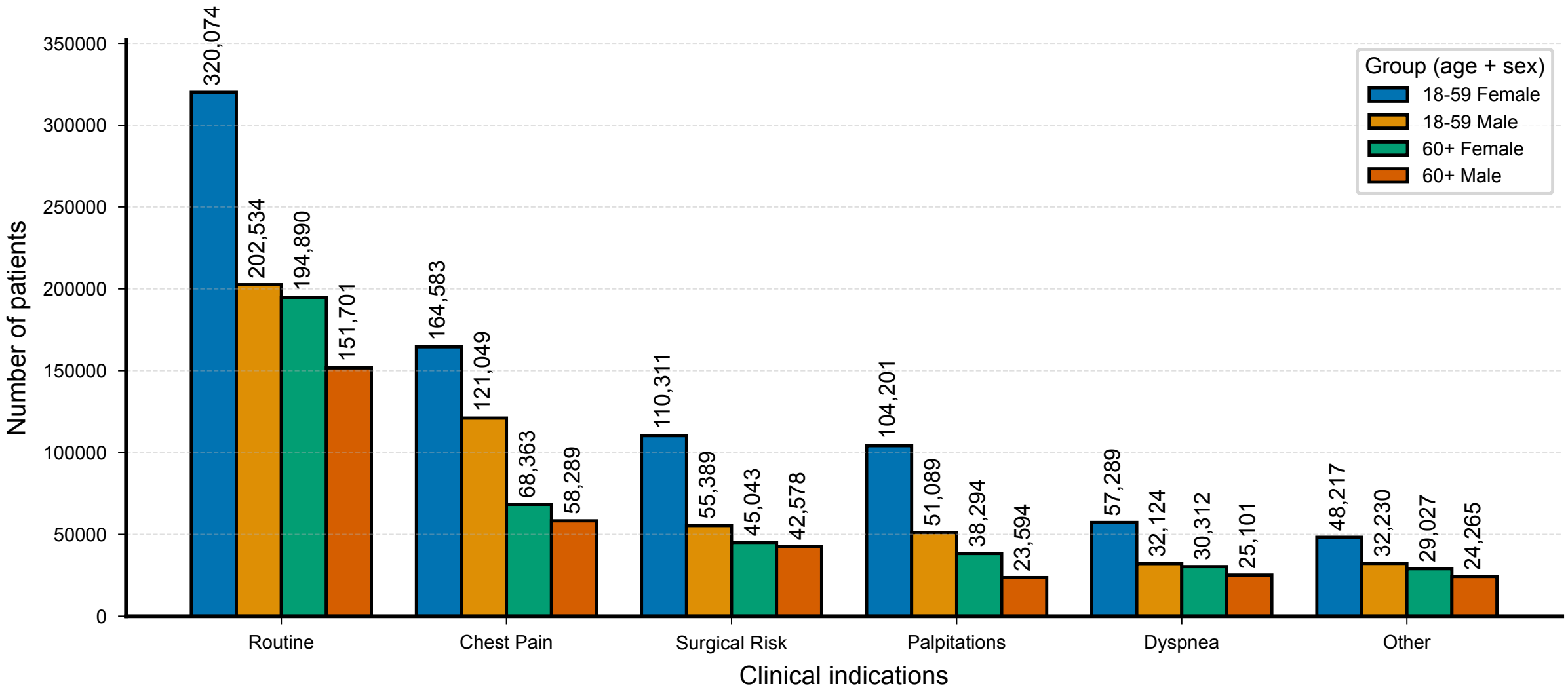

Supplementary Fig. 3. Clinical indications for exams, stratified by age group and sex, in the CODE-II dataset.

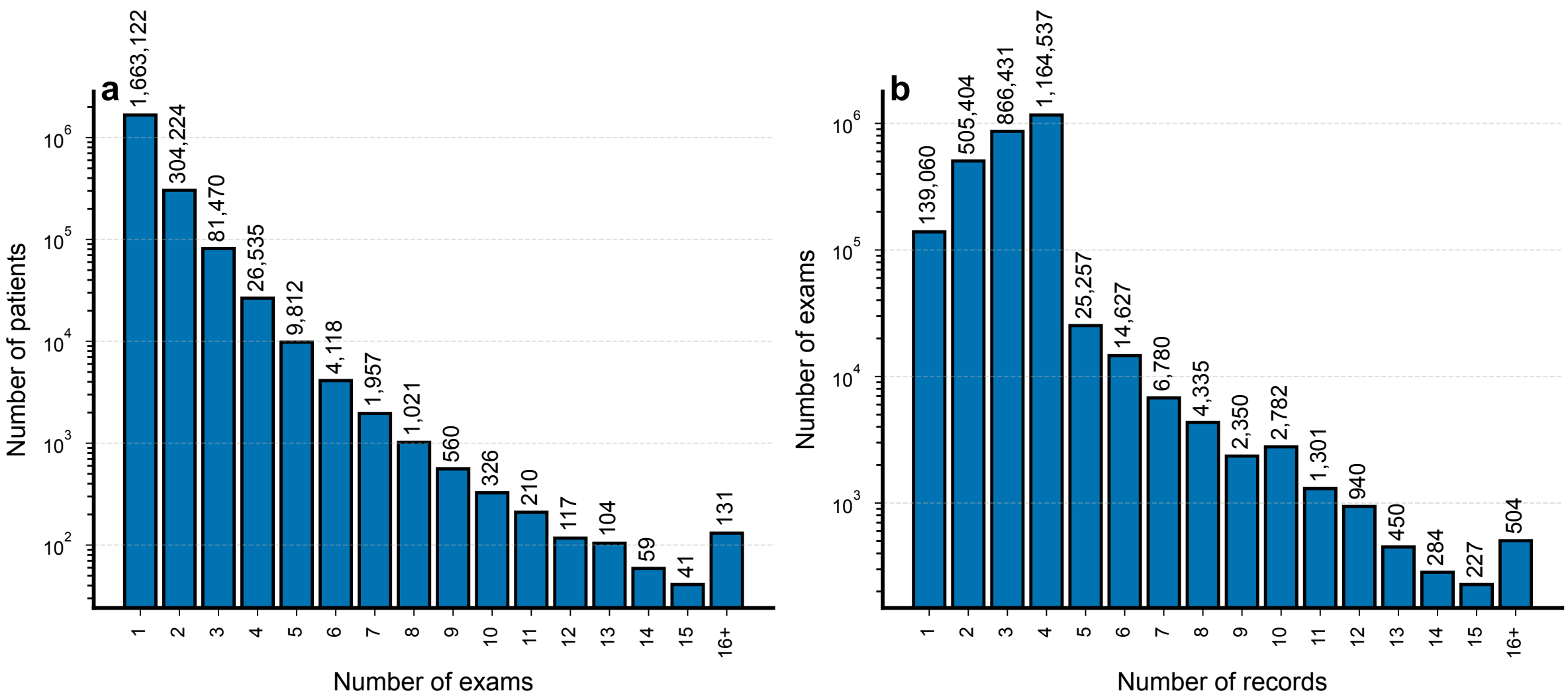

Supplementary Fig. 4. Summary of exam and signal record distributions in the CODE-II dataset. (a) Distribution of the number of ECG exams per patient. (b) Distribution of the number of signal records per exam. Each exam may include multiple consecutive tracings recorded during the same clinical session.

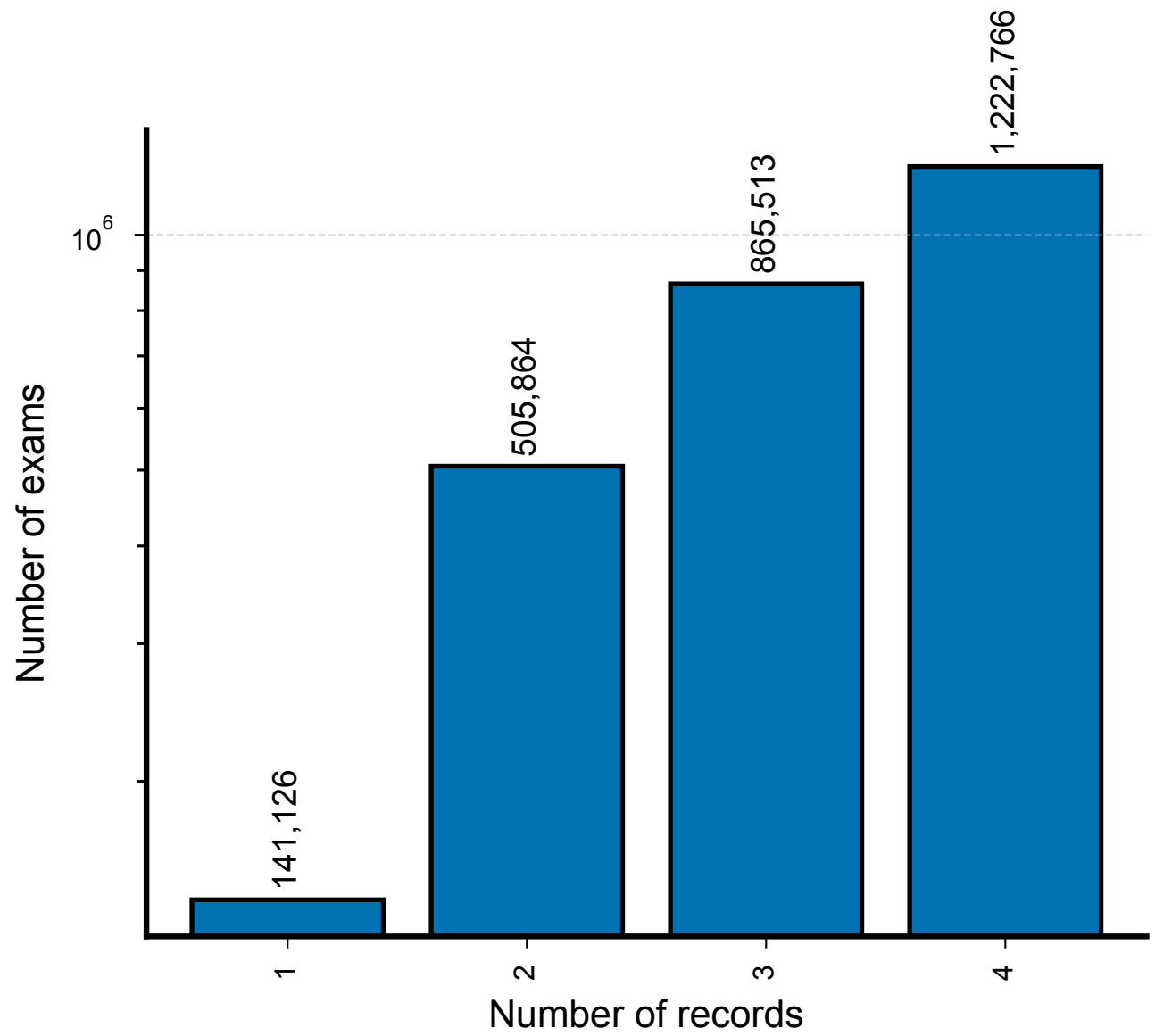

Supplementary Fig. 5. Final distribution of the number of tracings per exam in the CODE-II dataset after applying the pipeline that retains up to four tracings per exam.

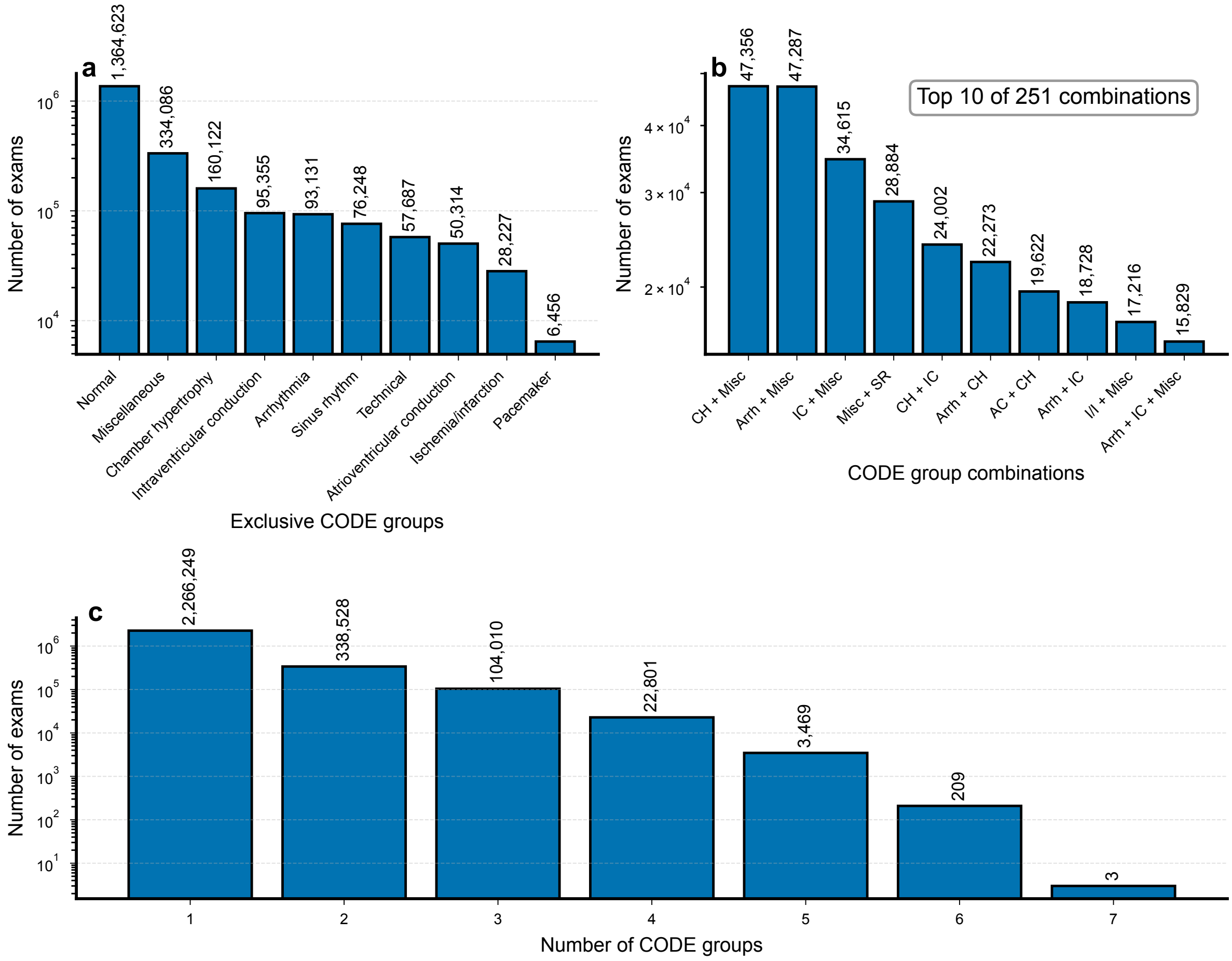

Supplementary Fig. 6. Analysis of group-level diagnostic label assignments in the CODE-II dataset. (a) Number of exams exclusively assigned to each of the 10 CODE groups. (b) Ten most frequent non-exclusive group combinations, among 251 distinct combinations observed. Group names were abbreviated (using the first four letters or a combination of initials) and joined by "+" to denote co-occurrence. (c) Distribution of the number of CODE groups assigned per exam, indicating how frequently exams belong to multiple groups.

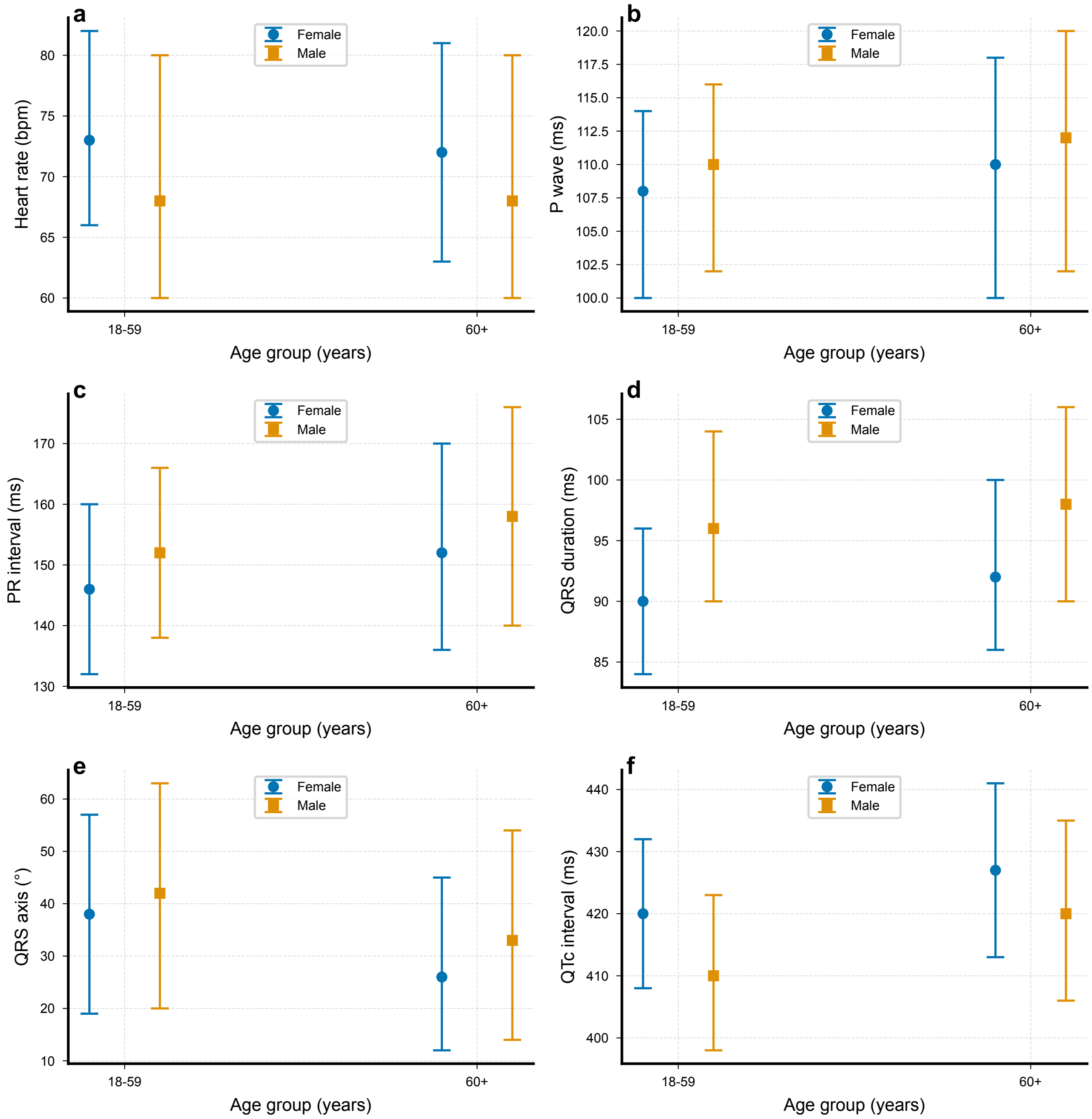

Supplementary Fig. 7. Median and interquartile range (IQR) of cardiac measurements stratified by age group and sex in the CODE-II dataset. (a) Heart rate (beats per minute); (b) P wave duration (milliseconds), reflecting atrial depolarization; (c) PR interval (ms), reflecting atrioventricular conduction time; (d) QRS duration (ms), representing ventricular depolarization; (e) QRS axis (degrees), showing the mean frontal plane electrical axis; and (f) Corrected QT interval (QTc, ms), adjusted for heart rate.

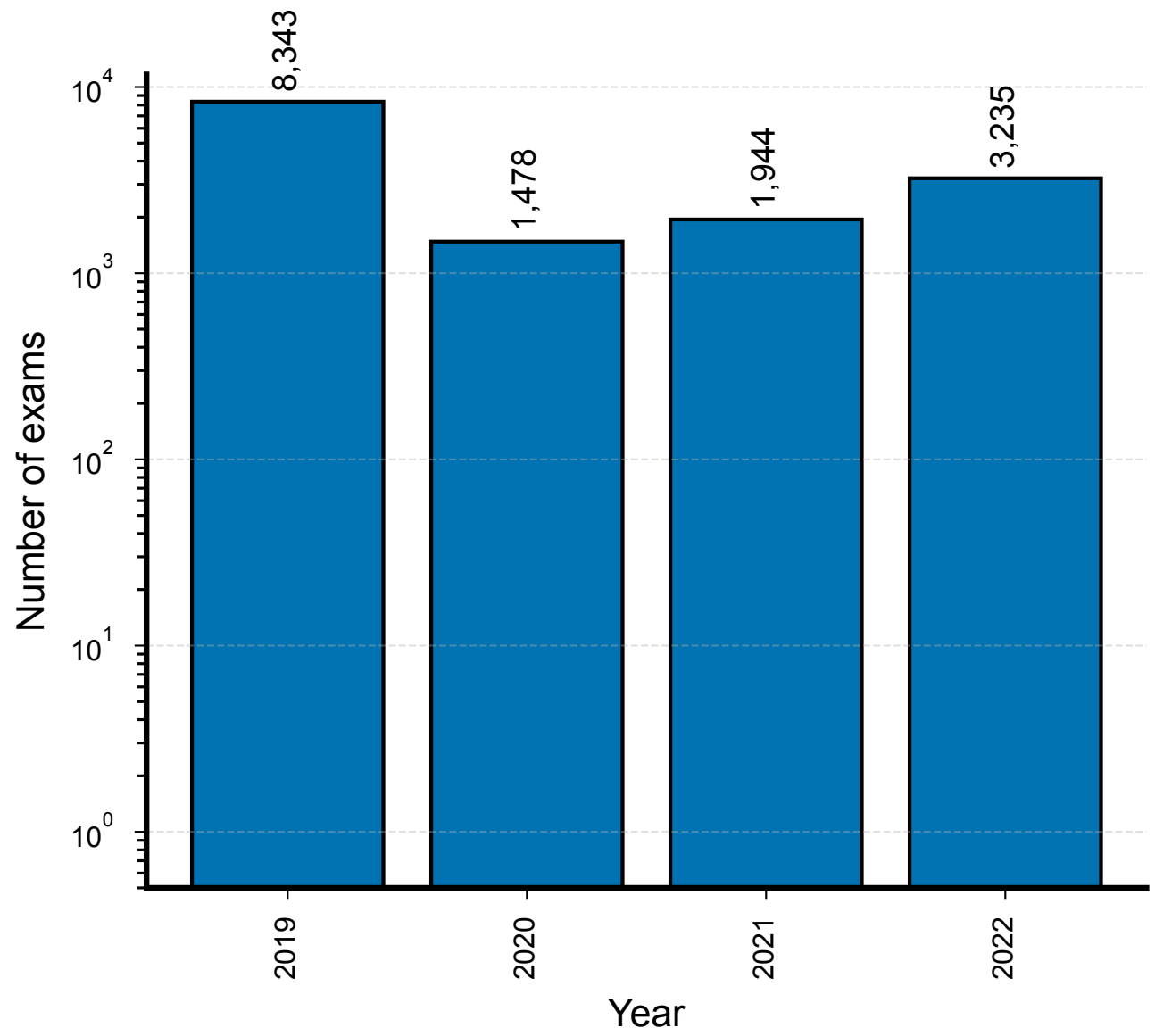

Supplementary Fig. 8. Number of ECG exams per year in the CODE-II-open dataset.

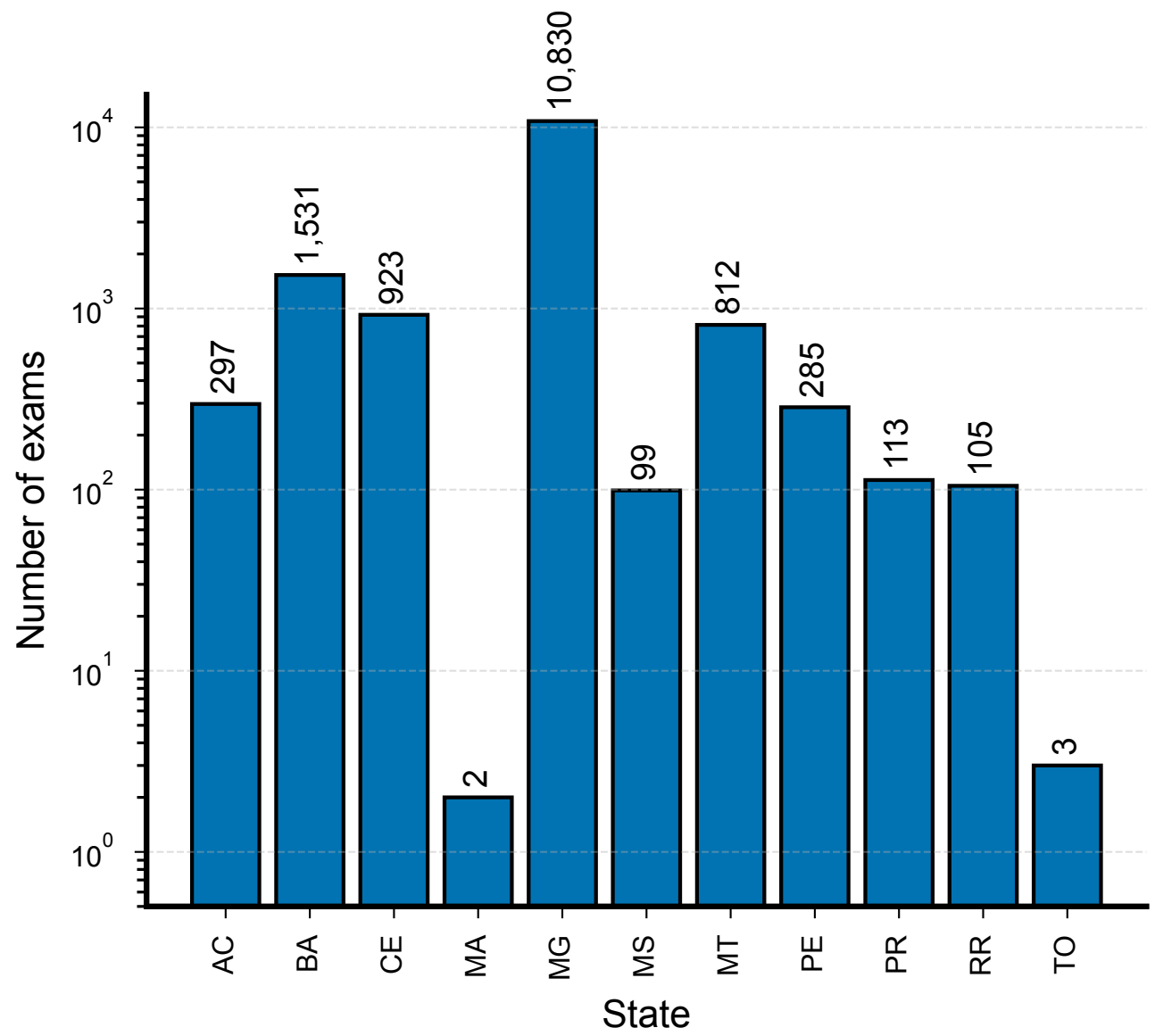

Supplementary Fig. 9. Geographic distribution of ECG exams in the CODE-II-open dataset across Brazilian states.

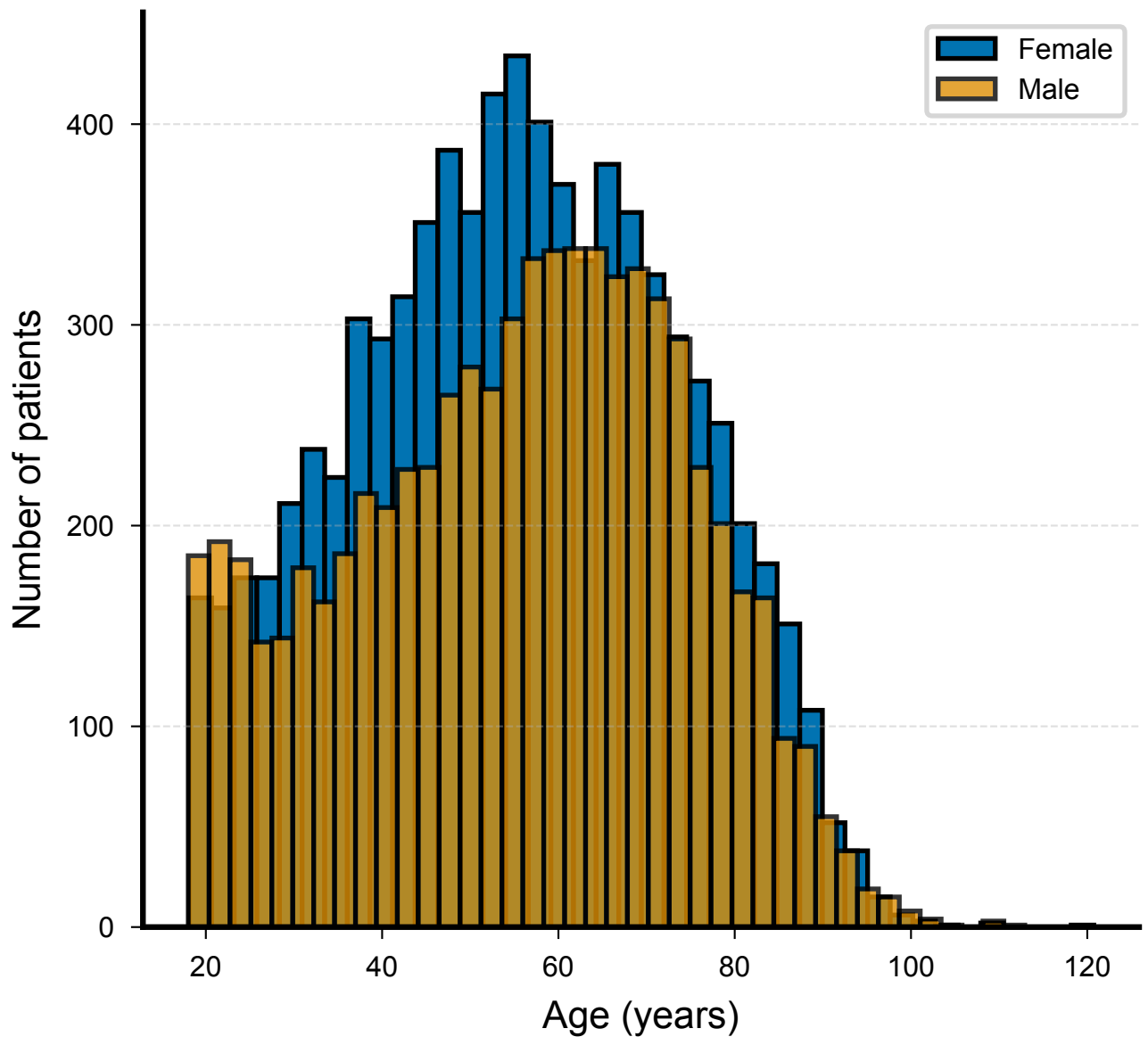

Supplementary Fig. 10. Age distribution of patients in the CODE-II-open dataset, stratified by sex.

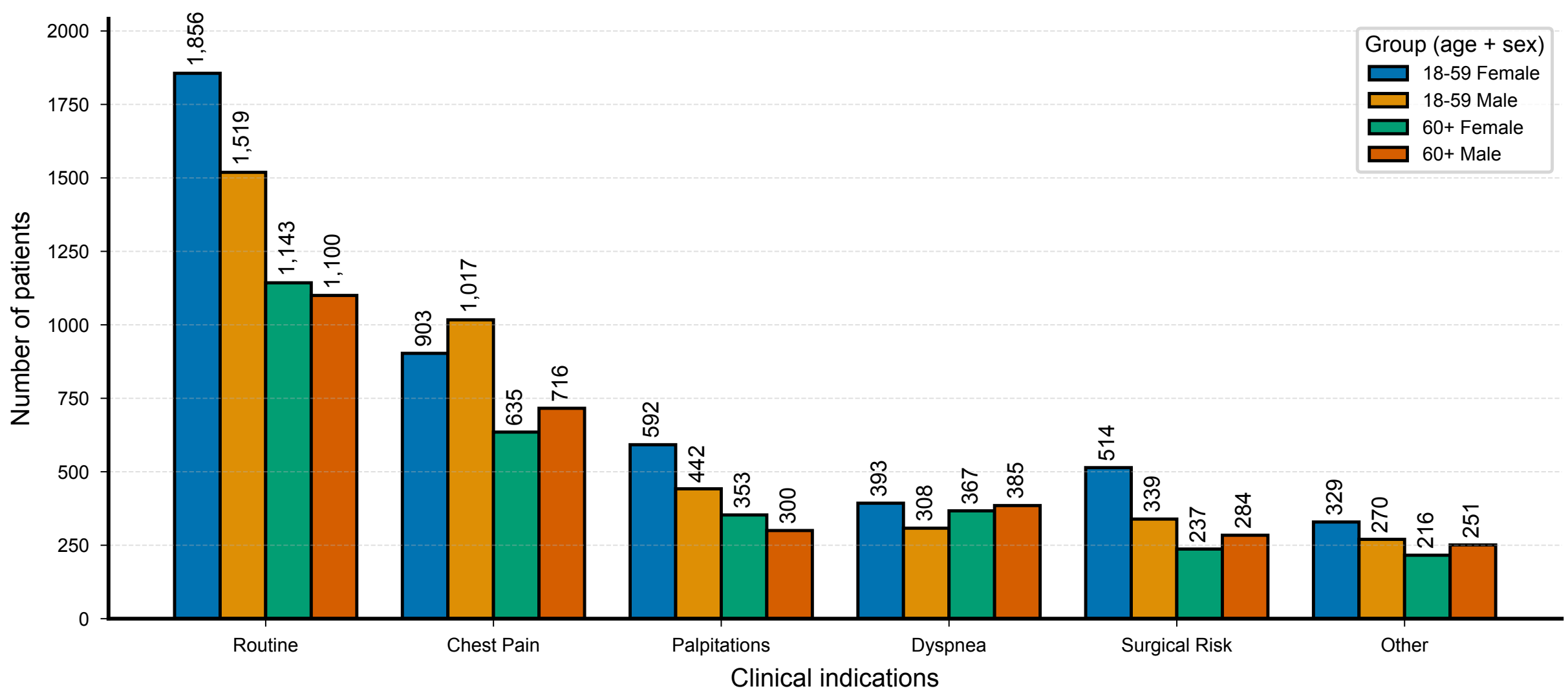

Supplementary Fig. 11. Distribution of ECG exam indications in the CODE-II-open dataset by age group and sex.

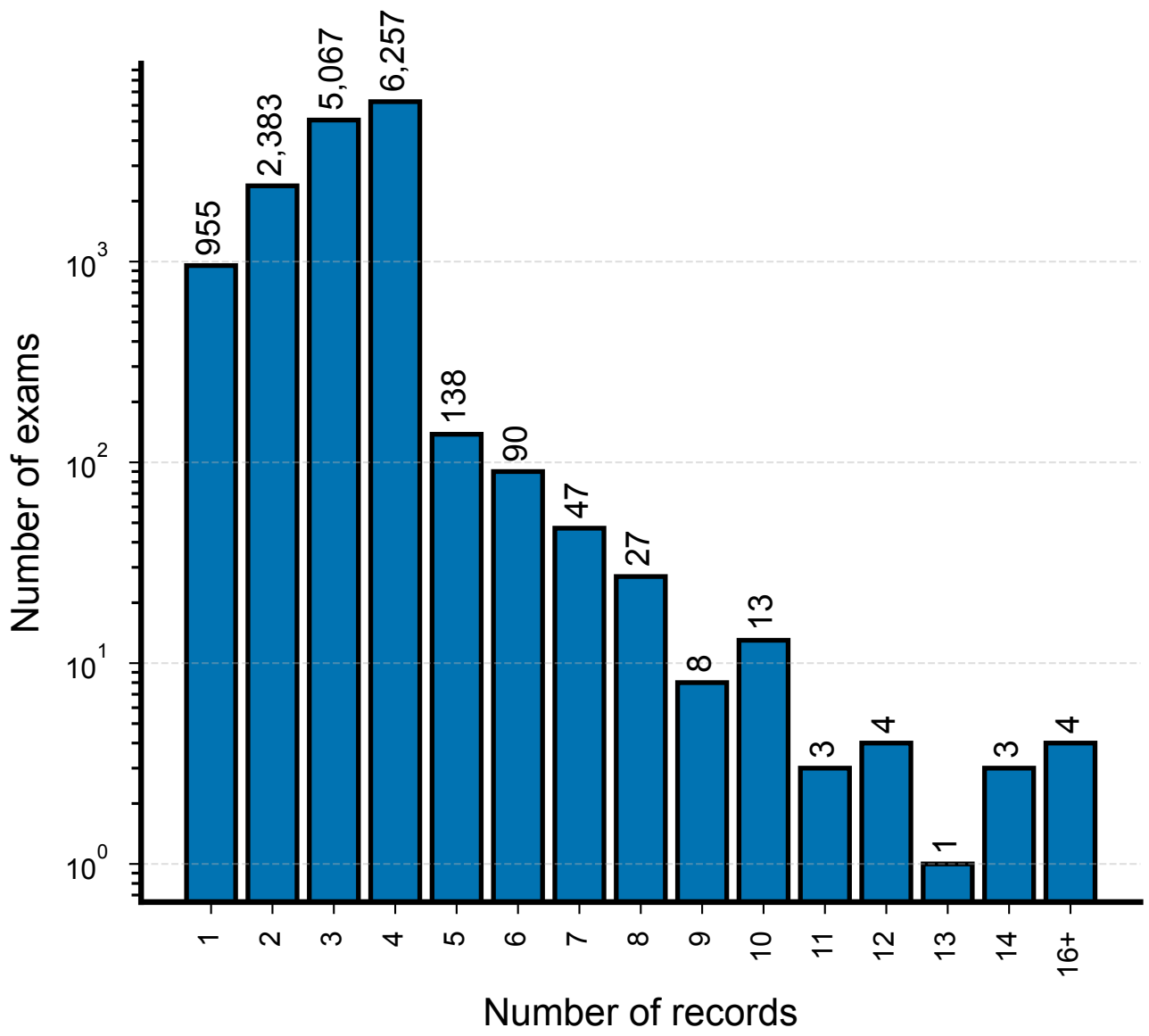

Supplementary Fig. 12. Distribution of the number of signal records per exam in the CODE-II-open dataset. Each exam may include multiple consecutive tracings recorded during the same clinical session.

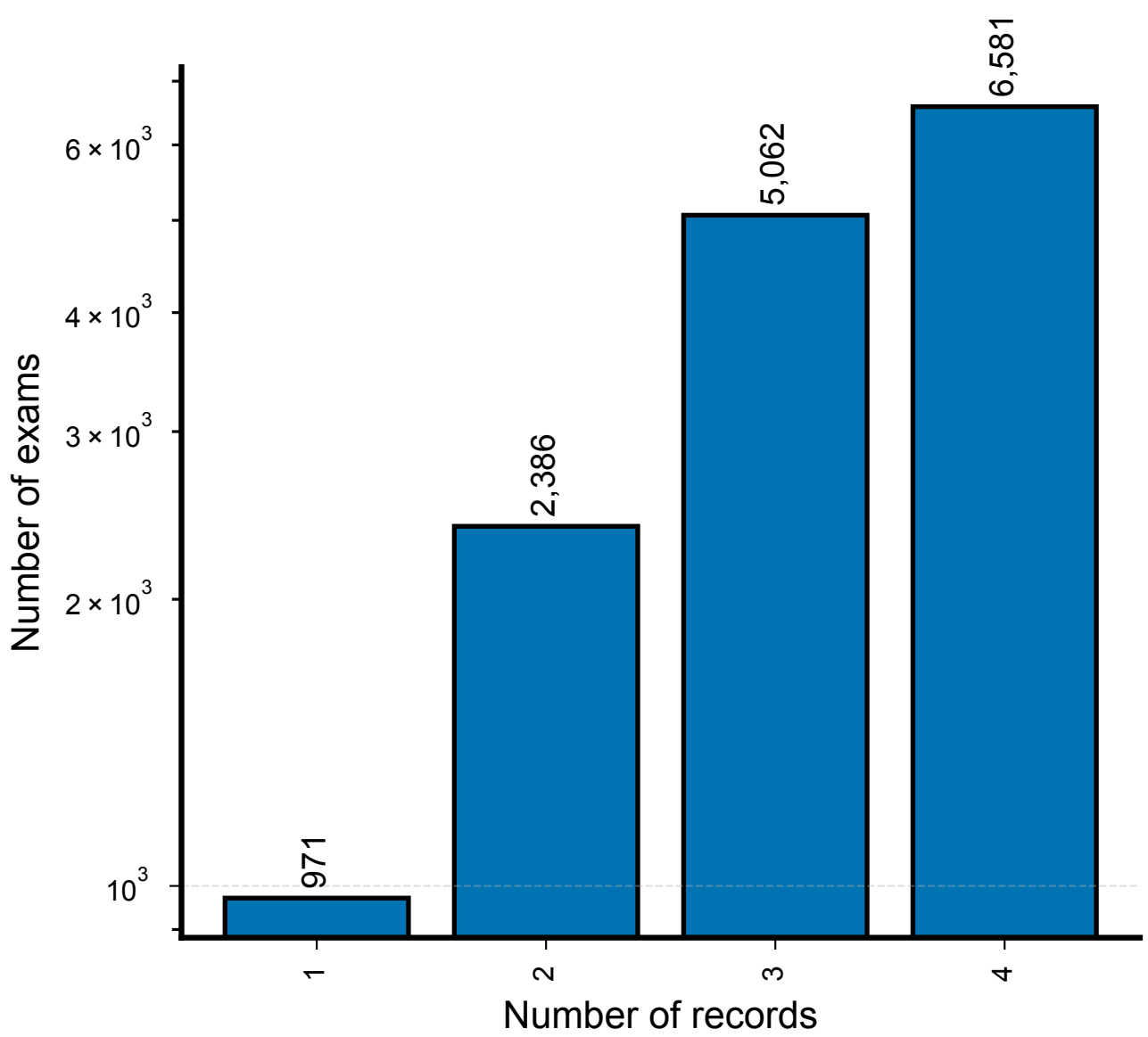

Supplementary Fig. 13. Final distribution of the number of tracings per exam in the CODE-II-open dataset after applying the pipeline that retains up to four tracings per exam.

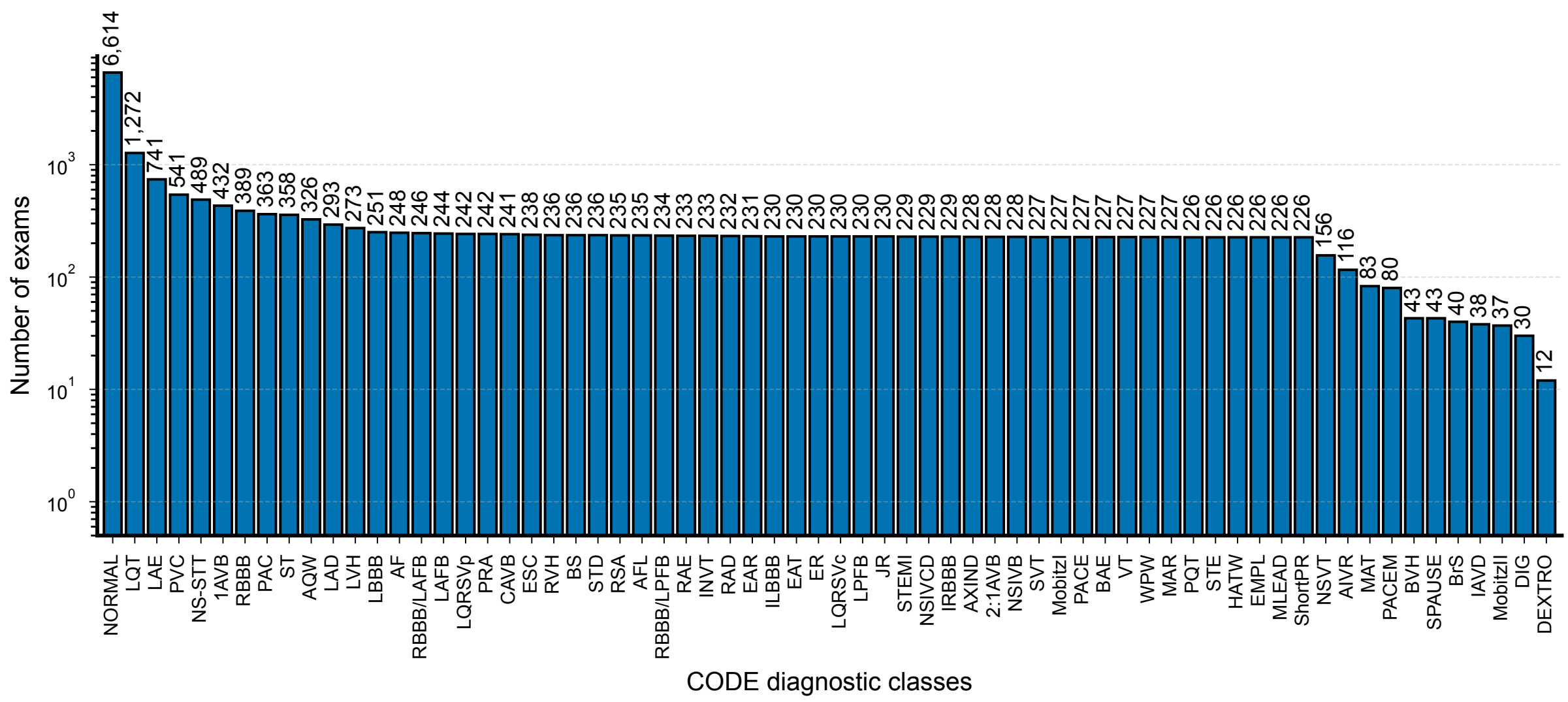

Supplementary Fig. 14. Number of exams per diagnostic class in the CODE-II-open dataset. Class assignments are not mutually exclusive: a single exam may be associated with multiple diagnoses, except for Normal ECGs, which are assigned exclusively.

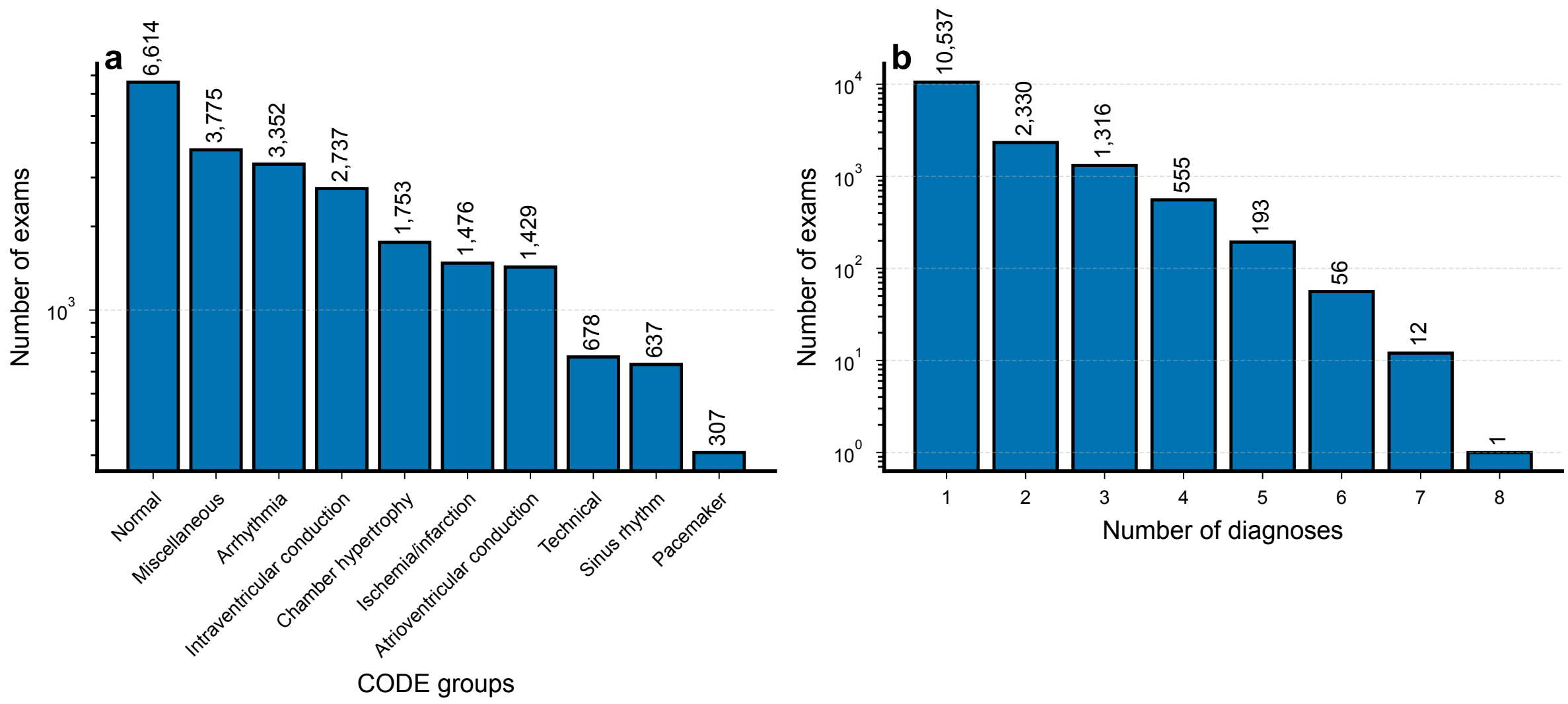

Supplementary Fig. 15. Distribution of CODE-II-open ECG exams by diagnostic group and number of assigned diagnoses. (a) Number of exams per CODE diagnostic group (note that exams may appear in multiple groups), and (b) the number of exams with a given number of assigned CODE diagnoses.

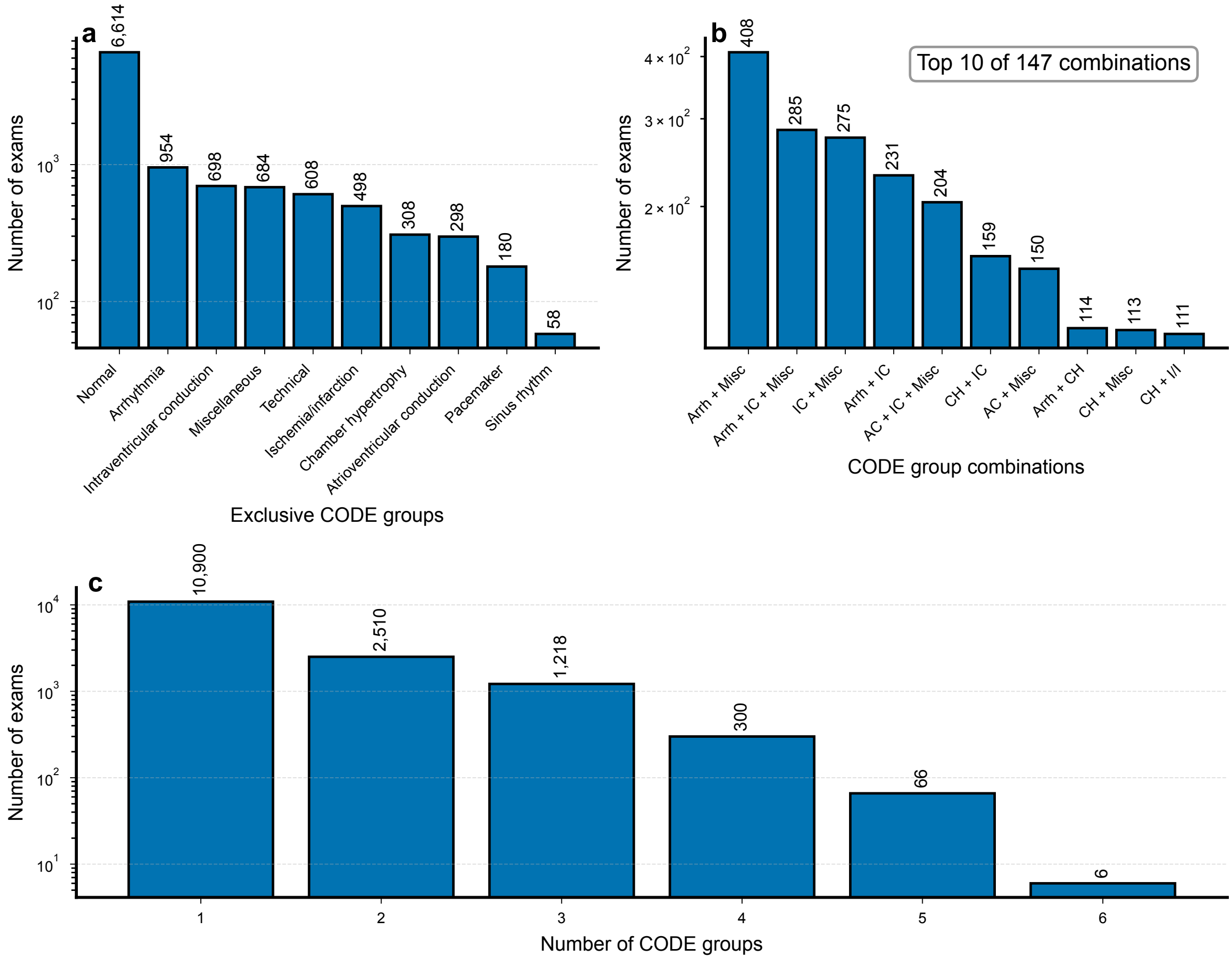

Supplementary Fig. 16. Analysis of group-level diagnostic label assignments in the CODE-II-open dataset. (a) Number of exams exclusively assigned to each of the 10 CODE groups. (b) Ten most frequent non-exclusive group combinations, among 147 distinct combinations observed. Group names were abbreviated (using the first four letters or a combination of initials) and joined by "+" to denote co-occurrence. (c) Distribution of the number of CODE groups assigned per exam, indicating how frequently exams belong to multiple groups.

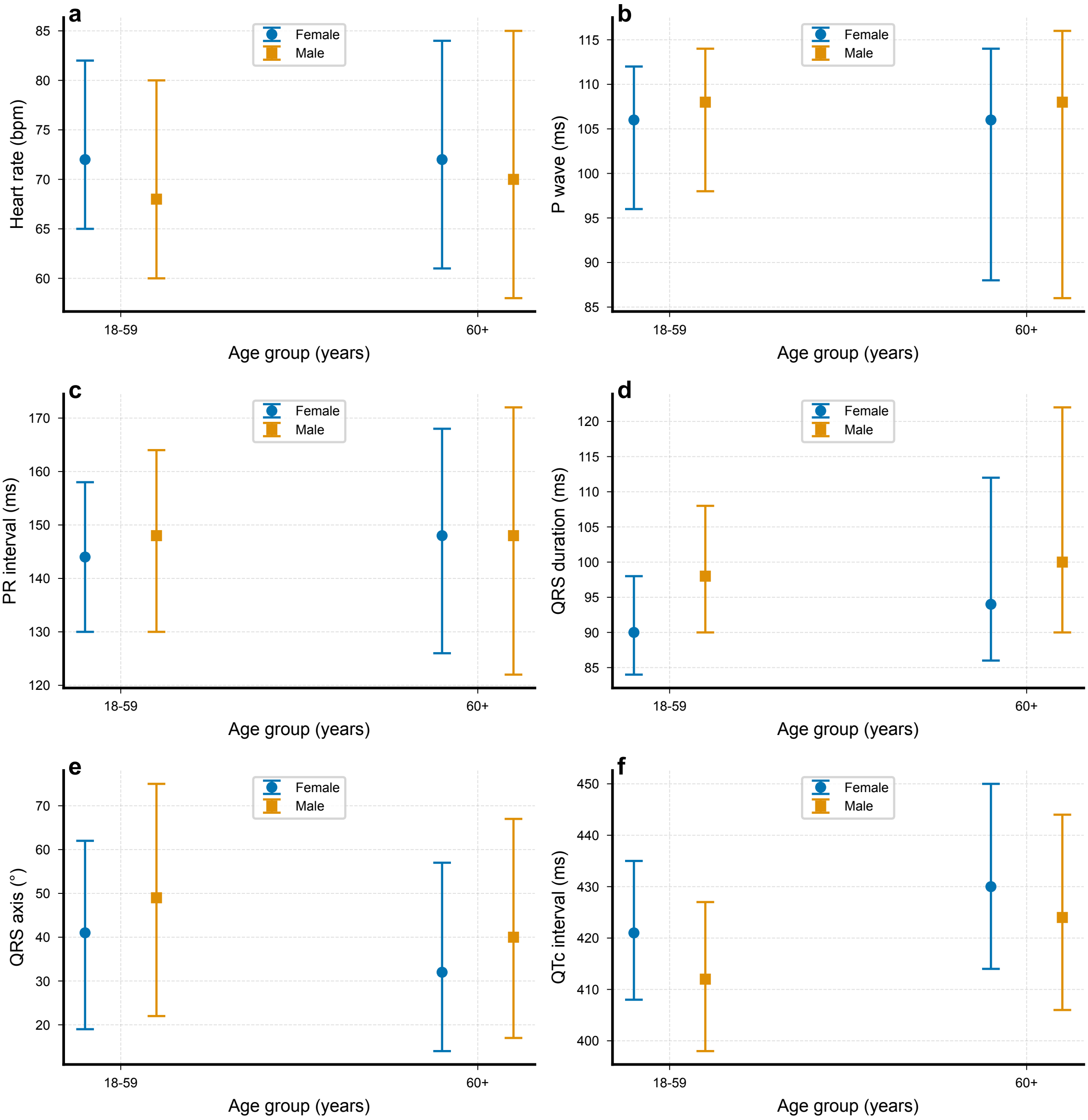

Supplementary Fig. 17. Median and interquartile range (IQR) of electrocardiographic measurements in the CODE-II-open dataset, stratified by age group and sex. (a) Heart rate (beats per minute); (b) P wave duration (milliseconds), reflecting atrial depolarization; (c) PR interval (ms), reflecting atrioventricular conduction time; (d) QRS duration (ms), representing ventricular depolarization; (e) QRS axis (degrees), showing the mean frontal plane electrical axis; and (f) Corrected QT interval (QTc, ms), adjusted for heart rate.

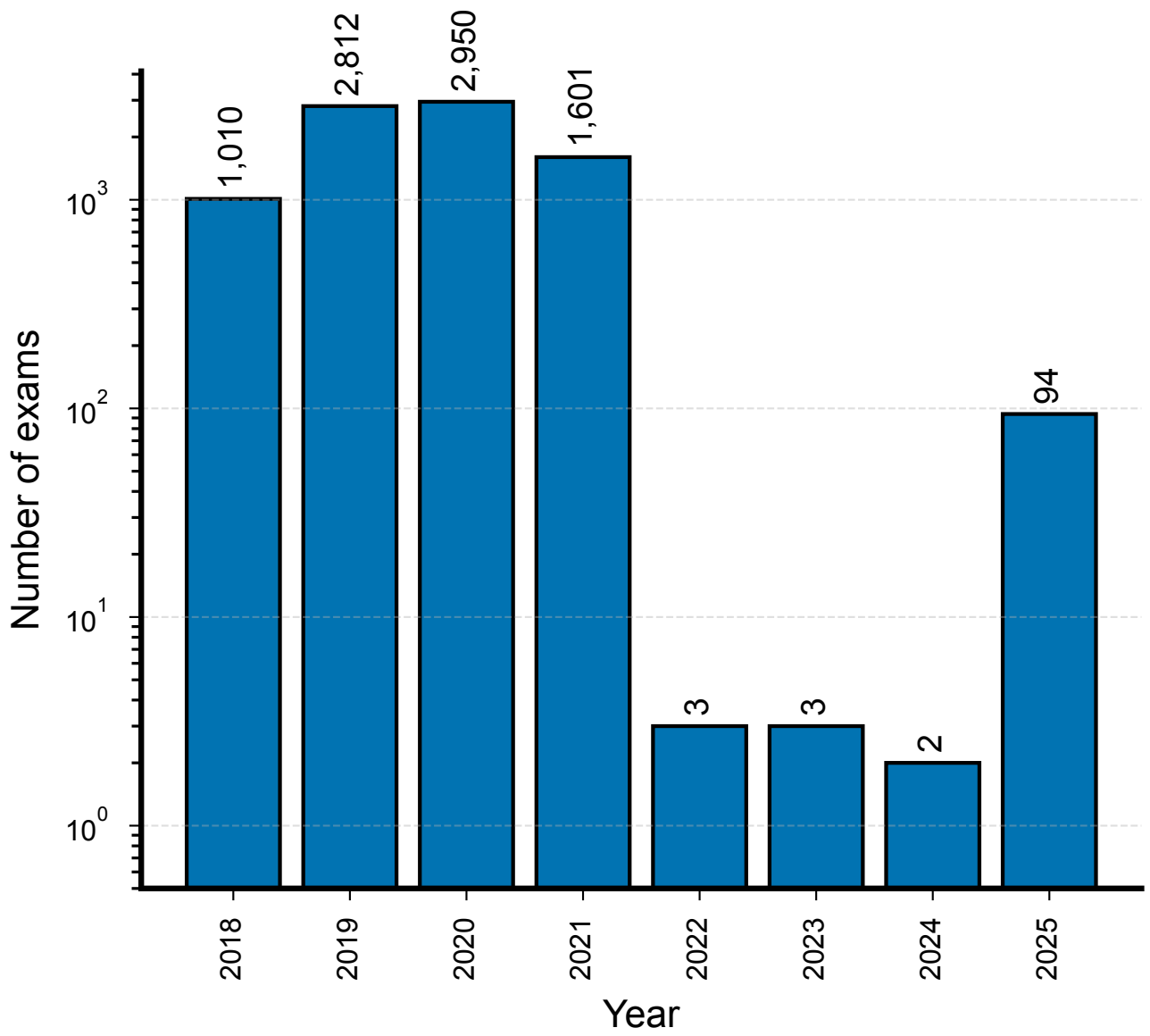

Supplementary Fig. 18. Annual distribution of ECG exams in the CODE-II-test dataset (2018–2025).

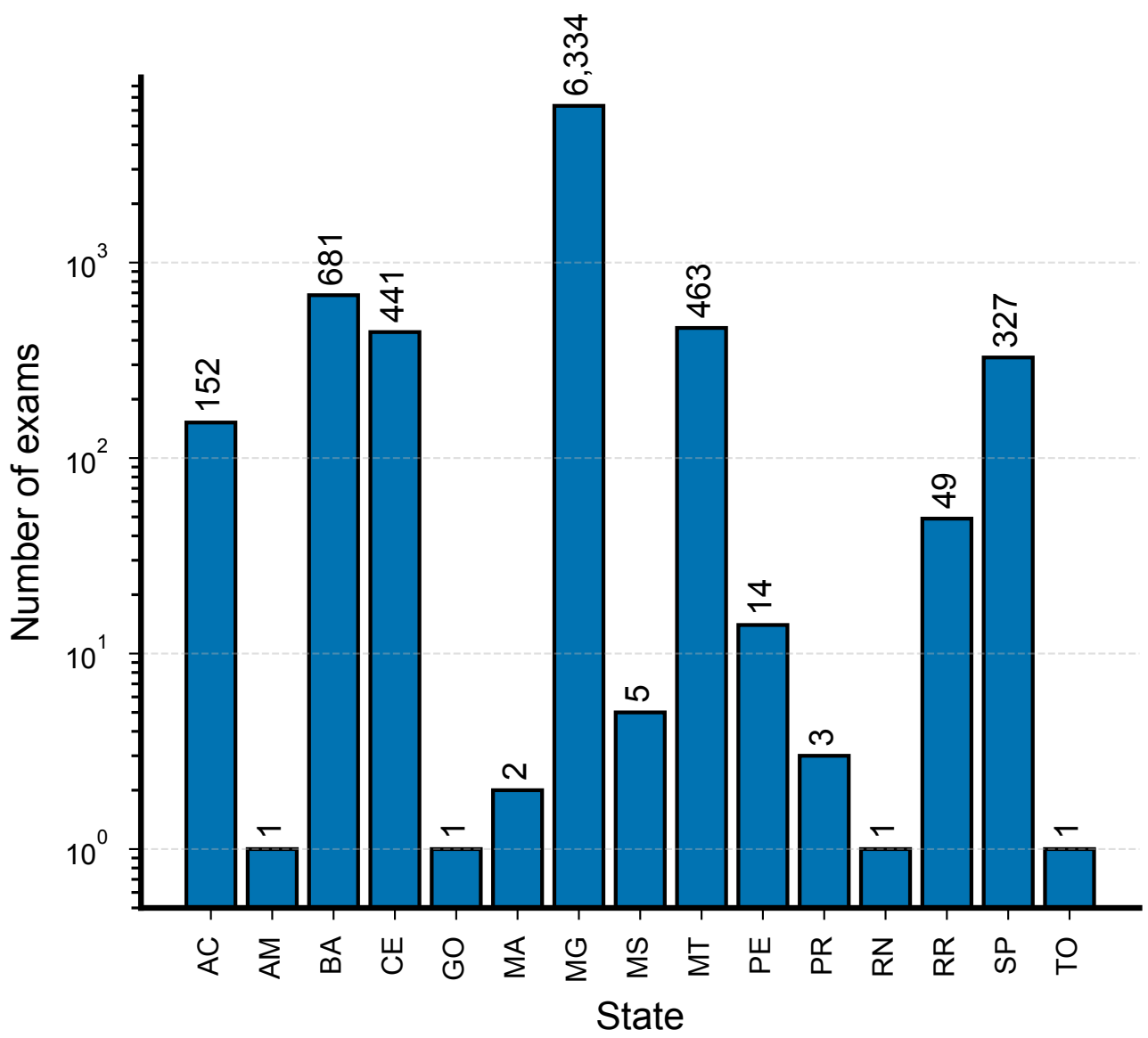

Supplementary Fig. 19. Geographic distribution of ECG exams in the CODE-II-test dataset across Brazilian states.

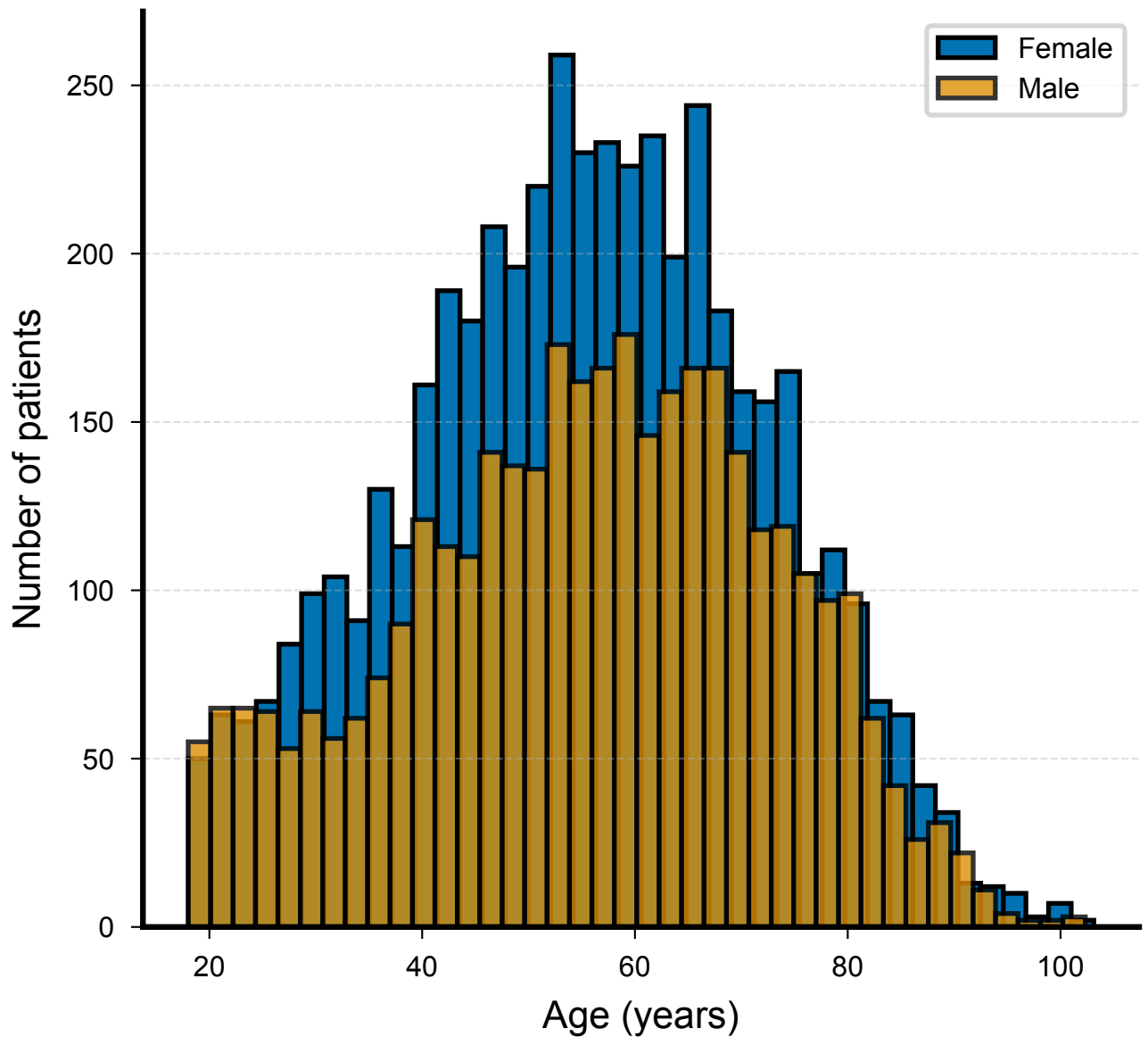

Supplementary Fig. 20. Age distribution of patients in the CODE-II-test dataset, stratified by sex.

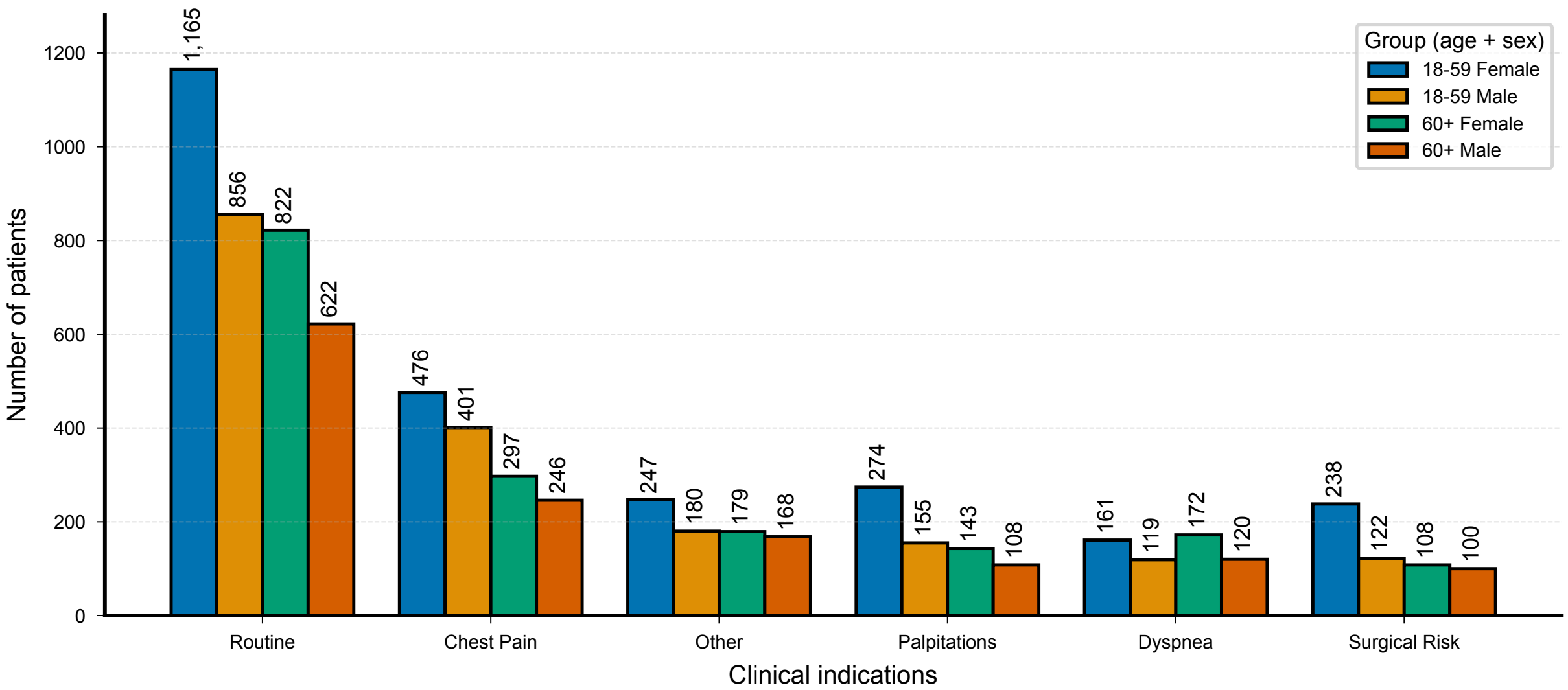

Supplementary Fig. 21. Distribution of ECG exam indications in the CODE-II-test dataset by age group and sex.

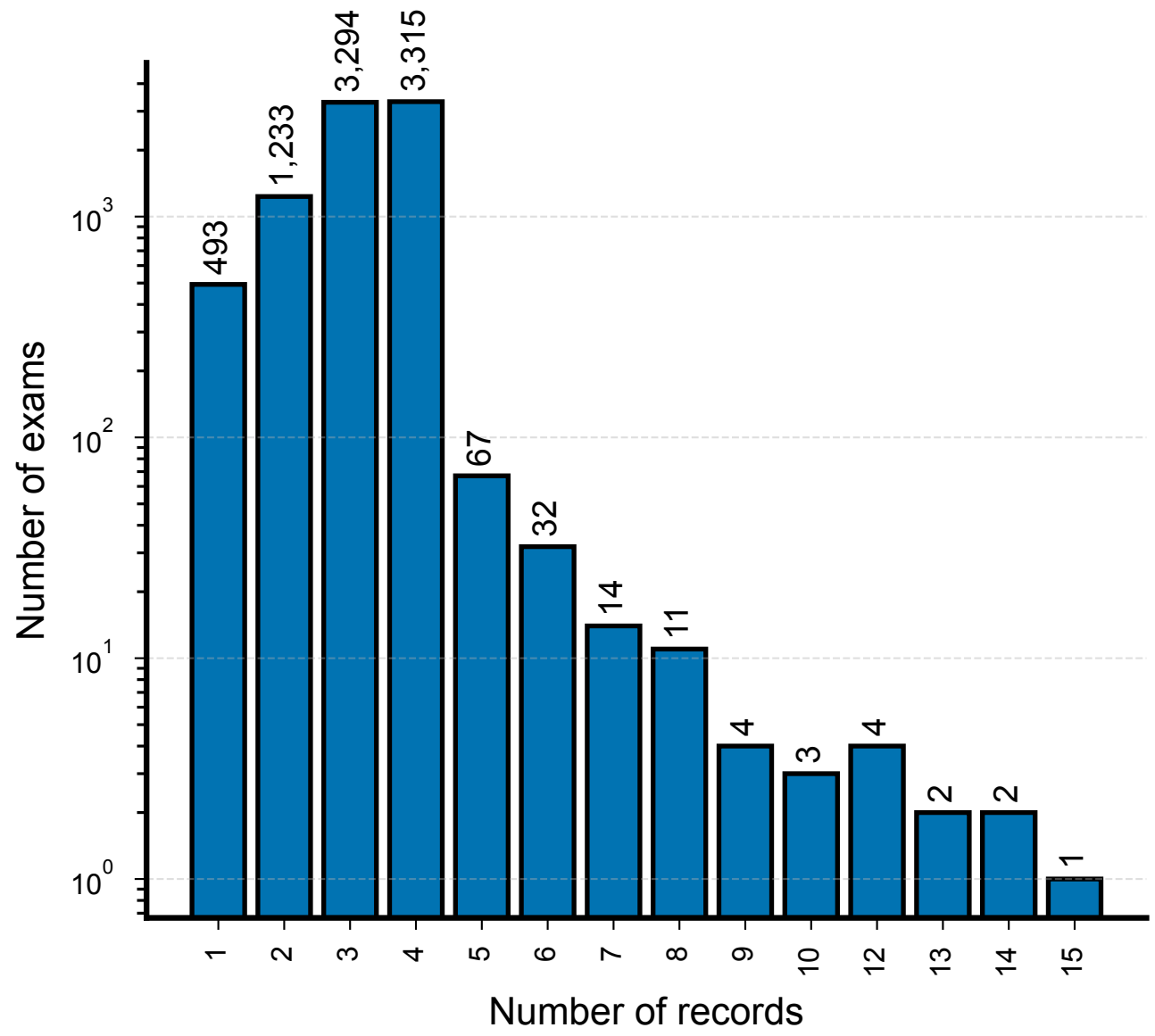

Supplementary Fig. 22. Number of signal records per exam in the CODE-II-test dataset.

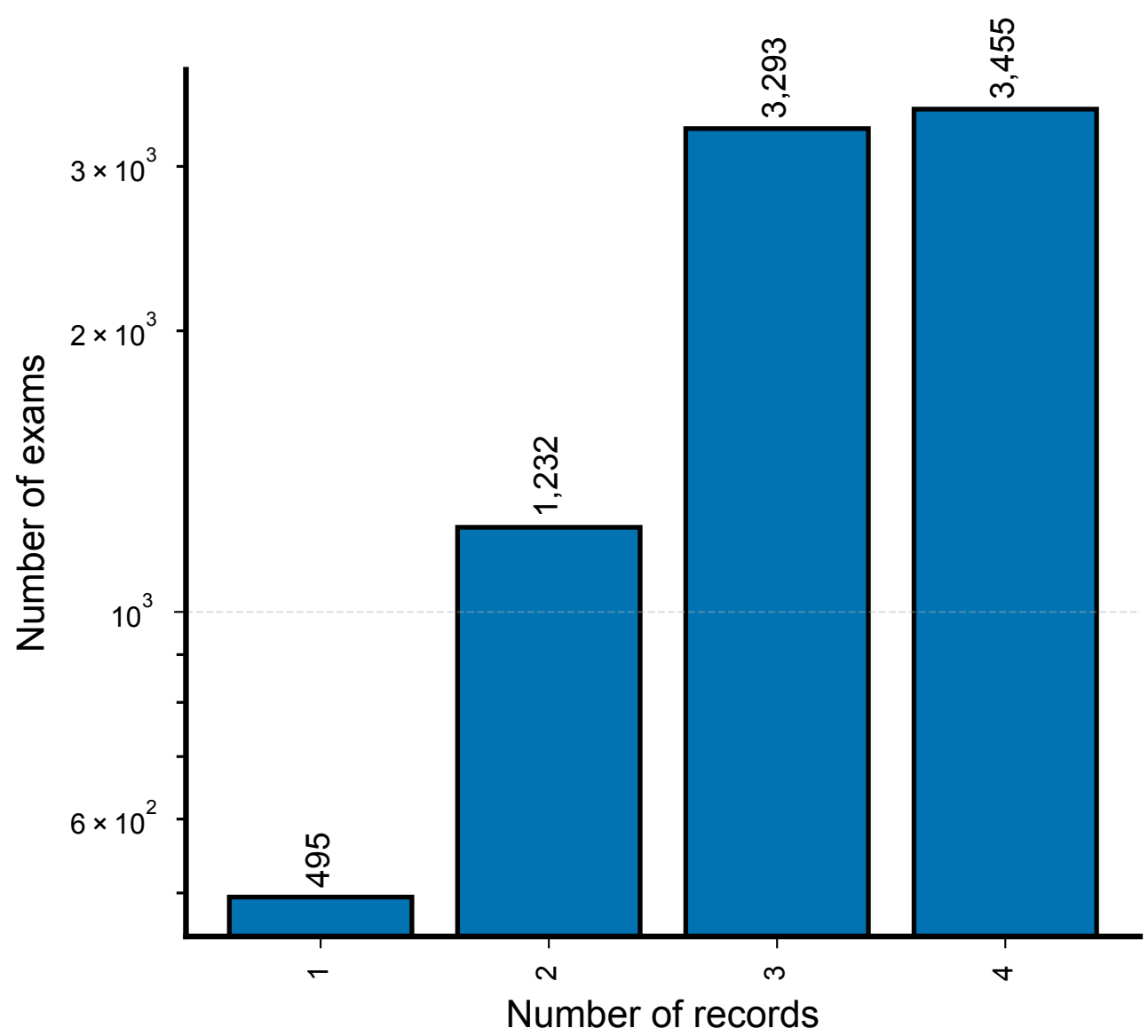

Supplementary Fig. 23. Final distribution of the number of tracings per exam in the CODE-II-test dataset after applying the pipeline that retains up to four tracings per exam.

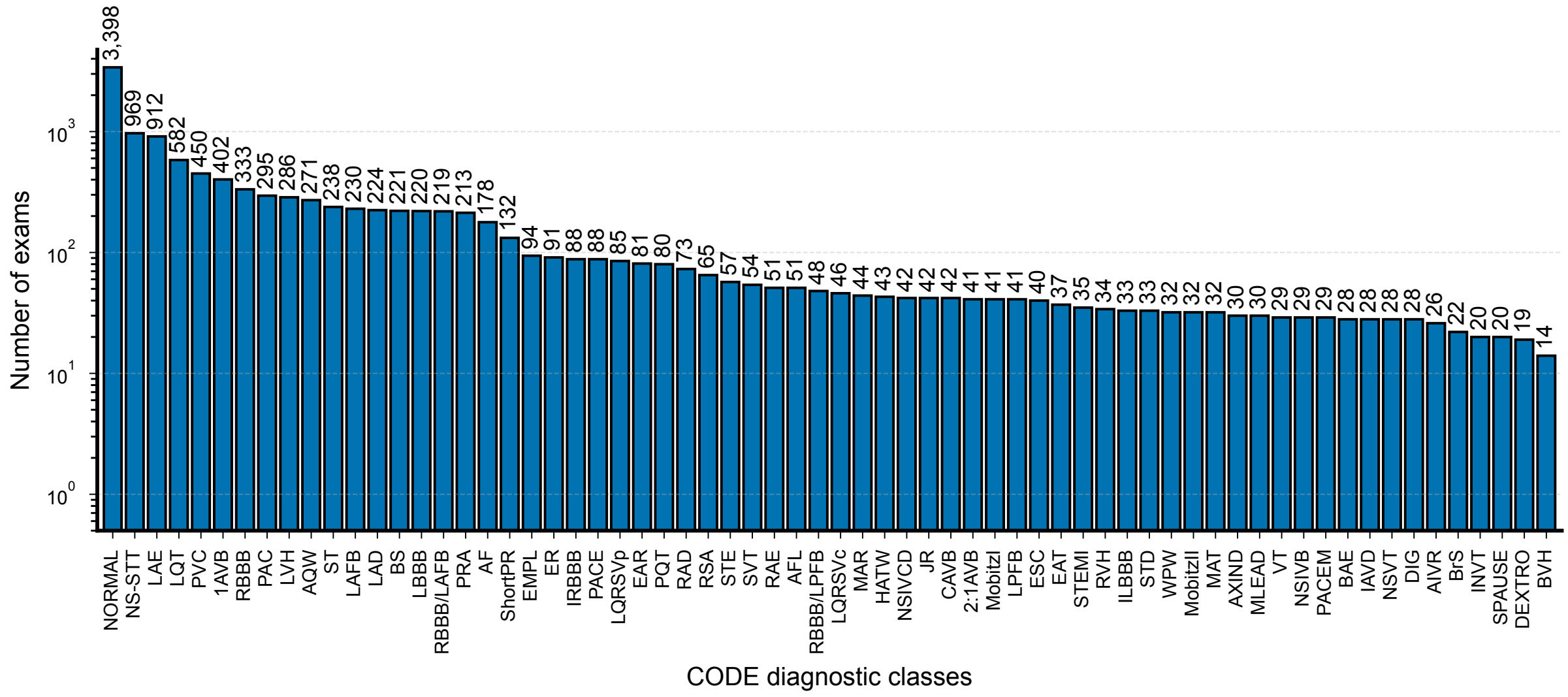

Supplementary Fig. 24. Distribution of ECG exams per diagnostic class in the CODE-II-test dataset. Class assignments are not mutually exclusive: a single exam may be associated with multiple diagnoses, except for Normal ECGs, which are assigned exclusively.

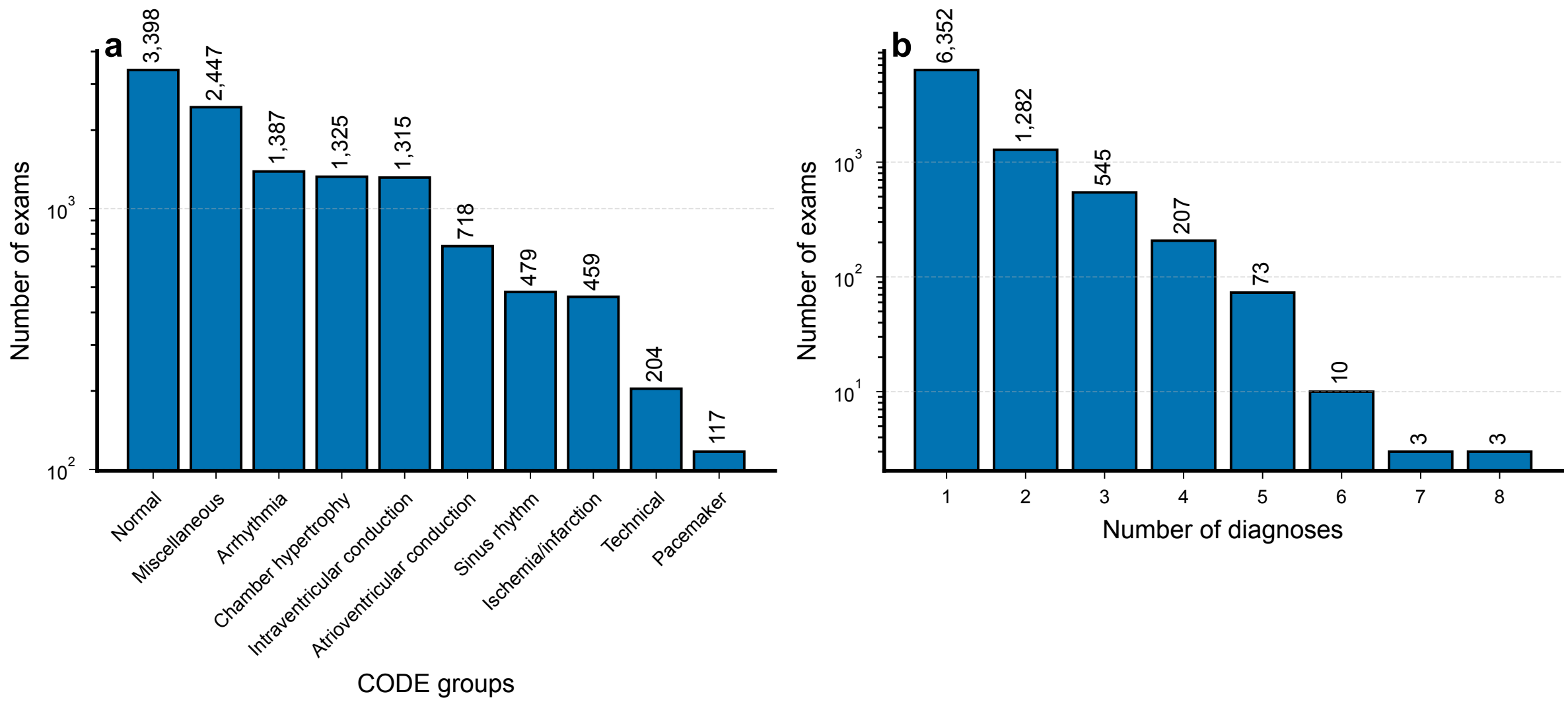

Supplementary Fig. 25. Distribution of ECG exams in the CODE-II-test dataset by diagnostic group and by number of assigned diagnoses. (a) Number of exams per CODE diagnostic group (exams may appear in multiple groups). (b) Distribution of exams by number of assigned CODE diagnoses.

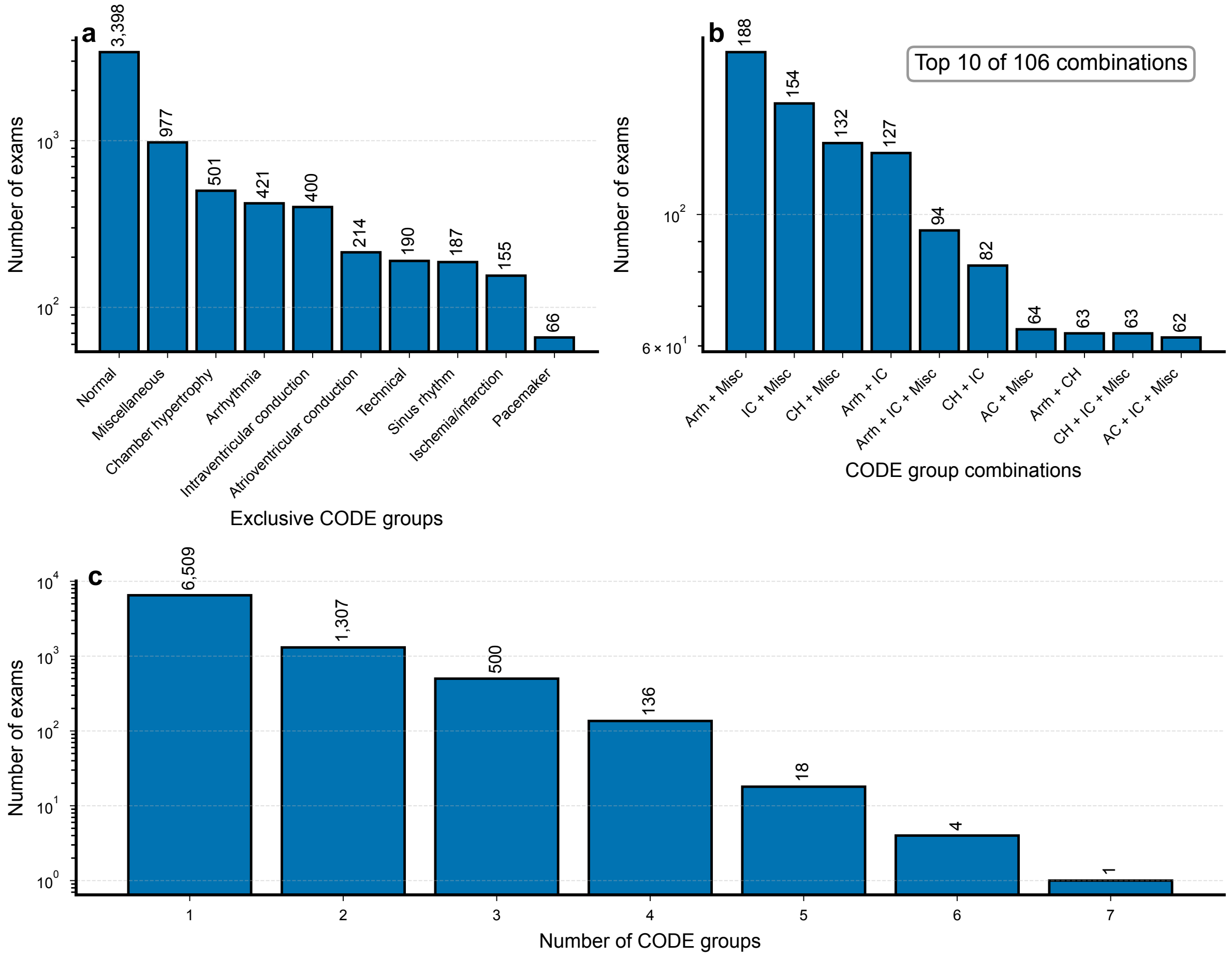

Supplementary Fig. 26. Group-level diagnostic label assignments in the CODE-II-test dataset. (a) Number of exams exclusively assigned to each of the 10 CODE groups. (b) Ten most frequent non-exclusive group combinations, among 106 distinct combinations observed. Group names were abbreviated (using the first four letters or a combination of initials) and joined by "+" to denote co-occurrence. (c) Distribution of the number of CODE groups assigned per exam, indicating how frequently exams belong to multiple groups.

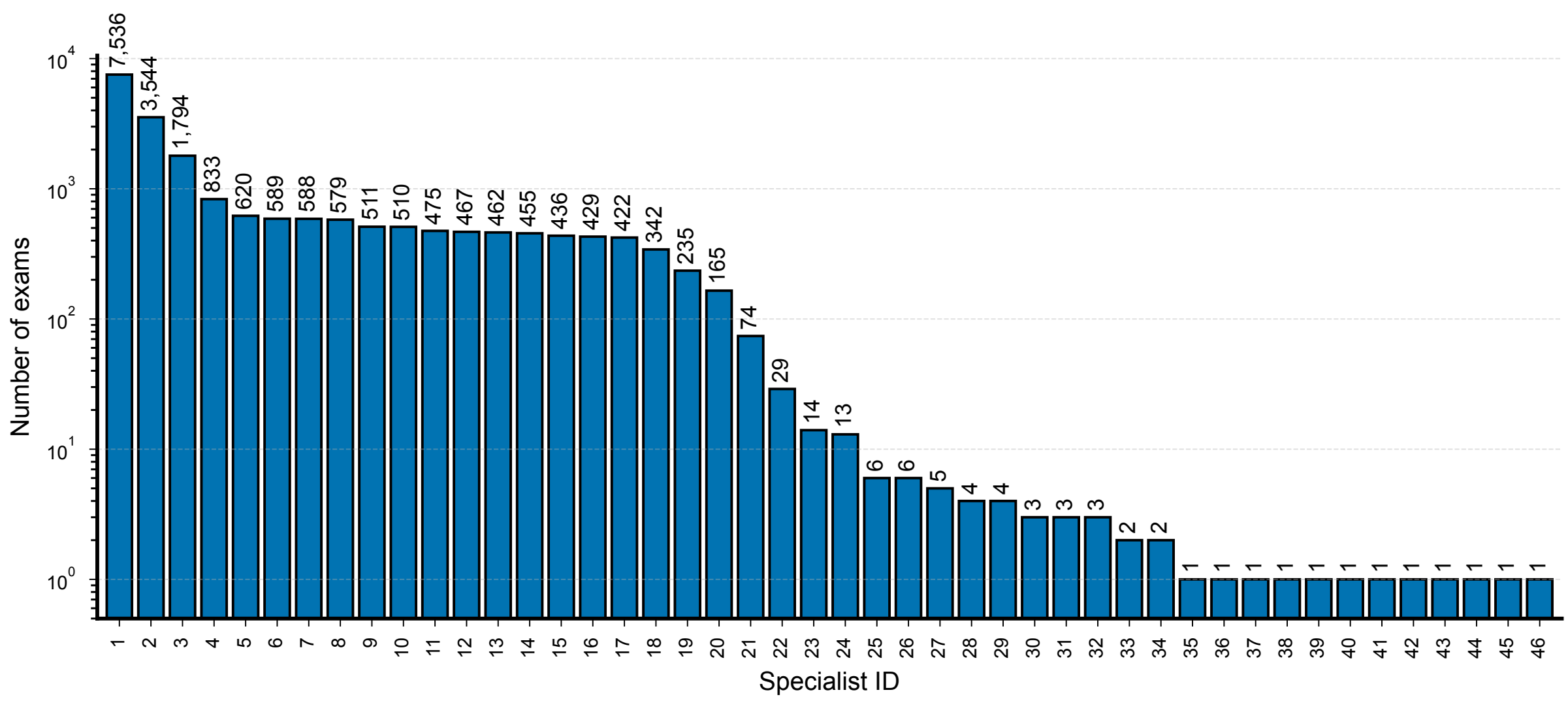

Supplementary Fig. 27. Distribution of the number of exams annotated per cardiologist in the CODE-II-test dataset (n = 46 cardiologists).

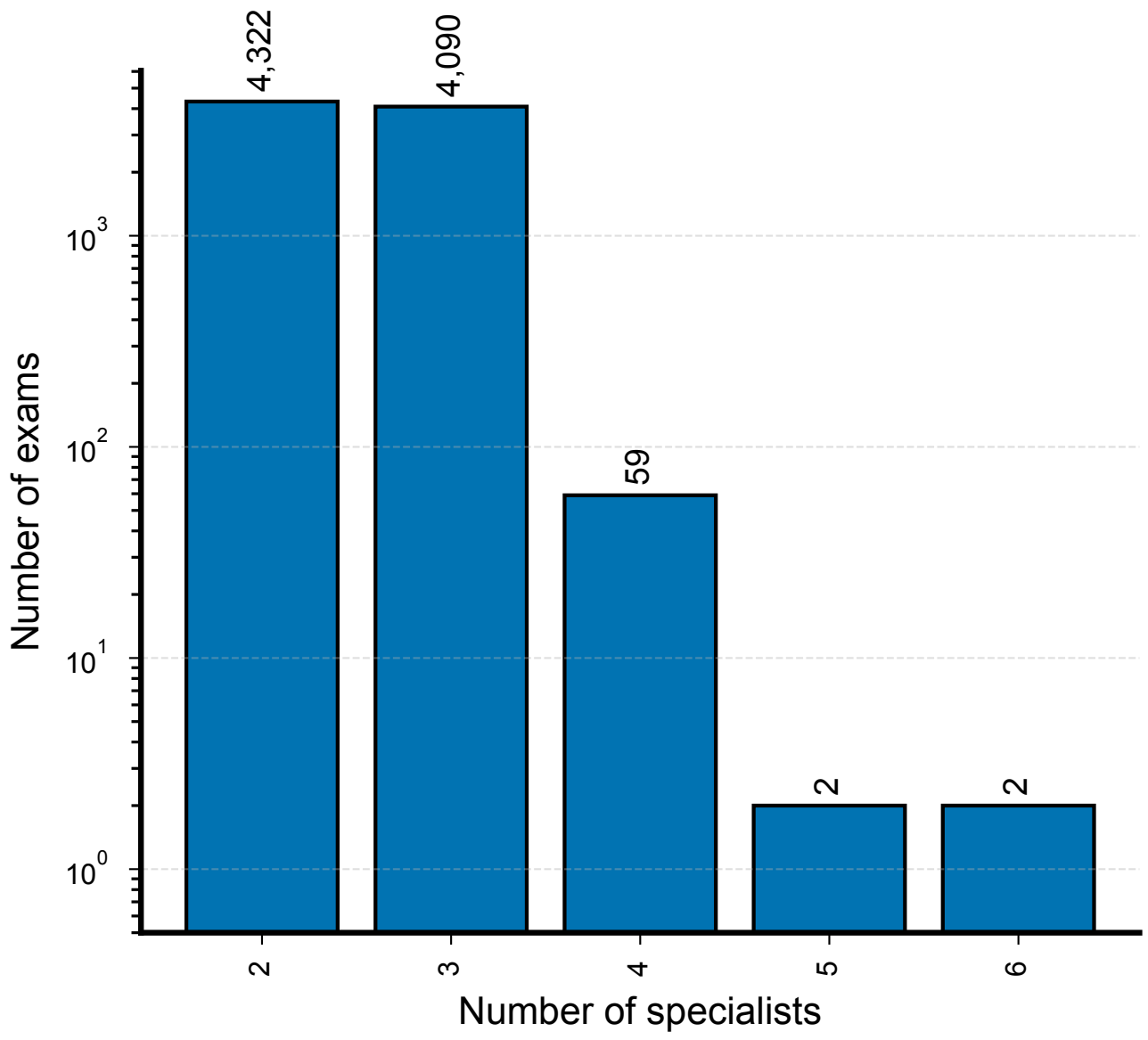

Supplementary Fig. 28. Distribution of the number of cardiologists per exam in the CODE-II-test dataset.

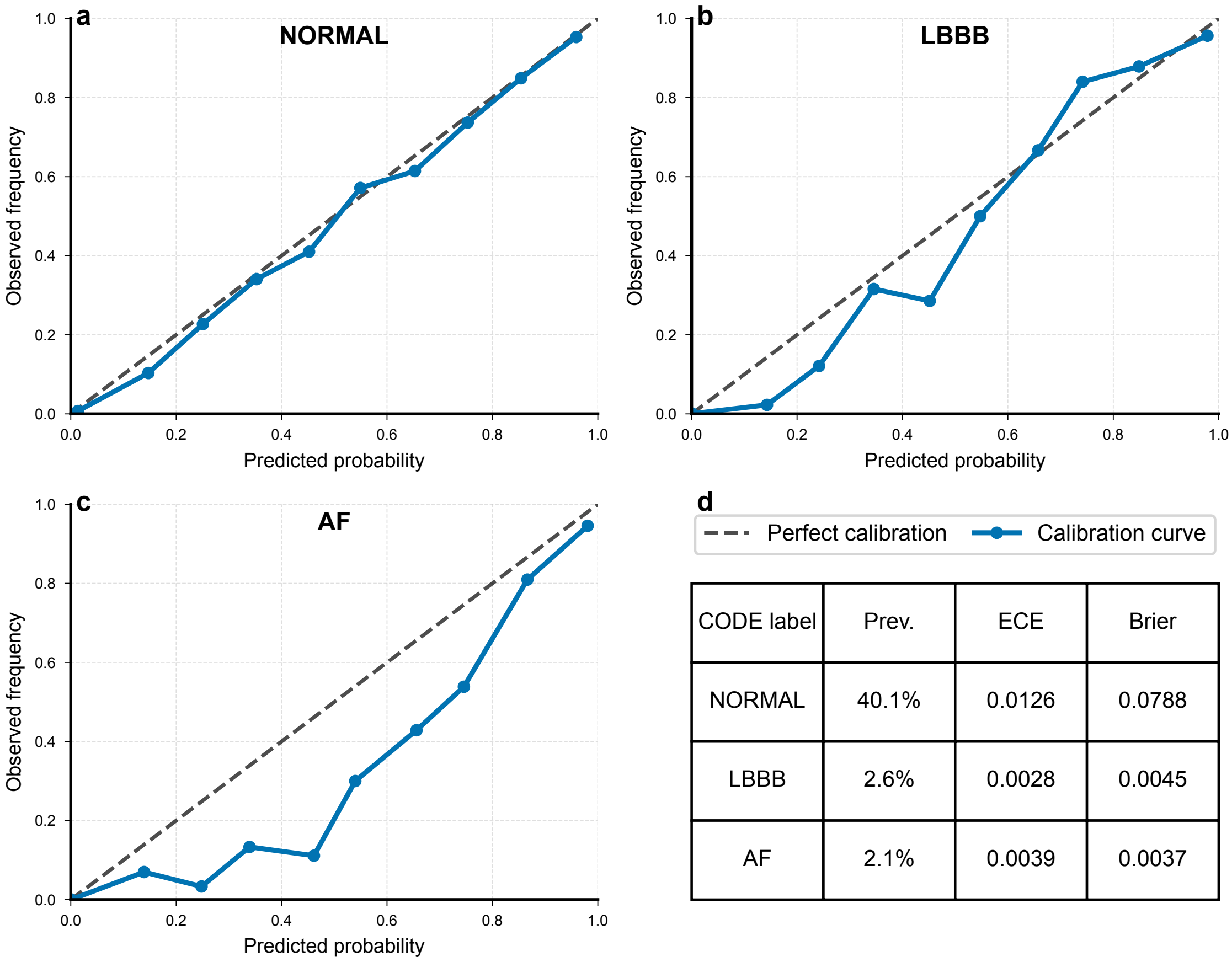

CODE label	Prev.	ECE	Brier
NORMAL	40.1%	0.0126	0.0788
LBBB	2.6%	0.0028	0.0045
AF	2.1%	0.0039	0.0037

Supplementary Fig. 29. Calibration curves for three representative diagnostic classes in the CODE-II-test dataset. Panels (a–c) show reliability diagrams for NORMAL, LBBB, and AF, respectively, plotting observed frequency against predicted probability. The dashed diagonal line indicates perfect calibration, while the solid line represents the empirical calibration curve obtained by binning predicted probabilities. Panel (d) summarizes, for these classes, the test-set prevalence, expected calibration error (ECE), and Brier score.

Supplementary Tables

Supplementary Table 1. Self-reported clinical comorbidities of patients in the CODE-II dataset, stratified by age group (18–59 years and $\geq$ 60 years) and sex, based on the first ECG exam. For hypertension, dyslipidemia, and diabetes, comorbidity status also inferred from reported medication usage.

	18–59		60+		Total			% of Patients with Condition		
Condition	Female	Male	Female	Male	Female	Male	All	Female	Male	All
Hypertension	298,696	159,570	319,837	220,367	618,533	379,937	998,470	61.95	38.05	47.69
Diabetes	79,382	39,982	111,306	62,594	190,688	102,576	293,264	65.02	34.98	14.01
Smoking	50,485	56,017	26,775	37,793	77,260	93,810	171,070	45.16	54.84	8.17
Dyslipidemia	25,576	13,090	39,733	21,604	65,309	34,694	100,003	65.31	34.69	4.78
Myocardial Infarction	9,558	7,625	9,620	11,481	19,178	19,106	38,284	50.09	49.91	1.83
Chagas Disease	6,582	3,710	7,235	4,491	13,817	8,201	22,018	62.75	37.25	1.05
COPD	3,985	1,820	4,403	3,732	8,388	5,552	13,940	60.17	39.83	0.67

Supplementary Table 2. Summary of the 66 CODE diagnostic classes in the CODE-II dataset, including class descriptions, labels, and the number and percentage of exams in the full dataset (n = 2,735,269), as well as class-wise counts of unique patients and ECG exams in the training and validation sets obtained using the proposed patient-exclusive stratified split. The complete table is also provided as Supplementary Data 1 (external Excel file, *Supplementary_Data_1.xlsx*).

		CODE-II dataset			Validation set		Training set	
CODE statement description	CODE label	Total Exams	Unique Patients	% of All Exams	Validation Exams	Validation Patients	Training Exams	Training Patients
ECG within normal limits for age and sex	NORMAL	1,364,623	1,174,017	49.89	272,925	267,612	1,091,698	906,405
Nonspecific ST-T abnormality	NS-STT	364,537	318,576	13.327	78,366	66,179	286,171	252,397
Left atrial enlargement	LAE	289,219	252,908	10.574	76,890	64,925	212,329	187,983
Left ventricular hypertrophy	LVH	110,180	94,222	4.028	25,492	21,598	84,688	72,624
Sinus tachycardia	ST	107,212	97,654	3.92	26,476	23,776	80,736	73,878
Premature ventricular complex(es)	PVC	105,608	92,877	3.861	31,248	26,398	74,360	66,479
First-degree atrioventricular block	1AVB	103,581	84,334	3.787	31,838	24,925	71,743	59,409
Prolonged QT interval	LQT	95,911	77,887	3.506	37,738	29,667	58,173	48,220
Premature atrial complex(es)	PAC	85,858	77,713	3.139	24,053	21,187	61,805	56,526
Pathological Q waves	AQW	72,558	60,443	2.653	22,007	17,679	50,551	42,764
Left-axis deviation	LAD	72,254	63,769	2.642	18,956	16,199	53,298	47,570
Left anterior fascicular block	LAFB	70,278	58,910	2.569	16,919	14,069	53,359	44,841
Primary repolarization abnormalities	PRA	63,966	56,038	2.339	15,377	13,396	48,589	42,642
Left bundle branch block	LBBB	60,804	45,165	2.223	16,420	11,609	44,384	33,556
Sinus bradycardia	BS	56,907	51,025	2.08	13,974	12,215	42,933	38,810
Atrial fibrillation	AF	52,513	36,283	1.92	15,613	10,240	36,900	26,043
Right bundle branch block	RBBB	49,473	38,918	1.809	10,817	8,241	38,656	30,677
Short PR interval	ShortPR	39,484	36,415	1.444	7,897	7,098	31,587	29,317
Low QRS voltage in peripheral leads	LQRSVp	33,745	30,288	1.234	9,105	7,972	24,640	22,316
Right bundle branch and left anterior fascicular blocks	RBBB/LAFB	32,701	25,020	1.196	8,246	5,961	24,455	19,059
Extremity electrode reversal/misplaced precordial electrode(s)	EMPL	30,581	28,025	1.118	6,116	5,677	24,465	22,348
Poor-quality ECG	PQT	29,226	25,755	1.068	5,845	5,012	23,381	20,743
Incomplete right bundle branch block	IRBBB	23,965	22,023	0.876	5,572	5,118	18,393	16,905
Low QRS voltage in precordial leads	LQRSVc	22,259	20,740	0.814	5,544	5,097	16,715	15,643
Ectopic atrial rhythm	EAR	21,628	20,364	0.791	4,969	4,607	16,659	15,757
Right-axis deviation	RAD	14,713	13,380	0.538	3,628	3,297	11,085	10,083
Early repolarization	ER	13,794	13,204	0.504	2,759	2,626	11,035	10,578
Incomplete left bundle branch block	ILBBB	12,255	11,567	0.448	3,242	3,005	9,013	8,562
Paced rhythm	PACE	10,566	7,224	0.386	2,234	1,589	8,332	5,635
ST-segment elevation	STE	7,567	6,573	0.277	2,074	1,802	5,493	4,771
Nonspecific intraventricular block	NSIVB	5,893	5,454	0.215	2,005	1,827	3,888	3,627
Right superior axis	RSA	5,706	4,979	0.209	1,942	1,672	3,764	3,307
Atrial flutter	AFL	5,304	4,182	0.194	1,605	1,221	3,699	2,961
Nonspecific intraventricular conduction delay	NSIVCD	4,811	4,653	0.176	1,234	1,186	3,577	3,467
Supraventricular tachycardia	SVT	4,675	3,948	0.171	1,554	1,276	3,121	2,672
ST-elevation myocardial infarction	STEMI	4,141	3,628	0.151	1,125	991	3,016	2,637
ST-segment depression	STD	3,824	3,345	0.14	1,191	1,046	2,633	2,299
Missing lead(s)	MLEAD	3,217	2,960	0.118	643	610	2,574	2,350
Left posterior fascicular block	LPFB	3,103	2,950	0.113	701	661	2,402	2,289
Right atrial enlargement	RAE	3,005	2,858	0.11	904	840	2,101	2,018
Right bundle branch and left posterior fascicular blocks	RBBB/LPFB	2,947	2,667	0.108	711	623	2,236	2,044
T-wave inversions	INVT	2,508	2,164	0.092	684	576	1,824	1,588
Ventricular preexcitation - Wolf Parkinson White	WPW	2,421	2,010	0.089	484	401	1,937	1,609
Ectopic atrial tachycardia, unifocal	EAT	2,284	2,136	0.084	770	712	1,514	1,424
Hyperacute T waves	HATW	1,963	1,744	0.072	486	426	1,477	1,318
Complete atrioventricular block (third-degree)	CAVB	1,793	1,570	0.066	632	551	1,161	1,019
Junctional rhythm	JR	1,679	1,589	0.061	446	422	1,233	1,167
Indeterminate axis	AXIND	1,252	1,208	0.046	407	384	845	824
Second-degree atrioventricular block, Mobitz type I (Wenckebach)	MobitzI	1,107	1,008	0.04	323	300	784	708
Escape complex(es)	ESC	966	960	0.035	299	296	667	664
Right ventricular hypertrophy	RVH	903	843	0.033	254	236	649	607
2:1 atrioventricular block	2:1AVB	592	538	0.022	231	208	361	330
Ventricular tachycardia	VT	482	417	0.018	117	99	365	318
Biatrial enlargement	BAE	392	387	0.014	105	104	287	283
Multifocal atrial rhythm	MAR	381	376	0.014	122	121	259	255
Nonsustained ventricular tachycardia	NSVT	295	281	0.011	130	123	165	158
Accelerated idioventricular rhythm	AIVR	202	197	0.007	68	67	134	130
Pacemaker malfunction	PACEM	137	126	0.005	31	27	106	99
Multifocal atrial tachycardia	MAT	117	117	0.004	34	34	83	83
Second-degree atrioventricular block, Mobitz type II	MobitzII	63	63	0.002	26	26	37	37
Brugada syndrome	BrS	63	59	0.002	15	13	48	46
Biventricular hypertrophy	BVH	57	56	0.002	18	17	39	39
Sinus pause	SPAUSE	56	55	0.002	14	13	42	42
Isorhythmic atrioventricular dissociation	IAVD	48	47	0.002	13	13	35	34
Digitalis effect	DIG	44	44	0.002	14	14	30	30
Dextrocardia	DEXTRO	29	28	0.001	6	6	23	22

Supplementary Table 3. Group-level distribution of CODE diagnostic assignments at the patient level, stratified by age (18–59 and $\geq$ 60 years) and sex. Columns report counts by stratum, totals, the percentage of patients within each diagnosis group (by sex and overall), and the percentage relative to all patients in CODE-II. Diagnostic groups are not mutually exclusive; patients may belong to multiple groups, except for the Normal group, which is exclusive by definition.

	18–59		60+		Total			% of Patients in Diagnosis Group		
Diagnosis Group	Female	Male	Female	Male	Female	Male	All	Female	Male	All
Normal	524,401	290,750	173,357	108,427	697,758	399,177	1,096,935	63.61	36.39	52.39
Miscellaneous	136,276	81,092	131,424	104,268	267,700	185,360	453,060	59.09	40.91	21.64
Chamber hypertrophy	48,350	65,685	72,809	83,572	121,159	149,257	270,416	44.8	55.2	12.92
Intraventricular conduction	24,776	38,086	56,863	66,707	81,639	104,793	186,432	43.79	56.21	8.9
Arrhythmia	28,529	24,156	57,770	66,572	86,299	90,728	177,027	48.75	51.25	8.45
Sinus rhythm	43,169	35,547	23,010	22,221	66,179	57,768	123,947	53.39	46.61	5.92
Atrioventricular conduction	27,767	18,439	27,641	31,981	55,408	50,420	105,828	52.36	47.64	5.05
Ischemia/infarction	9,054	12,555	16,810	20,846	25,864	33,401	59,265	43.64	56.36	2.83
Technical	14,306	10,173	10,023	8,652	24,329	18,825	43,154	56.38	43.62	2.06
Pacemaker	594	603	2,352	2,504	2,946	3,107	6,053	48.67	51.33	0.29

Supplementary Table 4. Prevalence of self-reported or inferred comorbidities across age and sex groups in the CODE-II-open dataset.

	18–59		60+		Total			% of Patients with Condition		
Condition	Female	Male	Female	Male	Female	Male	All	Female	Male	All
Hypertension	1,676	1,151	2,326	1,974	4,002	3,125	7,127	56.15	43.85	47.51
Diabetes	429	260	771	535	1,200	795	1,995	60.15	39.85	13.3
Smoking	318	450	208	355	526	805	1,331	39.52	60.48	8.87
Dyslipidemia	157	97	266	201	423	298	721	58.67	41.33	4.81
Myocardial Infarction	54	77	77	141	131	218	349	37.54	62.46	2.33
Chagas Disease	67	58	116	75	183	133	316	57.91	42.09	2.11
COPD	41	13	58	40	99	53	152	65.13	34.87	1.01

Supplementary Table 5. Summary of the 66 CODE diagnostic classes in the CODE-II-open dataset, including class descriptions, labels, and the number and percentage of exams in the dataset ($n = 15{,}000$), as well as class-wise counts of unique patients and ECG exams in the training and validation sets obtained using the proposed patient-exclusive stratified split. The complete table is also provided as Supplementary Data 2 (external Excel file, *Supplementary_Data_2.xlsx*).

		CODE-II-open dataset		Validation set	Training set
CODE statement description	CODE label	Total Exams	% of All Exams	Validation Exams	Training Exams
ECG within normal limits for age and sex	NORMAL	6,614	44.09	1,488	5,126
Prolonged QT interval	LQT	1,272	8.48	398	874
Left atrial enlargement	LAE	741	4.94	213	528
Premature ventricular complex(es)	PVC	541	3.61	159	382
Nonspecific ST-T abnormality	NS-STT	489	3.26	179	310
First-degree atrioventricular block	1AVB	432	2.88	134	298
Right bundle branch block	RBBB	389	2.59	105	284
Premature atrial complex(es)	PAC	363	2.42	125	238
Sinus tachycardia	ST	358	2.39	130	228
Pathological Q waves	AQW	326	2.17	97	229
Left-axis deviation	LAD	293	1.95	77	216
Left ventricular hypertrophy	LVH	273	1.82	78	195
Left bundle branch block	LBBB	251	1.67	69	182
Atrial fibrillation	AF	248	1.65	64	184
Right bundle branch and left anterior fascicular blocks	RBBB/LAFB	246	1.64	59	187
Left anterior fascicular block	LAFB	244	1.63	63	181
Low QRS voltage in peripheral leads	LQRSVp	242	1.61	57	185
Primary repolarization abnormalities	PRA	242	1.61	61	181
Complete atrioventricular block (third-degree)	CAVB	241	1.61	53	188
Escape complex(es)	ESC	238	1.59	49	189
Sinus bradycardia	BS	236	1.57	54	182
Right ventricular hypertrophy	RVH	236	1.57	47	189
ST-segment depression	STD	236	1.57	47	189
Right superior axis	RSA	235	1.57	47	188
Atrial flutter	AFL	235	1.57	48	187
Right bundle branch and left posterior fascicular blocks	RBBB/LPFB	234	1.56	45	189
T-wave inversions	INVT	233	1.55	48	185
Right atrial enlargement	RAE	233	1.55	50	183
Right-axis deviation	RAD	232	1.55	46	186
Ectopic atrial rhythm	EAR	231	1.54	45	186
Low QRS voltage in precordial leads	LQRSVc	230	1.53	46	184
Ectopic atrial tachycardia, unifocal	EAT	230	1.53	45	185
Left posterior fascicular block	LPFB	230	1.53	47	183
Junctional rhythm	JR	230	1.53	46	184
Incomplete left bundle branch block	ILBBB	230	1.53	46	184
Early repolarization	ER	230	1.53	45	185
Incomplete right bundle branch block	IRBBB	229	1.53	45	184
Nonspecific intraventricular conduction delay	NSIVCD	229	1.53	46	183
ST-elevation myocardial infarction	STEMI	229	1.53	46	183
2:1 atrioventricular block	2:1AVB	228	1.52	46	182
Nonspecific intraventricular block	NSIVB	228	1.52	46	182
Indeterminate axis	AXIND	228	1.52	45	183
Ventricular preexcitation - Wolf Parkinson White	WPW	227	1.51	45	182
Ventricular tachycardia	VT	227	1.51	46	181
Multifocal atrial rhythm	MAR	227	1.51	46	181
Biatrial enlargement	BAE	227	1.51	46	181
Second-degree atrioventricular block, Mobitz type I (Wenckebach)	MobitzI	227	1.51	46	181
Paced rhythm	PACE	227	1.51	45	182
Supraventricular tachycardia	SVT	227	1.51	45	182
Hyperacute T waves	HATW	226	1.51	45	181
Missing lead(s)	MLEAD	226	1.51	45	181
Extremity electrode reversal/misplaced precordial electrode(s)	EMPL	226	1.51	45	181
ST-segment elevation	STE	226	1.51	45	181
Poor-quality ECG	PQT	226	1.51	45	181
Short PR interval	ShortPR	226	1.51	45	181
Nonsustained ventricular tachycardia	NSVT	156	1.04	45	111
Accelerated idioventricular rhythm	AIVR	116	0.77	36	80
Multifocal atrial tachycardia	MAT	83	0.55	23	60
Pacemaker malfunction	PACEM	80	0.53	16	64
Biventricular hypertrophy	BVH	43	0.29	10	33
Sinus pause	SPAUSE	43	0.29	8	35
Brugada syndrome	BrS	40	0.27	7	33
Isorhythmic atrioventricular dissociation	IAVD	38	0.25	10	28
Second-degree atrioventricular block, Mobitz type II	MobitzII	37	0.25	12	25
Digitalis effect	DIG	30	0.2	12	18
Dextrocardia	DEXTRO	12	0.08	3	9

Supplementary Table 6. Prevalence of self-reported or inferred comorbidities across age and sex groups in the CODE-II-test dataset.

	18–59		60+		Total			% of Patients with Condition		
Condition	Female	Male	Female	Male	Female	Male	All	Female	Male	All
Hypertension	1,006	594	1,244	880	2,250	1,474	3,724	60.42	39.58	43.94
Chagas Disease	376	302	333	213	709	515	1,224	57.92	42.08	14.44
Diabetes	225	127	403	230	628	357	985	63.76	36.24	11.62
Smoking	160	212	103	144	263	356	619	42.49	57.51	7.3
Dyslipidemia	121	74	206	107	327	181	508	64.37	35.63	5.99
Myocardial Infarction	37	34	50	62	87	96	183	47.54	52.46	2.16
COPD	12	8	35	20	47	28	75	62.67	37.33	0.88

Supplementary Table 7. Summary of the 66 CODE diagnostic classes in the CODE-II-test dataset, including class descriptions, labels, and the number and percentage of exams in the dataset ($n = 8{,}475$). The complete table is also provided as Supplementary Data 3 (external Excel file, *Supplementary_Data_3.xlsx*).

		CODE-II-test dataset	
CODE statement description	CODE label	Total Exams	% of All Exams
ECG within normal limits for age and sex	NORMAL	3,398	40.09
Nonspecific ST-T abnormality	NS-STT	969	11.43
Left atrial enlargement	LAE	912	10.76
Prolonged QT interval	LQT	582	6.87
Premature ventricular complex(es)	PVC	450	5.31
First-degree atrioventricular block	1AVB	402	4.74
Right bundle branch block	RBBB	333	3.93
Premature atrial complex(es)	PAC	295	3.48
Left ventricular hypertrophy	LVH	286	3.37
Pathological Q waves	AQW	271	3.2
Sinus tachycardia	ST	238	2.81
Left anterior fascicular block	LAFB	230	2.71
Left-axis deviation	LAD	224	2.64
Sinus bradycardia	BS	221	2.61
Left bundle branch block	LBBB	220	2.6
Right bundle branch and left anterior fascicular blocks	RBBB/LAFB	219	2.58
Primary repolarization abnormalities	PRA	213	2.51
Atrial fibrillation	AF	178	2.1
Short PR interval	ShortPR	132	1.56
Extremity electrode reversal/misplaced precordial electrode(s)	EMPL	94	1.11
Early repolarization	ER	91	1.07
Paced rhythm	PACE	88	1.04
Incomplete right bundle branch block	IRBBB	88	1.04
Low QRS voltage in peripheral leads	LQRSVp	85	1.0
Ectopic atrial rhythm	EAR	81	0.96
Poor-quality ECG	PQT	80	0.94
Right-axis deviation	RAD	73	0.86
Right superior axis	RSA	65	0.77
ST-segment elevation	STE	57	0.67
Supraventricular tachycardia	SVT	54	0.64
Right atrial enlargement	RAE	51	0.6
Atrial flutter	AFL	51	0.6
Right bundle branch and left posterior fascicular blocks	RBBB/LPFB	48	0.57
Low QRS voltage in precordial leads	LQRSVc	46	0.54
Multifocal atrial rhythm	MAR	44	0.52
Hyperacute T waves	HATW	43	0.51
Junctional rhythm	JR	42	0.5
Complete atrioventricular block (third-degree)	CAVB	42	0.5
Nonspecific intraventricular conduction delay	NSIVCD	42	0.5
2:1 atrioventricular block	2:1AVB	41	0.48
Left posterior fascicular block	LPFB	41	0.48
Second-degree atrioventricular block, Mobitz type I (Wenckebach)	MobitzI	41	0.48
Escape complex(es)	ESC	40	0.47
Ectopic atrial tachycardia, unifocal	EAT	37	0.44
ST-elevation myocardial infarction	STEMI	35	0.41
Right ventricular hypertrophy	RVH	34	0.4
Incomplete left bundle branch block	ILBBB	33	0.39
ST-segment depression	STD	33	0.39
Multifocal atrial tachycardia	MAT	32	0.38
Second-degree atrioventricular block, Mobitz type II	MobitzII	32	0.38
Ventricular preexcitation - Wolf Parkinson White	WPW	32	0.38
Indeterminate axis	AXIND	30	0.35
Missing lead(s)	MLEAD	30	0.35
Ventricular tachycardia	VT	29	0.34
Pacemaker malfunction	PACEM	29	0.34
Nonspecific intraventricular block	NSIVB	29	0.34
Biatrial enlargement	BAE	28	0.33
Digitalis effect	DIG	28	0.33
Nonsustained ventricular tachycardia	NSVT	28	0.33
Isorhythmic atrioventricular dissociation	IAVD	28	0.33
Accelerated idioventricular rhythm	AIVR	26	0.31
Brugada syndrome	BrS	22	0.26
Sinus pause	SPAUSE	20	0.24
T-wave inversions	INVT	20	0.24
Dextrocardia	DEXTRO	19	0.22
Biventricular hypertrophy	BVH	14	0.17

Supplementary Table 8. Cardiologist-level performance on the CODE-II-test under the fair-scope definition. For each cardiologist, we report the number of exams reviewed, the number of diagnostic classes encountered, and micro- and macro-averaged precision, recall, specificity, F1 score, and NPV computed only over classes actually seen by that cardiologist. To avoid small-sample inflation, the table lists cardiologists with ≥ 100 reviewed exams. The complete list, including all cardiologists, is provided in Supplementary Data 4 (external Excel file, Supplementary_Data_4.xlsx).

			Micro-averaged					Macro-averaged				
Cardiologist ID	Exams reviewed	Classes seen	Precision	Recall	Specificity	F1 score	NPV	Precision	Recall	Specificity	F1 score	NPV
1	8,368	65	0.821	0.924	0.996	0.869	0.998	0.805	0.915	0.995	0.844	0.998
2	3,583	52	0.986	0.990	1.000	0.988	1.000	0.961	0.973	0.999	0.966	1.000
3	1,831	65	0.962	0.985	0.999	0.973	1.000	0.955	0.980	0.999	0.964	1.000
4	847	63	0.966	0.991	0.999	0.978	1.000	0.955	0.993	0.999	0.972	1.000
5	627	56	0.981	0.991	1.000	0.986	1.000	0.967	0.974	0.999	0.970	1.000
6	602	38	0.975	0.985	0.999	0.980	0.999	0.974	0.981	0.999	0.975	0.999
7	596	62	0.928	0.993	0.998	0.959	1.000	0.935	0.996	0.998	0.961	1.000
8	587	51	0.974	0.990	0.999	0.982	1.000	0.946	0.974	0.999	0.957	1.000
9	529	52	0.962	0.974	0.999	0.968	0.999	0.970	0.949	0.999	0.954	0.999
10	522	48	0.980	0.981	0.999	0.981	0.999	0.991	0.970	0.999	0.974	0.999
11	483	50	0.982	0.988	0.999	0.985	1.000	0.982	0.990	0.999	0.984	1.000
12	470	57	0.987	0.993	1.000	0.990	1.000	0.966	0.968	1.000	0.964	1.000
13	469	35	0.983	0.987	0.999	0.985	0.999	0.964	0.984	0.999	0.969	0.999
14	464	53	0.980	0.985	0.999	0.982	1.000	0.961	0.974	0.999	0.965	1.000
15	453	43	0.963	0.961	0.999	0.962	0.999	0.934	0.936	0.998	0.932	0.999
16	447	49	0.959	0.962	0.999	0.960	0.999	0.927	0.915	0.998	0.911	0.999
17	431	35	0.973	0.981	0.999	0.977	0.999	0.942	0.958	0.999	0.946	0.999
18	347	57	0.882	0.990	0.995	0.933	1.000	0.867	0.975	0.995	0.912	1.000
19	245	29	0.954	0.964	0.998	0.959	0.999	0.932	0.953	0.997	0.940	0.998
20	178	29	0.921	0.935	0.997	0.928	0.997	0.861	0.801	0.994	0.820	0.997

Supplementary Table 9. Per-class model performance on the CODE-II-test under the F1-max thresholding rule. For each diagnostic class, we report test prevalence (number and percentage), threshold-independent metrics (AUROC, AUPRC), and threshold-dependent metrics (F1-score, Recall/Sensitivity, Specificity, Precision, and NPV) computed using class-specific thresholds selected on the CODE-II validation split and applied unchanged to the test set. All metrics are reported with 95% confidence intervals estimated from 1,000 bootstrap resamples (shown in parentheses). The complete table is also provided as Supplementary Data 5 (external Excel file, *Supplementary_Data_5.xlsx*).

CODE statement description	CODE label	Total exams	Prevalence	Threshold	AUROC	AUPRC	F1 score	Recall	Specificity	Precision	NPV
ECG within normal limits for age and sex	NORMAL	3,398	40.09	0.2572	0.959 (0.955-0.963)	0.931 (0.923-0.938)	0.850 (0.841-0.858)	0.967 (0.960-0.972)	0.793 (0.782-0.804)	0.758 (0.745-0.770)	0.973 (0.967-0.977)
Nonspecific ST-T abnormality	NS-STT	969	11.43	0.2462	0.938 (0.932-0.945)	0.700 (0.669-0.732)	0.629 (0.608-0.650)	0.801 (0.775-0.826)	0.904 (0.897-0.911)	0.518 (0.494-0.542)	0.972 (0.968-0.976)
Left atrial enlargement	LAE	912	10.76	0.2886	0.898 (0.889-0.907)	0.531 (0.497-0.567)	0.541 (0.514-0.566)	0.638 (0.608-0.670)	0.913 (0.907-0.919)	0.469 (0.441-0.498)	0.954 (0.950-0.960)
Prolonged QT interval	LQT	582	6.87	0.2247	0.975 (0.971-0.978)	0.755 (0.717-0.788)	0.688 (0.660-0.714)	0.802 (0.773-0.831)	0.961 (0.957-0.965)	0.603 (0.568-0.636)	0.985 (0.982-0.988)
Premature ventricular complex(es)	PVC	450	5.31	0.1900	0.986 (0.979-0.992)	0.864 (0.833-0.892)	0.834 (0.808-0.859)	0.908 (0.881-0.933)	0.985 (0.982-0.988)	0.771 (0.736-0.807)	0.995 (0.993-0.996)
First-degree atrioventricular block	1AVB	402	4.74	0.3812	0.988 (0.982-0.993)	0.885 (0.858-0.910)	0.794 (0.763-0.824)	0.850 (0.816-0.887)	0.986 (0.983-0.988)	0.745 (0.705-0.786)	0.992 (0.990-0.994)
Right bundle branch block	RBBB	333	3.93	0.3898	0.996 (0.995-0.997)	0.921 (0.898-0.941)	0.829 (0.798-0.855)	0.898 (0.864-0.931)	0.989 (0.987-0.991)	0.770 (0.728-0.810)	0.996 (0.994-0.997)
Premature atrial complex(es)	PAC	295	3.48	0.1549	0.968 (0.957-0.978)	0.619 (0.559-0.671)	0.640 (0.599-0.678)	0.759 (0.712-0.809)	0.978 (0.975-0.981)	0.554 (0.507-0.602)	0.991 (0.989-0.993)
Left ventricular hypertrophy	LVH	286	3.37	0.2426	0.981 (0.975-0.986)	0.776 (0.733-0.819)	0.701 (0.659-0.742)	0.748 (0.697-0.797)	0.987 (0.984-0.989)	0.660 (0.606-0.712)	0.991 (0.989-0.993)
Pathological Q waves	AQW	271	3.20	0.2523	0.978 (0.971-0.984)	0.681 (0.619-0.738)	0.643 (0.593-0.690)	0.659 (0.603-0.715)	0.987 (0.985-0.990)	0.629 (0.569-0.686)	0.989 (0.986-0.991)
Sinus tachycardia	ST	238	2.81	0.3545	0.999 (0.998-0.999)	0.958 (0.940-0.973)	0.876 (0.847-0.902)	0.946 (0.917-0.973)	0.994 (0.992-0.995)	0.816 (0.771-0.858)	0.998 (0.998-0.999)
Left anterior fascicular block	LAFB	230	2.71	0.3248	0.994 (0.992-0.995)	0.818 (0.771-0.860)	0.742 (0.696-0.785)	0.800 (0.748-0.852)	0.990 (0.988-0.992)	0.692 (0.637-0.752)	0.994 (0.993-0.996)
Left-axis deviation	LAD	224	2.64	0.2631	0.981 (0.976-0.984)	0.515 (0.448-0.583)	0.562 (0.515-0.609)	0.709 (0.650-0.766)	0.978 (0.975-0.981)	0.466 (0.415-0.516)	0.992 (0.990-0.994)
Sinus bradycardia	BS	221	2.61	0.3799	0.997 (0.996-0.998)	0.902 (0.856-0.937)	0.825 (0.788-0.861)	0.926 (0.891-0.959)	0.992 (0.989-0.993)	0.744 (0.689-0.795)	0.998 (0.997-0.999)
Left bundle branch block	LBBB	220	2.60	0.4493	0.998 (0.996-0.999)	0.943 (0.915-0.967)	0.889 (0.858-0.919)	0.922 (0.882-0.955)	0.996 (0.995-0.997)	0.859 (0.814-0.900)	0.998 (0.997-0.999)
Right bundle branch and left anterior fascicular blocks	RBBB/LAFB	219	2.58	0.4443	0.997 (0.996-0.998)	0.907 (0.865-0.940)	0.829 (0.789-0.864)	0.844 (0.797-0.892)	0.995 (0.993-0.996)	0.815 (0.760-0.862)	0.996 (0.994-0.997)
Primary repolarization abnormalities	PRA	213	2.51	0.2124	0.985 (0.979-0.989)	0.664 (0.598-0.732)	0.649 (0.600-0.698)	0.783 (0.728-0.839)	0.984 (0.981-0.986)	0.555 (0.500-0.609)	0.994 (0.993-0.996)
Atrial fibrillation	AF	178	2.10	0.4494	0.999 (0.998-0.999)	0.949 (0.921-0.974)	0.890 (0.853-0.921)	0.955 (0.920-0.983)	0.996 (0.995-0.997)	0.833 (0.783-0.880)	0.999 (0.998-1.000)
Short PR interval	ShortPR	132	1.56	0.2221	0.985 (0.974-0.992)	0.640 (0.552-0.720)	0.575 (0.508-0.635)	0.691 (0.606-0.762)	0.989 (0.987-0.991)	0.494 (0.424-0.564)	0.995 (0.993-0.997)
Extremity electrode reversal/misplaced precordial electrode(s)	EMPL	94	1.11	0.4472	0.995 (0.989-0.998)	0.689 (0.593-0.788)	0.757 (0.691-0.819)	0.851 (0.774-0.918)	0.996 (0.994-0.997)	0.684 (0.601-0.767)	0.998 (0.997-0.999)
Early repolarization	ER	91	1.07	0.1035	0.984 (0.980-0.988)	0.464 (0.357-0.562)	0.450 (0.360-0.538)	0.451 (0.352-0.557)	0.994 (0.992-0.996)	0.452 (0.352-0.552)	0.994 (0.992-0.996)
Incomplete right bundle branch block	IRBBB	88	1.04	0.1527	0.986 (0.979-0.992)	0.596 (0.493-0.695)	0.532 (0.461-0.599)	0.742 (0.641-0.824)	0.989 (0.987-0.991)	0.416 (0.344-0.491)	0.997 (0.996-0.998)
Paced rhythm	PACE	88	1.04	0.3628	0.999 (0.998-0.999)	0.888 (0.815-0.946)	0.855 (0.802-0.900)	0.967 (0.924-1.000)	0.997 (0.996-0.998)	0.766 (0.690-0.840)	1.000 (0.999-1.000)
Low QRS voltage in peripheral leads	LQRSVp	85	1.00	0.3292	0.989 (0.985-0.992)	0.463 (0.354-0.579)	0.455 (0.383-0.524)	0.799 (0.709-0.879)	0.983 (0.980-0.985)	0.319 (0.256-0.384)	0.998 (0.997-0.999)
Ectopic atrial rhythm	EAR	81	0.96	0.1525	0.987 (0.981-0.991)	0.470 (0.362-0.583)	0.529 (0.451-0.601)	0.754 (0.658-0.842)	0.989 (0.987-0.992)	0.409 (0.331-0.486)	0.998 (0.997-0.999)
Poor-quality ECG	PQT	80	0.94	0.1379	0.956 (0.932-0.975)	0.561 (0.451-0.659)	0.502 (0.406-0.591)	0.549 (0.439-0.658)	0.994 (0.992-0.995)	0.465 (0.366-0.562)	0.996 (0.994-0.997)
Right-axis deviation	RAD	73	0.86	0.1742	0.991 (0.989-0.994)	0.480 (0.366-0.590)	0.456 (0.378-0.528)	0.807 (0.713-0.889)	0.985 (0.982-0.987)	0.319 (0.251-0.385)	0.998 (0.997-0.999)
Right superior axis	RSA	65	0.77	0.1986	0.997 (0.995-0.998)	0.684 (0.559-0.794)	0.650 (0.553-0.741)	0.723 (0.609-0.831)	0.996 (0.995-0.997)	0.592 (0.485-0.700)	0.998 (0.997-0.999)
ST-segment elevation	STE	57	0.67	0.1400	0.981 (0.972-0.988)	0.319 (0.211-0.437)	0.356 (0.245-0.463)	0.386 (0.262-0.510)	0.995 (0.993-0.996)	0.333 (0.220-0.447)	0.996 (0.995-0.997)
Supraventricular tachycardia	SVT	54	0.64	0.3162	0.997 (0.994-1.000)	0.909 (0.837-0.968)	0.858 (0.775-0.929)	0.836 (0.735-0.930)	0.999 (0.999-1.000)	0.883 (0.791-0.961)	0.999 (0.998-1.000)
Right atrial enlargement	RAE	51	0.60	0.0095	0.980 (0.957-0.996)	0.535 (0.386-0.683)	0.563 (0.444-0.672)	0.588 (0.451-0.719)	0.997 (0.996-0.998)	0.544 (0.415-0.678)	0.998 (0.996-0.998)
Atrial flutter	AFL	51	0.60	0.2207	0.996 (0.994-0.998)	0.762 (0.629-0.869)	0.706 (0.590-0.800)	0.687 (0.551-0.818)	0.998 (0.998-0.999)	0.730 (0.600-0.857)	0.998 (0.997-0.999)
Right bundle branch and left posterior fascicular blocks	RBBB/LPFB	48	0.57	0.1605	0.997 (0.993-0.999)	0.658 (0.515-0.797)	0.710 (0.600-0.796)	0.771 (0.641-0.879)	0.998 (0.997-0.999)	0.662 (0.541-0.780)	0.999 (0.998-0.999)
Low QRS voltage in precordial leads	LQRSVc	46	0.54	0.2332	0.981 (0.965-0.990)	0.315 (0.189-0.448)	0.331 (0.226-0.427)	0.546 (0.396-0.692)	0.990 (0.988-0.992)	0.239 (0.154-0.327)	0.998 (0.996-0.999)
Multifocal atrial rhythm	MAR	44	0.52	0.0190	0.956 (0.935-0.974)	0.157 (0.088-0.239)	0.042 (0.000-0.131)	0.024 (0.000-0.075)	1.000 (0.999-1.000)	0.255 (0.000-1.000)	0.995 (0.993-0.996)
Hyperacute T waves	HATW	43	0.51	0.0703	0.981 (0.966-0.992)	0.597 (0.449-0.735)	0.549 (0.393-0.684)	0.417 (0.271-0.558)	1.000 (0.999-1.000)	0.820 (0.650-0.960)	0.997 (0.996-0.998)
Complete atrioventricular block (third-degree)	CAVB	42	0.50	0.2056	0.996 (0.993-0.998)	0.665 (0.504-0.803)	0.605 (0.486-0.718)	0.738 (0.596-0.871)	0.997 (0.995-0.998)	0.517 (0.388-0.659)	0.999 (0.998-0.999)
Junctional rhythm	JR	42	0.50	0.1536	0.993 (0.990-0.996)	0.503 (0.362-0.640)	0.392 (0.254-0.530)	0.358 (0.216-0.514)	0.998 (0.997-0.999)	0.442 (0.278-0.610)	0.997 (0.995-0.998)
Nonspecific intraventricular conduction delay	NSIVCD	42	0.50	0.0413	0.981 (0.971-0.989)	0.269 (0.151-0.392)	0.325 (0.209-0.436)	0.428 (0.280-0.579)	0.994 (0.992-0.996)	0.265 (0.160-0.373)	0.997 (0.996-0.998)
Second-degree atrioventricular block, Mobitz type I (Wenckebach)	MobitzI	41	0.48	0.0733	0.995 (0.992-0.997)	0.549 (0.395-0.694)	0.564 (0.413-0.686)	0.563 (0.394-0.712)	0.998 (0.997-0.999)	0.571 (0.405-0.712)	0.998 (0.997-0.999)
Left posterior fascicular block	LPFB	41	0.48	0.1082	0.997 (0.995-0.998)	0.531 (0.375-0.686)	0.595 (0.489-0.700)	0.804 (0.677-0.925)	0.996 (0.994-0.997)	0.475 (0.367-0.593)	0.999 (0.998-1.000)
2:1 atrioventricular block	2:1AVB	41	0.48	0.1637	0.998 (0.997-0.999)	0.711 (0.559-0.859)	0.696 (0.576-0.804)	0.709 (0.564-0.839)	0.998 (0.998-0.999)	0.689 (0.545-0.833)	0.999 (0.998-0.999)
Escape complex(es)	ESC	40	0.47	0.0058	0.930 (0.886-0.967)	0.130 (0.065-0.222)	0.179 (0.091-0.271)	0.272 (0.143-0.419)	0.992 (0.990-0.994)	0.135 (0.067-0.216)	0.997 (0.995-0.998)
Ectopic atrial tachycardia, unifocal	EAT	37	0.44	0.0641	0.981 (0.969-0.990)	0.317 (0.166-0.460)	0.309 (0.167-0.427)	0.349 (0.189-0.500)	0.996 (0.995-0.997)	0.282 (0.145-0.409)	0.997 (0.996-0.998)
ST-elevation myocardial infarction	STEMI	35	0.41	0.2052	0.991 (0.975-0.999)	0.661 (0.488-0.821)	0.674 (0.545-0.790)	0.744 (0.600-0.886)	0.998 (0.997-0.999)	0.621 (0.471-0.769)	0.999 (0.998-1.000)
Right ventricular hypertrophy	RVH	34	0.40	0.0767	0.997 (0.994-0.999)	0.663 (0.490-0.823)	0.610 (0.436-0.747)	0.496 (0.323-0.656)	1.000 (0.999-1.000)	0.807 (0.621-0.957)	0.998 (0.997-0.999)
ST-segment depression	STD	33	0.39	0.0969	0.989 (0.976-0.997)	0.492 (0.318-0.662)	0.574 (0.419-0.706)	0.602 (0.428-0.762)	0.998 (0.997-0.999)	0.554 (0.386-0.714)	0.998 (0.998-0.999)
Incomplete left bundle branch block	ILBBB	33	0.39	0.1458	0.985 (0.979-0.991)	0.292 (0.155-0.463)	0.290 (0.171-0.404)	0.449 (0.278-0.615)	0.994 (0.992-0.995)	0.217 (0.122-0.317)	0.998 (0.997-0.999)
Second-degree atrioventricular block, Mobitz type II	MobitzII	32	0.38	0.0010	0.961 (0.926-0.985)	0.131 (0.070-0.215)	0.073 (0.000-0.179)	0.064 (0.000-0.162)	0.998 (0.996-0.999)	0.089 (0.000-0.227)	0.996 (0.995-0.998)
Multifocal atrial tachycardia	MAT	32	0.38	0.0312	0.994 (0.990-0.997)	0.475 (0.303-0.648)	0.000 (0.000-0.000)	0.000 (0.000-0.000)	1.000 (1.000-1.000)	0.000 (0.000-0.000)	0.996 (0.995-0.997)
Ventricular preexcitation - Wolf Parkinson White	WPW	32	0.38	0.2741	1.000 (1.000-1.000)	0.966 (0.879-1.000)	0.968 (0.920-1.000)	0.970 (0.895-1.000)	1.000 (1.000-1.000)	0.968 (0.893-1.000)	1.000 (1.000-1.000)
Indeterminate axis	AXIND	30	0.35	0.0116	0.969 (0.948-0.983)	0.138 (0.065-0.247)	0.128 (0.031-0.246)	0.135 (0.032-0.276)	0.997 (0.995-0.998)	0.125 (0.029-0.243)	0.997 (0.996-0.998)
Missing lead(s)	MLEAD	30	0.35	0.0862	0.947 (0.888-0.992)	0.459 (0.264-0.648)	0.441 (0.267-0.603)	0.391 (0.222-0.577)	0.999 (0.998-0.999)	0.518 (0.296-0.714)	0.998 (0.997-0.999)
Pacemaker malfunction	PACEM	29	0.34	0.0054	0.970 (0.942-0.991)	0.438 (0.248-0.636)	0.224 (0.054-0.410)	0.138 (0.029-0.269)	1.000 (0.999-1.000)	0.666 (0.199-1.000)	0.997 (0.996-0.998)
Nonspecific intraventricular block	NSIVB	29	0.34	0.1646	0.963 (0.939-0.981)	0.278 (0.124-0.452)	0.262 (0.127-0.400)	0.274 (0.125-0.435)	0.997 (0.996-0.998)	0.256 (0.120-0.419)	0.998 (0.996-0.998)
Ventricular tachycardia	VT	29	0.34	0.2377	1.000 (1.000-1.000)	0.992 (0.971-1.000)	0.949 (0.882-1.000)	0.965 (0.885-1.000)	1.000 (0.999-1.000)	0.934 (0.833-1.000)	1.000 (1.000-1.000)
Biatrial enlargement	BAE	28	0.33	0.0023	0.971 (0.942-0.994)	0.339 (0.185-0.510)	0.219 (0.057-0.391)	0.179 (0.045-0.333)	0.999 (0.998-0.999)	0.295 (0.083-0.538)	0.997 (0.996-0.998)
Nonsustained ventricular tachycardia	NSVT	28	0.33	0.0073	0.951 (0.881-0.988)	0.300 (0.145-0.467)	0.322 (0.121-0.513)	0.217 (0.074-0.379)	1.000 (0.999-1.000)	0.671 (0.333-1.000)	0.997 (0.996-0.998)
Isorhythmic atrioventricular dissociation	IAVD	28	0.33	0.0080	0.953 (0.928-0.973)	0.084 (0.038-0.151)	0.000 (0.000-0.000)	0.000 (0.000-0.000)	1.000 (1.000-1.000)	0.000 (0.000-0.000)	0.997 (0.995-0.998)
Digitalis effect	DIG	28	0.33	0.0017	0.927 (0.894-0.958)	0.137 (0.050-0.264)	0.000 (0.000-0.000)	0.000 (0.000-0.000)	1.000 (0.999-1.000)	0.000 (0.000-0.000)	0.997 (0.995-0.998)
Accelerated idioventricular rhythm	AIVR	26	0.31	0.0419	0.972 (0.936-0.994)	0.257 (0.125-0.409)	0.111 (0.000-0.267)	0.074 (0.000-0.191)	0.999 (0.999-1.000)	0.250 (0.000-0.667)	0.997 (0.996-0.998)
Brugada syndrome	BrS	22	0.26	0.0080	0.992 (0.976-0.999)	0.672 (0.461-0.854)	0.162 (0.000-0.381)	0.092 (0.000-0.235)	1.000 (1.000-1.000)	0.864 (0.000-1.000)	0.998 (0.997-0.999)
T-wave inversions	INVT	20	0.24	0.1086	0.993 (0.982-0.999)	0.505 (0.278-0.734)	0.365 (0.143-0.593)	0.300 (0.105-0.500)	0.999 (0.999-1.000)	0.493 (0.200-0.800)	0.998 (0.998-0.999)
Sinus pause	SPAUSE	20	0.24	0.0001	0.833 (0.743-0.909)	0.024 (0.008-0.054)	0.041 (0.000-0.130)	0.051 (0.000-0.167)	0.997 (0.995-0.998)	0.036 (0.000-0.118)	0.998 (0.997-0.999)
Dextrocardia	DEXTRO	19	0.22	0.0002	0.998 (0.997-0.999)	0.454 (0.281-0.666)	0.161 (0.000-0.378)	0.105 (0.000-0.273)	1.000 (0.999-1.000)	0.400 (0.000-1.000)	0.998 (0.997-0.999)
Biventricular hypertrophy	BVH	14	0.17	0.0002	0.961 (0.936-0.982)	0.077 (0.018-0.200)	0.160 (0.000-0.353)	0.143 (0.000-0.350)	0.999 (0.998-1.000)	0.198 (0.000-0.500)	0.999 (0.998-0.999)

Supplementary Table 10. Aggregate model performance on the CODE-II-test under two threshold rules: the primary F1-max thresholds and an alternative based on Youden's J (both selected on the CODE-II validation split and applied unchanged to the Test set). Micro- and macro-averaged metrics are reported—Precision, Recall, F1-score, AUROC, and AUPRC—for each rule.

	F1-max thresholds		Youden's J thresholds	
Metric	micro-averaged	macro-averaged	micro-averaged	macro-averaged
Precision	0.632	0.517	0.228	0.182
Recall	0.801	0.563	0.946	0.945
F1-score	0.706	0.512	0.368	0.274
AUROC	0.983	0.978	0.983	0.978
AUPRC	0.776	0.553	0.776	0.553

Supplementary Table 11. Per-class model performance on the CODE-II-test for the three diagnostic classes that produced degenerate metrics under the F1-max rule (precision, recall, and F1 equal to zero). For each class, we report Test-set prevalence (number and %), AUROC and AUPRC, and—under two thresholding rules, F1-max and Youden's J, both selected on the CODE-II validation split and applied unchanged to the Test set—the selected threshold and the resulting Precision, Recall, Specificity, F1-score, and NPV.

CODE statement description	CODE label	Total exams	% of all exams	AUROC	AUPRC	Thresholding rule	Threshold	Precision	Recall	Specificity	F1 score	NPV
Multifocal atrial tachycardia	MAT	32	0.38	0.994	0.452	F1-max	0.0312	0.000	0.000	0.999	0.000	0.996
						Youden's J	3.9976e-08	0.068	1.000	0.948	0.128	1.000
Isorhythmic atrioventricular dissociation	IAVD	28	0.33	0.952	0.074	F1-max	0.0079	0.000	0.000	0.999	0.000	0.997
						Youden's J	7.0004e-08	0.037	0.786	0.933	0.071	0.999
Digitalis effect	DIG	28	0.33	0.926	0.119	F1-max	0.0016	0.000	0.000	0.999	0.000	0.997
						Youden's J	2.9463e-09	0.012	0.964	0.733	0.023	0.999

Supplementary Table 12. Macro-AUROC results for all supervised and pre-trained models on the five PTB-XL tasks—diagnosis, subclass, superclass, form, and rhythm—and on the full CPSC 2018 dataset. All models were trained using 100% of the available training data. For each model, we report the mean macro-AUROC over three runs, with the run-to-run variation shown in parentheses (maximum minus minimum). The table also includes the average macro-AUROC across tasks and the total number of model parameters. The complete set of results, including each model under few-shot training (5% and 10%) on PTB-XL and the improvement relative to its randomly initialized counterpart (when applicable), is provided in Supplementary Data 6 (external Excel file, *Supplementary_Data_6.xlsx*).

	PTB-XL classification tasks					CPSC 2018	Average Macro-AUROC	# Parameters
Model	Diagnosis	Subclass	Superclass	Form	Rhythm	Diagnosis	Avg. AUROC	Params
BiLSTM	0.9153 (0.016)	0.9202 (0.006)	0.9224 (0.001)	0.8493 (0.017)	0.9386 (0.033)	0.9581 (0.008)	0.9173	2,335,660
LSTM	0.9168 (0.004)	0.9161 (0.020)	0.9241 (0.003)	0.8266 (0.065)	0.9602 (0.009)	0.9617 (0.010)	0.9176	908,716
XResNet1d101	0.9142 (0.008)	0.8990 (0.008)	0.9148 (0.002)	0.8248 (0.019)	0.9640 (0.009)	0.9607 (0.005)	0.9129	1,877,292
ResNet1d-Wang	0.9291 (0.007)	0.9312 (0.006)	0.9253 (0.003)	0.8609 (0.011)	0.9460 (0.004)	0.9592 (0.006)	0.9253	450,860
InceptionTime	0.9216 (0.007)	0.9134 (0.014)	0.9251 (0.003)	0.8608 (0.031)	0.9589 (0.006)	0.9615 (0.006)	0.9236	512,684
ResNet1d-Lima	0.9195 (0.010)	0.9171 (0.006)	0.9209 (0.002)	0.8041 (0.016)	0.9009 (0.009)	0.9347 (0.003)	0.8995	7,144,908
ResNet1d-Ribeiro	0.9030 (0.008)	0.9027 (0.008)	0.9166 (0.008)	0.8533 (0.012)	0.9543 (0.004)	0.9481 (0.022)	0.913	6,616,508
ResNet1d-Merl	0.9312 (0.008)	0.9332 (0.003)	0.9247 (0.005)	0.8752 (0.021)	0.9144 (0.017)	0.9446 (0.004)	0.9206	3,871,404
ViT1d-Merl	0.9078 (0.005)	0.9128 (0.005)	0.9094 (0.006)	0.7933 (0.009)	0.8925 (0.018)	0.9337 (0.006)	0.8916	5,481,644
ECG-FM	0.8962 (0.005)	0.9148 (0.019)	0.9163 (0.003)	0.8692 (0.024)	0.9666 (0.001)	0.9694 (0.006)	0.9221	90,916,908
Heartlang	0.9188 (0.005)	0.9181 (0.006)	0.9047 (0.006)	0.8582 (0.003)	0.9323 (0.016)	0.9465 (0.012)	0.9131	39,083,820
HuBERT-Small	0.9113 (0.007)	0.9070 (0.003)	0.9046 (0.001)	0.7948 (0.028)	0.9322 (0.005)	0.9310 (0.006)	0.8968	30,231,724
HuBERT-Base	0.8927 (0.032)	0.9054 (0.008)	0.9040 (0.007)	0.8139 (0.013)	0.9448 (0.011)	0.9362 (0.011)	0.8995	92,831,916
Ours	**0.9399 (0.001)**	**0.9336 (0.001)**	**0.9343 (0.003)**	**0.8932 (0.010)**	**0.9691 (0.001)**	**0.9731 (0.002)**	**0.9405**	7,140,556

Supplementary Table 13. Model sizes and pre-training datasets for baseline ECG models. The table reports the number of parameters (estimated from full-data training on PTB-XL diagnostic classes), the size of the pre-training set (in ECG records or ECG–report pairs), and the corresponding data sources used for model pre-training.

Model	# Parameters	Pre-training Size	Pre-training Sources
ResNet1d-Lima	7,144,908	2,470,424 ECG records	CODE-I
ResNet1d-Ribeiro	6,616,508	2,322,513 ECG records	CODE-I
ResNet1d-Merl	3,871,404	771,693 pairs of ECG records and reports	MIMIC-IV ECG
ViT1d-Merl	5,481,644	771,693 pairs of ECG records and reports	MIMIC-IV ECG
ECG-FM	90,916,908	2,503,136 ECG records	UHN-ECG, MIMIC-IV ECG, CPSC, CPSC-Extra, PTB-XL, Georgia, Ningbo, and Chapman
Heartlang	39,083,820	800,035 ECG records	MIMIC-IV ECG
HuBERT-Small	30,231,724	9.1 million ECG records	CODE-I, CPSC and CPSC-Extra, PTB and PTB-XL, Georgia, Chapman, Ningbo, the partition from the Tianchi Arrhythmia Competition, Shandong Provincial Hospital, MIMIC-IV ECG, and SaMi-Trop
HuBERT-Base	92,831,916	9.1 million ECG records	CODE-I, CPSC and CPSC-Extra, PTB and PTB-XL, Georgia, Chapman, Ningbo, the partition from the Tianchi Arrhythmia Competition, Shandong Provincial Hospital, MIMIC-IV ECG, and SaMi-Trop
Ours	7,140,556	2,735,269 ECG records	CODE-II

Supplementary Table 14. Training and fine-tuning configurations for pre-trained ECG models. For each model, we report the learning rates used under full-data and few-shot scenarios, the input frequency and segment length, and the adopted fine-tuning strategy. When applicable, model-specific exceptions are noted. The same configurations were used for both PTB-XL and CPSC 2018 experiments unless otherwise specified. Input signals shorter than the target input length were symmetrically zero-padded, while longer signals were symmetrically truncated.

	Learning Rate		Input Configuration		Fine-tuning Strategy
Model	Full-data	Few-shot	Frequency (Hz)	Input length	Fine-tuning strategy
ResNet1d-Lima	1e-3	1e-3	400	4,096	Full-tuning
ResNet1d-Ribeiro	1e-3	1e-3	400	4,096	Full-tuning
ResNet1d-Merl	1e-3	1e-3	500	N/A	Full-tuning
ViT1d-Merl	1e-3	1e-3	500	N/A	Full-tuning
ECG-FM	1e-6 for pre-trained models, 1e-5 for models with random initialization	1e-3	500	N/A	Full-tuning
Heartlang	1e-3	1e-3	100	N/A	Full-tuning
HuBERT-Small	1e-5	1e-3, 1e-4	500	2,500	Full-tuning was performed except for the convolutional embedder, as specified in the original paper
HuBERT-Base	1e-5	1e-3, 1e-4	500	2,500	Full-tuning was performed except for the convolutional embedder, as specified in the original paper
Ours	1e-3	1e-3	400	4,096	Full-tuning

Supplementary Table 15. Performance of the baseline and reference ECG models on the CODE-II-open dataset under their best-performing configuration, either trained from scratch or fine-tuned from pre-trained weights. The same evaluation pipeline used for external benchmarking was applied to ensure comparability across experiments. Metrics are reported as the mean macro-AUROC and macro-AUPRC over three independent runs, with the range width (maximum—minimum) shown in parentheses. For reference, the performance of the baseline model trained on the full CODE-II cohort is also reported, denoted as Ours (CODE-II) and highlighted in bold. The complete results, including all individual runs, are provided in Supplementary Data 7 (external Excel file, *Supplementary_Data_7.xlsx*).

Model	Training type	macro-AUROC	macro-AUPRC
BiLSTM	from scratch	0.9372 (0.011)	0.3709 (0.069)
LSTM	from scratch	0.9382 (0.017)	0.3659 (0.083)
XResNet1d101	from scratch	0.9467 (0.005)	0.4184 (0.012)
ResNet1d-Wang	from scratch	0.9405 (0.001)	0.3909 (0.013)
InceptionTime	from scratch	0.9489 (0.003)	0.4168 (0.022)
ResNet1d-Lima	fine-tuned	0.9496 (0.008)	0.4092 (0.030)
ResNet1d-Ribeiro	fine-tuned	0.9531 (0.001)	0.4281 (0.005)
ResNet1d-Merl	fine-tuned	0.9425 (0.002)	0.3917 (0.014)
ViT1d-Merl	fine-tuned	0.9278 (0.002)	0.3451 (0.019)
ECG-FM	fine-tuned	0.9248 (0.003)	0.3292 (0.011)
Heartlang	fine-tuned	0.9125 (0.014)	0.2993 (0.038)
HuBERT-Small	fine-tuned	0.9410 (0.003)	0.3767 (0.027)
HuBERT-Base	fine-tuned	0.9364 (0.004)	0.3562 (0.021)
Ours (CODE-II-open)	from scratch	0.9505 (0.003)	0.4097 (0.012)
Ours (CODE-II)	from scratch	**0.9779**	**0.5608**

Supplementary Table 16. Per-class inter-rater agreement among cardiologists (pairwise). For each CODE diagnostic class, we report exam-level prevalence and cardiologist coverage, including the number of cardiologists who reviewed the class and the number of cardiologist pairs with defined or undefined Cohen's kappa. For each cardiologist pair, agreement is computed on their shared exams (including only pairs with ≥30 shared exams) and summarized across pairs as median [IQR] for the number of shared exams and observed agreement. Cohen's kappa is summarized only over non-degenerate pairs (i.e., excluding pairs whose shared-exam annotations are single-label: all-positive or all-negative), whereas these degenerate pairs are retained in the shared-exam and observed agreement summaries. Exams reviewed by more than two cardiologists contribute to multiple pairwise comparisons (one per cardiologist pair). All 66 classes are shown and ordered by median Cohen's kappa (descending).

			Cardiologist coverage			Inter-rater agreement (pairwise, median [IQR])		
CODE statement description	CODE label	Prevalence (%)	Cardiologists (n)	Pairs with defined kappa	Pairs with undefined kappa	Shared exams	Observed agreement	Cohen's kappa
Prolonged QT interval	LQT	6.87	24	59	1	167 [68, 434]	1.000 [0.986, 1.000]	1.000 [0.919, 1.000]
Premature ventricular complex(es)	PVC	5.31	23	60	2	155 [68, 410]	1.000 [0.990, 1.000]	1.000 [0.921, 1.000]
First-degree atrioventricular block	1AVB	4.74	26	59	1	167 [68, 434]	1.000 [0.987, 1.000]	1.000 [0.891, 1.000]
Right bundle branch block	RBBB	3.93	20	57	3	167 [68, 434]	1.000 [0.995, 1.000]	1.000 [0.939, 1.000]
Premature atrial complex(es)	PAC	3.48	21	52	8	167 [68, 434]	1.000 [0.991, 1.000]	1.000 [0.848, 1.000]
Sinus tachycardia	ST	2.81	23	45	15	167 [68, 434]	1.000 [0.999, 1.000]	1.000 [0.967, 1.000]
Left anterior fascicular block	LAFB	2.71	24	59	3	155 [68, 410]	1.000 [0.994, 1.000]	1.000 [0.891, 1.000]
Sinus bradycardia	BS	2.61	22	53	7	167 [68, 434]	1.000 [0.995, 1.000]	1.000 [0.908, 1.000]
Left bundle branch block	LBBB	2.6	25	54	6	167 [68, 434]	1.000 [0.996, 1.000]	1.000 [0.930, 1.000]
Right bundle branch and left anterior fascicular blocks	RBBB/LAFB	2.58	21	55	5	167 [68, 434]	1.000 [0.997, 1.000]	1.000 [0.934, 1.000]
Atrial fibrillation	AF	2.1	20	47	13	167 [68, 434]	1.000 [1.000, 1.000]	1.000 [1.000, 1.000]
Extremity electrode reversal/misplaced precordial electrode(s)	EMPL	1.11	23	41	21	155 [68, 410]	1.000 [0.997, 1.000]	1.000 [0.747, 1.000]
Paced rhythm	PACE	1.04	19	43	15	180 [64, 441]	1.000 [1.000, 1.000]	1.000 [1.000, 1.000]
ST-segment elevation	STE	0.67	15	34	10	210 [68, 456]	1.000 [0.996, 1.000]	1.000 [0.756, 1.000]
Supraventricular tachycardia	SVT	0.64	15	34	7	230 [75, 463]	1.000 [1.000, 1.000]	1.000 [1.000, 1.000]
Right atrial enlargement	RAE	0.6	13	25	6	232 [76, 438]	1.000 [1.000, 1.000]	1.000 [0.873, 1.000]
Atrial flutter	AFL	0.6	17	31	20	218 [70, 448]	1.000 [1.000, 1.000]	1.000 [1.000, 1.000]
Multifocal atrial rhythm	MAR	0.52	15	24	6	126 [55, 464]	1.000 [0.998, 1.000]	1.000 [0.894, 1.000]
Hyperacute T waves	HATW	0.51	13	24	11	230 [74, 458]	1.000 [0.998, 1.000]	1.000 [0.764, 1.000]
Complete atrioventricular block (third-degree)	CAVB	0.5	14	24	5	81 [61, 465]	1.000 [1.000, 1.000]	1.000 [0.982, 1.000]
Junctional rhythm	JR	0.5	15	25	16	203 [71, 463]	1.000 [0.998, 1.000]	1.000 [0.690, 1.000]
Nonspecific intraventricular conduction delay	NSIVCD	0.5	17	31	18	156 [63, 453]	1.000 [0.998, 1.000]	1.000 [0.878, 1.000]
2:1 atrioventricular block	2:1AVB	0.48	13	22	5	96 [65, 500]	1.000 [1.000, 1.000]	1.000 [0.942, 1.000]
Second-degree atrioventricular block, Mobitz type I (Wenckebach)	MobitzI	0.48	13	22	5	96 [66, 500]	1.000 [0.998, 1.000]	1.000 [0.786, 1.000]
Left posterior fascicular block	LPFB	0.48	13	26	12	142 [64, 426]	1.000 [1.000, 1.000]	1.000 [0.788, 1.000]
ST-elevation myocardial infarction	STEMI	0.41	20	28	11	222 [74, 459]	1.000 [0.999, 1.000]	1.000 [0.766, 1.000]
Right ventricular hypertrophy	RVH	0.4	13	25	10	218 [73, 458]	1.000 [1.000, 1.000]	1.000 [0.865, 1.000]
Second-degree atrioventricular block, Mobitz type II	MobitzII	0.38	11	17	4	154 [71, 520]	1.000 [1.000, 1.000]	1.000 [0.941, 1.000]
Multifocal atrial tachycardia	MAT	0.38	10	18	3	96 [61, 465]	1.000 [1.000, 1.000]	1.000 [1.000, 1.000]
Ventricular preexcitation - Wolf Parkinson White	WPW	0.38	11	17	13	234 [76, 460]	1.000 [1.000, 1.000]	1.000 [1.000, 1.000]
Missing lead(s)	MLEAD	0.35	16	16	8	227 [76, 463]	1.000 [1.000, 1.000]	1.000 [0.985, 1.000]
Pacemaker malfunction	PACEM	0.34	12	16	9	96 [69, 520]	1.000 [1.000, 1.000]	1.000 [0.991, 1.000]
Ventricular tachycardia	VT	0.34	15	29	12	222 [72, 444]	1.000 [1.000, 1.000]	1.000 [0.726, 1.000]
Isorhythmic atrioventricular dissociation	IAVD	0.33	11	17	6	77 [58, 472]	1.000 [0.998, 1.000]	1.000 [0.899, 1.000]
Biatrial enlargement	BAE	0.33	14	18	20	225 [72, 448]	1.000 [1.000, 1.000]	1.000 [0.697, 1.000]
Brugada syndrome	BrS	0.26	11	18	2	125 [71, 520]	1.000 [1.000, 1.000]	1.000 [1.000, 1.000]
Sinus pause	SPAUSE	0.24	9	5	6	154 [92, 540]	1.000 [1.000, 1.000]	1.000 [1.000, 1.000]
T-wave inversions	INVT	0.24	14	11	8	242 [86, 492]	1.000 [1.000, 1.000]	1.000 [0.729, 1.000]
Dextrocardia	DEXTRO	0.22	11	5	2	77 [58, 106]	1.000 [1.000, 1.000]	1.000 [1.000, 1.000]
Biventricular hypertrophy	BVH	0.17	10	4	3	444 [74, 542]	1.000 [1.000, 1.000]	1.000 [0.750, 1.000]
Early repolarization	ER	1.07	19	44	13	154 [68, 431]	1.000 [0.992, 1.000]	0.963 [0.618, 1.000]
Digitalis effect	DIG	0.33	13	16	17	230 [81, 463]	1.000 [1.000, 1.000]	0.961 [0.832, 1.000]
Ectopic atrial tachycardia, unifocal	EAT	0.44	14	24	15	222 [73, 458]	1.000 [0.998, 1.000]	0.951 [0.685, 1.000]
Primary repolarization abnormalities	PRA	2.51	21	47	5	167 [71, 446]	1.000 [0.988, 1.000]	0.939 [0.747, 1.000]
ST-segment depression	STD	0.39	13	24	12	226 [74, 464]	1.000 [0.997, 1.000]	0.937 [0.703, 1.000]
Poor-quality ECG	PQT	0.94	20	39	20	156 [70, 438]	1.000 [0.997, 1.000]	0.932 [0.740, 1.000]
Left-axis deviation	LAD	2.64	25	55	7	155 [68, 410]	1.000 [0.987, 1.000]	0.926 [0.758, 1.000]
Right superior axis	RSA	0.77	16	31	19	155 [64, 460]	1.000 [0.996, 1.000]	0.922 [0.568, 1.000]
Right bundle branch and left posterior fascicular blocks	RBBB/LPFB	0.57	13	19	13	227 [48, 466]	1.000 [0.997, 1.000]	0.917 [0.715, 1.000]
Pathological Q waves	AQW	3.2	24	58	4	155 [68, 410]	0.998 [0.982, 1.000]	0.902 [0.655, 1.000]
Low QRS voltage in peripheral leads	LQRSVp	1.0	15	29	12	203 [71, 463]	1.000 [0.990, 1.000]	0.898 [0.521, 1.000]
Escape complex(es)	ESC	0.47	10	16	3	81 [70, 492]	0.997 [0.986, 1.000]	0.897 [0.824, 1.000]
Incomplete right bundle branch block	IRBBB	1.04	19	38	15	154 [63, 431]	1.000 [0.992, 1.000]	0.856 [0.663, 1.000]
Left atrial enlargement	LAE	10.76	25	60	2	155 [68, 410]	0.977 [0.942, 1.000]	0.853 [0.698, 1.000]
Nonsustained ventricular tachycardia	NSVT	0.33	12	18	15	230 [71, 463]	1.000 [0.998, 1.000]	0.851 [0.680, 1.000]
Nonspecific intraventricular block	NSIVB	0.34	12	16	6	205 [97, 508]	1.000 [0.998, 1.000]	0.839 [0.706, 1.000]
ECG within normal limits for age and sex	NORMAL	40.09	25	56	3	156 [70, 438]	0.932 [0.870, 1.000]	0.838 [0.739, 1.000]
Nonspecific ST-T abnormality	NS-STT	11.43	25	62	0	155 [68, 410]	0.964 [0.936, 1.000]	0.829 [0.658, 1.000]
Short PR interval	ShortPR	1.56	21	48	8	190 [70, 434]	1.000 [0.993, 1.000]	0.821 [0.665, 1.000]
Ectopic atrial rhythm	EAR	0.96	20	36	22	155 [64, 441]	1.000 [0.992, 1.000]	0.803 [0.544, 1.000]
Low QRS voltage in precordial leads	LQRSVc	0.54	16	25	21	142 [68, 410]	1.000 [0.996, 1.000]	0.799 [0.386, 1.000]
Left ventricular hypertrophy	LVH	3.37	22	54	8	155 [68, 410]	0.989 [0.975, 1.000]	0.792 [0.662, 1.000]
Accelerated idioventricular rhythm	AIVR	0.31	7	13	0	154 [69, 465]	0.998 [0.986, 0.999]	0.791 [0.660, 0.940]
Incomplete left bundle branch block	ILBBB	0.39	18	29	24	154 [63, 431]	1.000 [0.997, 1.000]	0.766 [0.653, 1.000]
Right-axis deviation	RAD	0.86	19	42	14	190 [70, 434]	1.000 [0.991, 1.000]	0.748 [0.474, 1.000]
Indeterminate axis	AXIND	0.35	8	14	3	232 [74, 520]	0.995 [0.986, 1.000]	0.701 [0.438, 0.904]

Supplementary Table 17. Stratified model performance on the CODE-II-test. Results are reported by sex, age group, state (Minas Gerais [MG] vs non-MG, where non-MG includes all other Brazilian states represented in CODE-II-test), and comorbidity burden (defined as the presence of zero or at least one of seven pre-specified clinical conditions). For each subgroup, we report the number of exams and its proportion within the corresponding stratum, along with micro- and macro-averaged AUROC and AUPRC. Values are shown as point estimates with 95% confidence intervals (in parentheses) estimated from 1,000 bootstrap resamples at the exam level.

				Micro-averaged		Macro-averaged	
Stratum	Subgroup	Exams (n)	Within-stratum (%)	AUROC	AUPRC	AUROC	AUPRC
Sex	Female	4,871	57.47	0.987 (0.985-0.988)	0.806 (0.796-0.817)	0.981 (0.979-0.984)	0.561 (0.552-0.600)
Sex	Male	3,604	42.53	0.979 (0.977-0.981)	0.739 (0.726-0.752)	0.973 (0.968-0.977)	0.556 (0.551-0.587)
Age Group	18-59	4,966	58.6	0.989 (0.988-0.990)	0.817 (0.806-0.828)	0.983 (0.978-0.987)	0.560 (0.551-0.601)
Age Group	60+	3,509	41.4	0.976 (0.973-0.978)	0.727 (0.713-0.740)	0.970 (0.966-0.973)	0.563 (0.552-0.592)
State	MG	6,334	74.74	0.984 (0.983-0.986)	0.785 (0.776-0.794)	0.977 (0.974-0.980)	0.551 (0.545-0.580)
State	Non-MG	2,141	25.26	0.980 (0.978-0.983)	0.747 (0.730-0.765)	0.979 (0.976-0.982)	0.573 (0.565-0.615)
Comorbidities	0	3,109	36.68	0.986 (0.984-0.988)	0.796 (0.781-0.811)	0.983 (0.980-0.985)	0.572 (0.560-0.620)
Comorbidites	≥ 1	5,366	63.32	0.982 (0.980-0.983)	0.765 (0.756-0.776)	0.974 (0.971-0.978)	0.555 (0.548-0.582)

Supplementary Table 18. Per-class calibration results on the CODE-II-test. For each of the 66 CODE diagnostic classes, we report test prevalence (number of exams and percentage) and two calibration metrics: Expected Calibration Error (ECE) and Brier score. Values are shown as point estimates with 95% confidence intervals (in parentheses) estimated from 1,000 exam-level bootstrap resamples.

CODE statement description	CODE label	Exams (n)	Prevalence (%)	ECE (95% CI)	Brier score (95% CI)
ECG within normal limits for age and sex	NORMAL	3,398	40.09	0.0126 (0.0100-0.0193)	0.0788 (0.0749-0.0825)
Nonspecific ST-T abnormality	NS-STT	969	11.43	0.0126 (0.0088-0.0180)	0.0562 (0.0530-0.0597)
Left atrial enlargement	LAE	912	10.76	0.0136 (0.0105-0.0198)	0.0676 (0.0640-0.0715)
Prolonged QT interval	LQT	582	6.87	0.0097 (0.0076-0.0141)	0.0297 (0.0271-0.0323)
Premature ventricular complex(es)	PVC	450	5.31	0.0259 (0.0235-0.0293)	0.0199 (0.0179-0.0221)
First-degree atrioventricular block	1AVB	402	4.74	0.0045 (0.0037-0.0077)	0.0137 (0.0120-0.0154)
Right bundle branch block	RBBB	333	3.93	0.0045 (0.0037-0.0071)	0.0096 (0.0081-0.0112)
Premature atrial complex(es)	PAC	295	3.48	0.0118 (0.0089-0.0147)	0.0204 (0.0181-0.0228)
Left ventricular hypertrophy	LVH	286	3.37	0.0041 (0.0034-0.0074)	0.0142 (0.0125-0.0161)
Pathological Q waves	AQW	271	3.2	0.0080 (0.0061-0.0112)	0.0166 (0.0143-0.0186)
Sinus tachycardia	ST	238	2.81	0.0031 (0.0023-0.0047)	0.0047 (0.0037-0.0057)
Left anterior fascicular block	LAFB	230	2.71	0.0035 (0.0025-0.0061)	0.0101 (0.0087-0.0117)
Left-axis deviation	LAD	224	2.64	0.0045 (0.0034-0.0077)	0.0163 (0.0145-0.0184)
Sinus bradycardia	BS	221	2.61	0.0049 (0.0040-0.0071)	0.0068 (0.0057-0.0079)
Left bundle branch block	LBBB	220	2.6	0.0028 (0.0022-0.0047)	0.0045 (0.0036-0.0056)
Right bundle branch and left anterior fascicular blocks	RBBB/LAFB	219	2.58	0.0020 (0.0016-0.0041)	0.0063 (0.0052-0.0077)
Primary repolarization abnormalities	PRA	213	2.51	0.0037 (0.0027-0.0068)	0.0126 (0.0109-0.0144)
Atrial fibrillation	AF	178	2.1	0.0039 (0.0029-0.0053)	0.0037 (0.0027-0.0047)
Short PR interval	ShortPR	132	1.56	0.0022 (0.0018-0.0049)	0.0085 (0.0070-0.0101)
Extremity electrode reversal/misplaced precordial electrode(s)	EMPL	94	1.11	0.0033 (0.0025-0.0051)	0.0048 (0.0036-0.0062)
Early repolarization	ER	91	1.07	0.0065 (0.0046-0.0085)	0.0084 (0.0066-0.0101)
Incomplete right bundle branch block	IRBBB	88	1.04	0.0018 (0.0016-0.0041)	0.0063 (0.0051-0.0076)
Paced rhythm	PACE	88	1.04	0.0025 (0.0018-0.0040)	0.0029 (0.0019-0.0040)
Low QRS voltage in peripheral leads	LQRSVp	85	1.0	0.0094 (0.0076-0.0112)	0.0082 (0.0072-0.0093)
Ectopic atrial rhythm	EAR	81	0.96	0.0024 (0.0019-0.0047)	0.0065 (0.0052-0.0079)
Poor-quality ECG	PQT	80	0.94	0.0034 (0.0022-0.0051)	0.0060 (0.0048-0.0074)
Right-axis deviation	RAD	73	0.86	0.0048 (0.0035-0.0066)	0.0066 (0.0054-0.0078)
Right superior axis	RSA	65	0.77	0.0021 (0.0015-0.0038)	0.0039 (0.0028-0.0049)
ST-segment elevation	STE	57	0.67	0.0035 (0.0026-0.0054)	0.0056 (0.0043-0.0070)
Supraventricular tachycardia	SVT	54	0.64	0.0014 (0.0009-0.0023)	0.0014 (0.0008-0.0021)
Right atrial enlargement	RAE	51	0.6	0.0051 (0.0038-0.0067)	0.0050 (0.0037-0.0064)
Atrial flutter	AFL	51	0.6	0.0024 (0.0016-0.0035)	0.0024 (0.0015-0.0033)
Right bundle branch and left posterior fascicular blocks	RBBB/LPFB	48	0.57	0.0026 (0.0016-0.0039)	0.0030 (0.0022-0.0040)
Low QRS voltage in precordial leads	LQRSVc	46	0.54	0.0030 (0.0021-0.0047)	0.0048 (0.0038-0.0059)
Multifocal atrial rhythm	MAR	44	0.52	0.0052 (0.0037-0.0067)	0.0052 (0.0038-0.0067)
Hyperacute T waves	HATW	43	0.51	0.0039 (0.0027-0.0054)	0.0037 (0.0025-0.0050)
Complete atrioventricular block (third-degree)	CAVB	42	0.5	0.0019 (0.0013-0.0031)	0.0026 (0.0017-0.0034)
Junctional rhythm	JR	42	0.5	0.0030 (0.0020-0.0044)	0.0037 (0.0027-0.0049)
Nonspecific intraventricular conduction delay	NSIVCD	42	0.5	0.0038 (0.0025-0.0053)	0.0046 (0.0033-0.0060)
Second-degree atrioventricular block, Mobitz type I (Wenckebach)	MobitzI	41	0.48	0.0032 (0.0022-0.0047)	0.0035 (0.0025-0.0048)
Left posterior fascicular block	LPFB	41	0.48	0.0028 (0.0018-0.0043)	0.0035 (0.0026-0.0047)
2:1 atrioventricular block	2:1AVB	41	0.48	0.0024 (0.0016-0.0035)	0.0025 (0.0016-0.0034)
Escape complex(es)	ESC	40	0.47	0.0045 (0.0031-0.0060)	0.0047 (0.0033-0.0062)
Ectopic atrial tachycardia, unifocal	EAT	37	0.44	0.0032 (0.0021-0.0046)	0.0037 (0.0026-0.0050)
ST-elevation myocardial infarction	STEMI	35	0.41	0.0016 (0.0011-0.0028)	0.0021 (0.0014-0.0030)
Right ventricular hypertrophy	RVH	34	0.4	0.0032 (0.0022-0.0045)	0.0030 (0.0020-0.0041)
ST-segment depression	STD	33	0.39	0.0025 (0.0016-0.0038)	0.0028 (0.0019-0.0039)
Incomplete left bundle branch block	ILBBB	33	0.39	0.0010 (0.0008-0.0025)	0.0032 (0.0024-0.0043)
Second-degree atrioventricular block, Mobitz type II	MobitzII	32	0.38	0.0038 (0.0025-0.0051)	0.0038 (0.0025-0.0051)
Multifocal atrial tachycardia	MAT	32	0.38	0.0038 (0.0026-0.0052)	0.0038 (0.0026-0.0052)
Ventricular preexcitation - Wolf Parkinson White	WPW	32	0.38	0.0002 (0.0001-0.0006)	0.0002 (0.0000-0.0005)
Indeterminate axis	AXIND	30	0.35	0.0034 (0.0021-0.0046)	0.0035 (0.0022-0.0047)
Missing lead(s)	MLEAD	30	0.35	0.0026 (0.0018-0.0040)	0.0028 (0.0019-0.0040)
Pacemaker malfunction	PACEM	29	0.34	0.0034 (0.0021-0.0047)	0.0034 (0.0021-0.0047)
Nonspecific intraventricular block	NSIVB	29	0.34	0.0014 (0.0007-0.0029)	0.0030 (0.0020-0.0042)
Ventricular tachycardia	VT	29	0.34	0.0004 (0.0001-0.0008)	0.0003 (0.0001-0.0006)
Biatrial enlargement	BAE	28	0.33	0.0033 (0.0021-0.0045)	0.0033 (0.0021-0.0046)
Nonsustained ventricular tachycardia	NSVT	28	0.33	0.0032 (0.0020-0.0045)	0.0032 (0.0020-0.0045)
Isorhythmic atrioventricular dissociation	IAVD	28	0.33	0.0033 (0.0021-0.0046)	0.0033 (0.0021-0.0046)
Digitalis effect	DIG	28	0.33	0.0033 (0.0022-0.0046)	0.0033 (0.0022-0.0046)
Accelerated idioventricular rhythm	AIVR	26	0.31	0.0029 (0.0018-0.0041)	0.0030 (0.0018-0.0041)
Brugada syndrome	BrS	22	0.26	0.0026 (0.0015-0.0038)	0.0026 (0.0015-0.0037)
T-wave inversions	INVT	20	0.24	0.0020 (0.0011-0.0029)	0.0020 (0.0011-0.0028)
Sinus pause	SPAUSE	20	0.24	0.0024 (0.0014-0.0035)	0.0024 (0.0013-0.0034)
Dextrocardia	DEXTRO	19	0.22	0.0022 (0.0013-0.0033)	0.0022 (0.0013-0.0033)
Biventricular hypertrophy	BVH	14	0.17	0.0016 (0.0009-0.0026)	0.0017 (0.0009-0.0026)

Supplementary Table 19. Overall calibration performance on the CODE-II-test. We report macro- and micro-averaged Expected Calibration Error (ECE) and Brier score, with 95% confidence intervals estimated from 1,000 exam-level bootstrap resamples (shown in parentheses).

Metric	ECE (95% CI)	Brier score (95% CI)
Macro-averaged	0.0043 (0.0044-0.0048)	0.0087 (0.0084-0.0089)
Micro-averaged	0.0022 (0.0019-0.0025)	0.0087 (0.0084-0.0089)

Supplementary References

[1] Palhares, D. M. F. *et al.* Normal limits of the electrocardiogram derived from a large database of brazilian primary care patients. *BMC Cardiovasc. Disord.* **17**, 152 (2017).

[2] Pinto, M. M., Filho *et al.* Electrocardiographic findings in brazilian adults without heart disease: ELSA-brasil. *Arq. Bras. Cardiol.* **109**, 416–424 (2017).

[3] Mason, J. W. *et al.* Electrocardiographic reference ranges derived from 79,743 ambulatory subjects. *J. Electrocardiol.* **40**, 228–234 (2007).

[4] Rijnbeek, P. R. *et al.* Normal values of the electrocardiogram for ages 16-90 years. *J. Electrocardiol.* **47**, 914–921 (2014).
